# Taxonomic revision of the long-nosed armadillos, Genus *Dasypus* Linnaeus, 1758 (Mammalia, Cingulata)

**DOI:** 10.1371/journal.pone.0195084

**Published:** 2018-04-06

**Authors:** Anderson Feijó, Bruce D. Patterson, Pedro Cordeiro-Estrela

**Affiliations:** 1 Laboratório de mamíferos, Departamento de Sistemática e Ecologia, Centro de Ciências Exatas e da Natureza, Universidade Federal da Paraíba, Campus I, João Pessoa, PB, Brazil; 2 Programa de Pós-Graduação em Ciências Biológicas (Zoologia), Departamento de Sistemática e Ecologia, Centro de Ciências Exatas e da Natureza, Universidade Federal da Paraíba, Campus I, João Pessoa, PB, Brazil; 3 Integrative Research Center, Field Museum of Natural History, Chicago, IL, United States of America; Universidade Federal da Bahia, BRAZIL

## Abstract

*Dasypus* is the most speciose genus of the order Cingulata, including approximately 40% of known living armadillos. Nine species are currently recognized, although comprehensive analyses of the entire genus have never been done. Our aim is to revise the taxonomy of the long-nosed armadillos and properly define the taxa. We examined 2126 specimens of *Dasypus* preserved in 39 different museum collections, including 17 type specimens. Three complementary methods were applied to explore morphological datasets both qualitatively and quantitatively. Qualitative morphological variation in discrete characters was assessed by direct observations of specimens. Linear morphometric variation was based on external data and cranial measurements of 887 adult skulls. The shape and size of the skull was abstracted through two-dimensional geometric morphometric analyses of dorsal, lateral and ventral views of respectively 421, 211, and 220 adult specimens. Our results converge on the recognition of eight living species (*D*. *beniensis*, *D*. *kappleri*, *D*. *mazzai*, *D*. *novemcinctus*, *D*. *pastasae*, *D*. *pilosus*, *D*. *sabanicola*, and *D*. *septemcinctus*), and three subspecies of *D*. *septemcinctus* (*D*. *s*. *septemcinctus*, *D*. *s*. *hybridus*, and a new subspecies from Cordoba described here). Information on type material, diagnosis, distribution, and taxonomic comments for each taxon are provided. We designate a lectotype for *D*. *novemcinctus*; and a neotype for *Loricatus hybridus* (= *D*. *septemcinctus hybridus*).

## Introduction

*Dasypus* is the most speciose genus of the order Cingulata, including approximately 40% of living known armadillo species [1,2,3]. It has the widest distribution of the order, occurring from Argentina to the United States, encompassing the latitudes from 39°S to 40°N [4]. The monophyly of the genus is well supported both by morphological [5,6] and molecular data [7,8,9,10]. *Dasypus* is the only living representative of the subfamily Dasypodinae (or Dasypodidae according to Gibb et al. [10]) and diverged from other armadillos during the Neogene. Its precise divergence time is still debated. Gibb et al. [10], based on a molecular dataset, proposed an estimate of 12.4 Ma (middle Miocene), whereas Castro et al. [5], based on fossil evidence, suggested 3.5 Ma (late Pliocene).

Armadillos of the genus *Dasypus* have unique traits among mammals. They are the only vertebrates known to exhibit obligatory polyembryony, i.e., production of multiple embryos from a single zygote [11,12]. The females of long-nosed armadillos give birth to fixed clonal siblings that vary from two to twelve offspring according to the species [13,14,15]. Moreover, the long-nosed armadillos are the only natural non-human hosts of *Mycobacterium leprae*, the agent of leprosy [16,17]. In addition, they are the natural reservoir of several other pathogens of human interest, such as *Trypanosoma cruzi* and *Leishmania* spp. [15,18]. Due to these peculiar traits, the armadillos of the genus *Dasypus* are widely used as models in biomedical studies (see [19]).

The taxonomy of the long-nosed armadillos has a dynamic and debated history. All six armadillos described by Linnaeus [20] were allocated into the genus *Dasypus*. Later, Lesson [21], Wagler [22], Burmeister [23], Peters [24], Gray [25] and Gray [26] proposed new taxonomic arrangements splitting the armadillos into at least four genera. From the middle of XIX^th^ to the beginning of XX^th^ century, the long-nosed armadillos were largely referred to the genus *Tatu* Blumenbach, 1779 [25,26,27,28,29,30,31], whereas *Dasypus* was restricted to the six-banded armadillo (currently *Euphractus sexcinctus*). When Thomas [32] selected *Dasypus novemcinctus* as type species of *Dasypus* Linnaeus, 1758, he ended prior discussion and since then the long-nosed armadillos have been referred to *Dasypus*.

The number of taxa of *Dasypus* considerably changed during the XIX^th^ century, reaching up to 26 species. The majority of descriptions were based on slight differences evident in single or few specimens (e.g. [24,26]). Lönnberg [33] stated that many of the diagnostic traits of *Dasypus* related taxa show high intraspecific variation. Hamlett [34] recognized only five species (*D*. *novemcinctus*, *D*. *septemcinctus*, *D*. *hybridus*, *D*. *kappleri*, and *D*. *“mazzai”*) plus *Dasypus pilosus*, which he had not examined. Cabrera [35] listed the same six species as Hamlett [34], and also recognized three South American subspecies of *D*. *novemcinctus* and two subspecies of *D*. *kappleri*. In the most recent revision of the genus, Wetzel and Mondolfi [36] recognized six species of *Dasypus* classified into three subgenera: *Hyperoambon* Peters, 1864, *Cryptophractus* Fitzinger, 1856, and *Dasypus* Linnaeus, 1758. In their taxonomic arrangement, Wetzel and Mondolfi [36] included the recently described *Dasypus sabanicola* Mondolfi, 1968 and excluded *Dasypus mazzai* Yepes, 1933, which they considered a synonym of *Dasypus hybridus*. Later, Vizcaíno [37] described *Dasypus yepesi* from northwestern Argentina. Accordingly, Gardner [38] recognized seven species and eight subspecies, as did Wetzel et al. [1]. Unlike the XIX^th^ century, the taxonomy of the long-nosed armadillos over the last decades has had only minor changes. Recently, Feijó and Cordeiro-Estrela [3] split *Dasypus kappleri* into three species, revalidating *D*. *pastasae* and *D*. *beniensis* and highlighting the underestimated diversity in the genus.

Nine species are currently recognized in the genus: *D*. *beniensis*, *D*. *hybridus*, *D*. *kappleri*, *D*. *mazzai*, *D*. *novemcinctus*, *D*. *pastasae*, *D*. *pilosus*, *D*. *sabanicola*, and *D*. *septemcinctus*. Nevertheless, comprehensive analyses of the whole genus have never been done. In this sense, our aim is to revise the taxonomy of the long-nosed armadillos, properly define its taxa, and describe their morphological variation and geographical distribution.

## Materials and methods

### Specimens examined

We examined 2126 specimens of the genus *Dasypus* represented by dry skins, carapaces, isolated osteoderms, and skulls preserved in the following collections: Colección Mamíferos Lillo, Tucumán, Argentina (CML); Museo Argentino de Ciencias Naturales “Bernardino Rivadavia”, Buenos Aires, Argentina (MACN); Museo de La Plata, La Plata, Argentina (MLP); Naturhistorisches Museum, Vienna, Austria (NMW); Colección Boliviana de Fauna, La Paz, Bolivia (CBF); Museo de Historia Natural Noel Kempff Mercado, Santa Cruz, Bolivia (MNK); Universidade Regional de Blumenau, Santa Catarina, Brazil (FURB); Instituto Nacional de Pesquisas da Amazônia, Manaus, Brazil (INPA); Museu de Ciências Naturais da Fundação Zoobotânica, Porto Alegre, Brazil (MCN); Museu de Ciências Naturais da Pontifícia Universidade Católica de Minas Gerais, Belo Horizonte, Brazil (MCN-M); Universidade Luterana do Brasil, Canoas, Brazil (MCNU); Museu de História Natural Capão da Imbuia, Curitiba, Brasil (MHNCI); Museu Nacional da Universidade Federal do Rio de Janeiro, Rio de Janeiro, Brazil (MN); Museu Paraense Emílio Goeldi, Pará, Brazil (MPEG); Museu de Zoologia da Universidade de São Paulo, São Paulo, Brazil (MZUSP); Universidade Estadual do Rio de Janeiro, Rio de Janeiro, Brazil (UERJ); Universidade Federal de Minas Gerais, Belo Horizonte, Brazil (UFMG); Coleção de Mamíferos do Departamento de Sistemática e Ecologia da Universidade Federal da Paraíba, João Pessoa, Brazil (UFPB); Coleção de Mamíferos da Universidade Federal de Pernambuco, Recife, Brazil (UFPE); Coleção de Mamíferos da Universidade de Brasília, Distrito Federal, Brazil (UNB); Instituto Alexander von Humboldt, Villa de Leyva, Colombia (IAVH); Instituto de Ciencias Naturales, Universidad Nacional de Colombia, Bogotá, Colombia (ICN); Museo Ecuatoriano de Ciencias Naturales, Quito, Ecuador (MECN); Museo del Instituto de Ciencias Biológicas de la Escuela Politécnica Nacional, Quito, Ecuador (MEPN); Museo de Zoología, de la Pontificia Universidad Católica del Ecuador, Quito, Ecuador (QCAZ); Musée Zoologique de l’Université et de la ville de Strasbourg, Strasbourg, France (MZS Mam); Staatliches Museum für Naturkunde Stuttgart, Stuttgart, Germany (SMNS); Museum für Naturkunde, Berlin, Germany (ZMB_Mam); Museo Nacional de Historia Natural del Paraguay, Asunción, Paraguay (MNHNP); Museo de Historia Natural, Universidad Nacional Mayor de San Marcos, Lima, Peru (MUSM); Swedish Museum of Natural History (Naturhistoriska Riksmuseet), Stockholm, Sweden (NRM); Museum of Evolution, Uppsala University, Sweden (UUZM); Museo Nacional de História Natural de Montevideo, Montevideo, Uruguay (MNHN); Coleccíon de Mamíferos de la Facultad de Ciencias Naturales, Montevideo, Uruguay (ZVCM); American Museum of Natural History, New York, USA (AMNH); Field Museum of Natural History, Chicago, USA (FMNH); Museo de la Estación Biológica Rancho Grande, Maracay, Venezuela (EBRG); Museo de Biologia, Universidad Central da Venezuela, Caracas, Venezuela (MBUCV); Museo de Historia Natural La Salle, Caracas, Venezuela (MHNLS). A list of all specimens examined including their museum collection numbers and localities is presented in the S1 Appendix.

Examined material included the type specimens of *Dasypus novemcinctus* Linnaeus, 1758, *Dasypus novemcinctus aequatorialis* Lönnberg, 1913, *Dasypus kappleri peruvianus* Lönnberg, 1928, *Dasypus beniensis* Lönnberg, 1942 housed at NRM; *Dasypus novemcinctus fenestratus* Peters, 1864, *Dasypus novemcinctus mexicanus* Peters, 1864, *Dasypus pentadactylus* Peters, 1864 housed at ZMB_Mam; *Dasypus novemcinctus mexianae* Hagmann, 1908 housed at MZS Mam; *Dasypus kappleri* Krauss, 1862 housed at SMNS; *Dasypus sabanicola* Mondolfi, 1968 housed at EBRG; *Dasypus mazzai* Yepes, 1933 housed at MACN; *Dasypus yepesi* Vizcaíno, 1995 housed at MLP; *Dasypus septemcinctus* Linnaeus, 1758 housed at UUZM; and *Cryptophractus pilosus* Fitzinger, 1856 housed at NMW. We also examined photographs of the type specimen of *Tatu pastasae* Thomas, 1901 housed in the Natural History Museum, London, England (BMNH) and *Dasypus peba* Burmeister, 1848 at the Martin-Luther-Universität Halle-Wittenberg, Halle, Germany.

### Nomenclatural acts

The electronic edition of this article conforms to the requirements of the amended International Code of Zoological Nomenclature, and hence the new names contained herein are available under that Code from the electronic edition of this article. This published work and the nomenclatural acts it contains have been registered in ZooBank, the online registration system for the ICZN. The ZooBank LSIDs (Life Science Identifiers) can be resolved and the associated information viewed through any standard web browser by appending the LSID to the prefix “http://zoobank.org/”. The LSID for this publication is: urn:lsid:zoobank.org:pub:F77AD6D7-54C1-457A-8212-9336F1A42C59. The electronic edition of this work was published in a journal with an ISSN, and has been archived and is available from the following digital repositories: PubMed Central, LOCKSS.

### Gazetteer and geographic distribution

Geographic provenance of specimens was obtained from specimen labels. Coordinates are as precise as possible, given this source, and were obtained directly from the labels, from published gazetteers (e.g. [39,40,41,42,43,44,45]), or online databases (e.g., Geonames—http://www.geonames.org/; Global Gazetteer Volume 2.3—http://www.fallingrain.com/world/index.html). In some cases, the exact locality was unavailable or not determinable, so we used the coordinates of the nearest city.

### Morphological approach

Morphological variation was assessed through qualitative and quantitative approaches. Only adult specimens were used in our statistical analyses. Specimens were treated as adults when they exhibited a fused basisphenoid-basioccipital suture, fused dorsal sutures, and a partial or complete permanent dentition [36,46,47]. To obtain a broad overview of the variation present in *Dasypus*, we employed the complementary methods described below.

#### Qualitative data and definition of morphogroups

External and cranial qualitative characters were mainly based on diagnostic traits of species and subspecies (senior and junior synonyms) of the genus *Dasypus* as previously described [3,24,26,31,33,34,36,48,49,50,51,52,53,54,55] and on our observations of trait variation. A list of characters analyzed in this study is presented in the S2 Appendix. Nomenclature of the cranial bones follows Wible and Gaudin [56].

We sorted our sample into ten morphogroups based on a consistent combination of traits that are geographic structured, regardless of the previously attributed latin name of the specimen. The advantage of this approach is that it can reveal the morphological diversity present in the sample, instead of relying on currently recognized species as analytical baselines, which often underestimate actual diversity (e.g., [3,57, 58]). This is especially true for medium and large mammals that are taxonomically poorly studied [3, 57, 58].

We divided these ten groups into two artificial size classes, namely large (from Group 1 to 5) and small (Group 6–10) *Dasypus*. Below we briefly describe the principal diagnostic characters of each morphogroup (for a full description and detailed metric information of them see the “Species Account” section). The following comparative uni- and multivariate analyses were performed using the morphogroups.

The large *Dasypus* include armadillos with skull length varying from 79 to 141 mm. Group 1 includes the largest specimens of the sample (mean total skull length 133.6 mm [n = 8], standard deviation(sd) = 3.5 mm), with externally visible fifth digit on the manus, enlarged projecting scales at the knee, rough scales on the pelvic shield, flattened scales in the proximal rings of the tail, yellowish stripe along the sides of the carapace, poorly differentiated occipital lobe, sigmoid dorsal profile of the skull, smoothly inclined lateral palatine crest, and pentagonal tentorial process of the parietals. Group 2 (mean total skull length 122.7 mm [n = 31], sd = 5.9 mm) resembles Group 1, but with scales of the pelvic shield not uniform in size and texture, flattened scales on the proximal rings of the tail, lateral margin of the palatine with a prominent and thin crest, and a rectangular and prominent tentorial process of the parietals. Group 3 (mean total skull length 128.1 mm [n = 33], sd = 5 mm) lacking a well-defined occipital lobe, exhibits an enlarged projecting scale at the knee, flattened and uniform scales on the pelvic shield, keeled scales on the proximal tail rings, sigmoid dorsal profile of the skull, erect and swollen lateral margin of the palatine, straight posterior border of the palatine, and a rectangular and prominent tentorial process of the parietals. Group 4 consists of medium-sized animals (mean total skull length 97 mm [n = 702], sd = 5.2 mm), with well-defined occipital lobe, four digits on the manus, poorly developed scales at the knee, length of the tail longer than head-and-body length, smooth and flatten scales in the pelvic shield and caudal sheath, sigmoid dorsal profile of the skull, well-marked inflation of the maxilla anterior to the lacrimal, rounded lateral margin of the palatine. Group 5 comprises specimens of medium size (mean total skull length 107.6 mm [n = 5], sd = 4 mm) with carapace totally covered by long, dense yellowish hair, four digits on the manus, well developed occipital lobe, poorly developed scales at the knee, smooth and flattened scales in the caudal sheath, very elongated rostrum, palate and mandible, and diminutive teeth.

The small *Dasypus* vary from 58–76 mm in skull length. Group 6 (mean total skull length 71.9 mm [n = 23], sd = 2.1 mm) includes specimens with 7–9 movable bands, 47–58 scutes on the posterior border of the scapular shield, 46–56 scutes on the 4th movable band, four fingers on the manus, elusive yellowish stripe in the lateral of the carapace, clearly notable occipital lobe, poorly developed of the scales at the knee, length of the tail similar or smaller than head-body length, sigmoid dorsal profile of the skull. Group 7 comprises specimens (mean total skull length 71.3 mm [n = 50], sd = 3.4 mm), with short ears, length of tail smaller than head-body, with 6–7 movable bands, a robust and straightened dorsal profile of the skull, poorly developed inflation of the maxilla anterior to the lacrimal, and rounded lateral margin of the palatine. Group 8 individuals (mean total skull length 65.4 mm [n = 24], sd = 4.6 mm) resembles Group 7, but with longer ears and a fragile and smaller skull. Group 9 includes the smallest specimens (mean total skull length 64.7 mm [n = 6], sd = 1.8 mm) with very short ears, short carapace length, smaller number of scutes in the carapace, a fragile and narrow skull. Group 10 comprises specimens (mean total skull length 73.3 mm [n = 3], sd = 1.2 mm) of a with 8–9 movable bands, 62–66 scutes on the posterior border of the scapular shield, 57–62 scutes on the 4th movable band, four fingers on the manus, notable yellowish stripe in the lateral of the carapace, well-marked occipital lobe, poorly developed of the scales at the knee, length of the tail similar or smaller than head-and-body length, and a sigmoid dorsal profile of the skull.

#### Linear data and analyses

We assessed the linear morphometric variation in our sample through external and cranial data. The external measurements were classified into two categories: standard and carapace. Standard measurements include the traditional measures recorded from freshly dead individuals and were taken from specimen labels. They include six variables: total length (T), head-body length (HB), tail length (TA), hind foot length (HF), ear length (E), and weight (W). When only total length was provided, we subtracted the recorded tail length from it to obtain the values of the head and body length.

**Fig 1:**
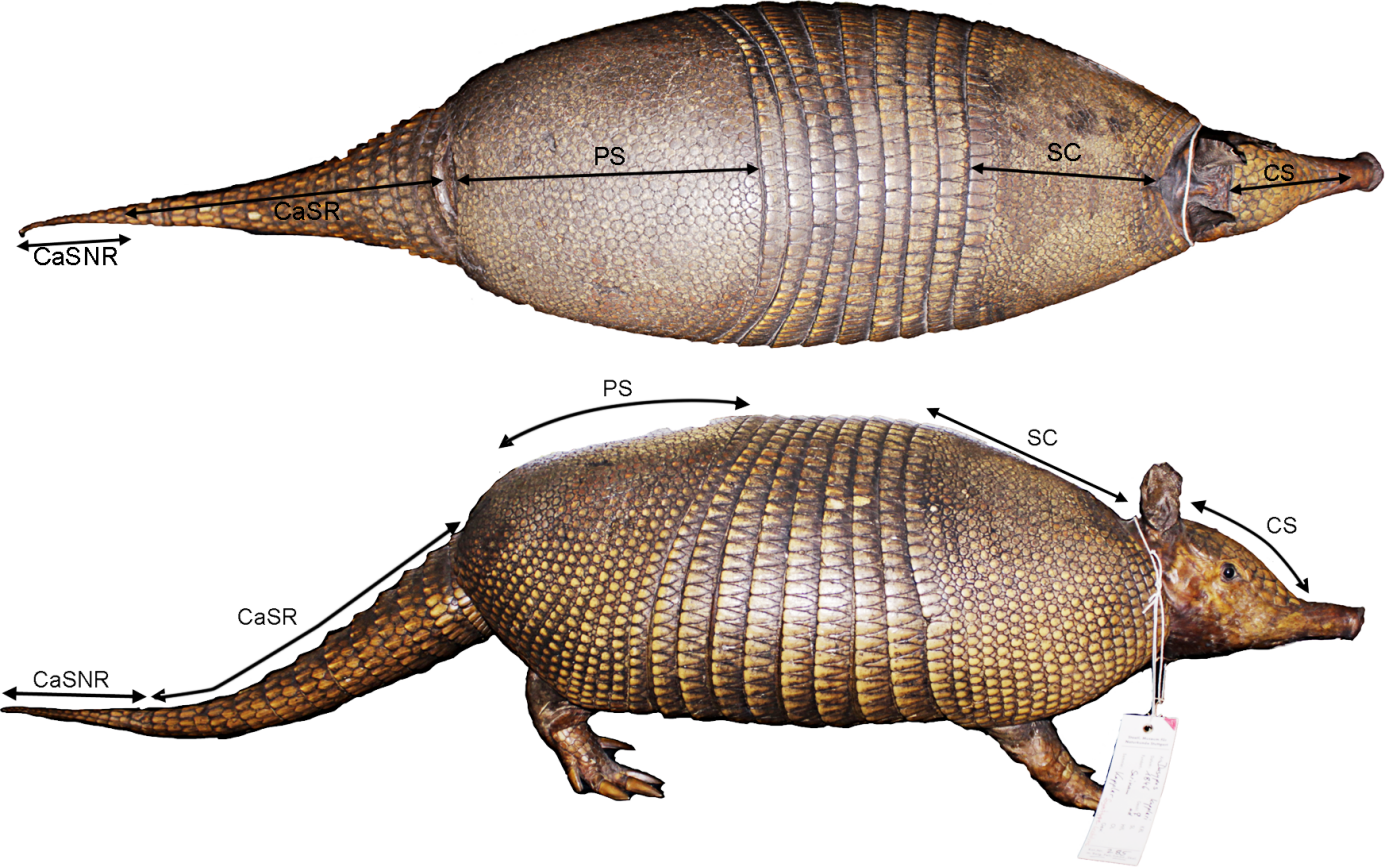
Carapace measurements on *Dasypus kappleri* (SMN 285). CaSR: Dorsal length of the caudal sheath with rings; CaSNR: Dorsal length of the caudal sheath without rings; CS: Dorsal length of the cephalic shield; PS: Dorsal length of the pelvic shield; SC: Dorsal length of the scapular shield. See the text for details.

The five carapace measurements were selected from the characters used previously in diagnosing taxa, either at the specific or subspecific rank. They were taken with a flexible tape measure due to the variable curvature of the carapace. They are defined as follows (Fig 1): Dorsal length of the cephalic shield (CS), shortest distance between the posteriormost and anteriormost point of the cephalic shield; dorsal length of the scapular shield (SC), shortest distance between the anteriormost median point of anterior border of the scapular shield and posteriormost median point of posterior border of the same structure; dorsal length of the pelvic shield (PS), shortest distance between the anteriormost median point of the anterior border of the pelvic shield and posteriormost median point of posterior border of the same structure; dorsal length of the caudal sheath with rings (CaSR): shortest distance between the anteriormost median point of the anterior border of the caudal sheath and the posteriormost median point of the last distal ring (i.e., two rows of scutes organized in concentric rings); dorsal length of the caudal sheath without rings (CaSNR), shortest distance between the anteriormost median point of the anterior portion of the tail without ring and the tip of the tail. We also computed the ratios of the PS to SC and CaSR to CaSNR to reflect proportionality. In addition, we scored the number of scutes on the posterior border of the scapular shield (SSS), on the third and fourth movable bands (3thMB and 4thMB respectively), and the number of tail rings (TR).

External measurements were explored through univariate analyses due to the large number of incomplete specimens (i.e., with one or more variables missing data). Descriptive statistics (mean, standard deviation, range) and Box-and-Whisker plots were used to summarize the metric variance present in each species and to detect putative diagnostic measures.

**Fig 2:**
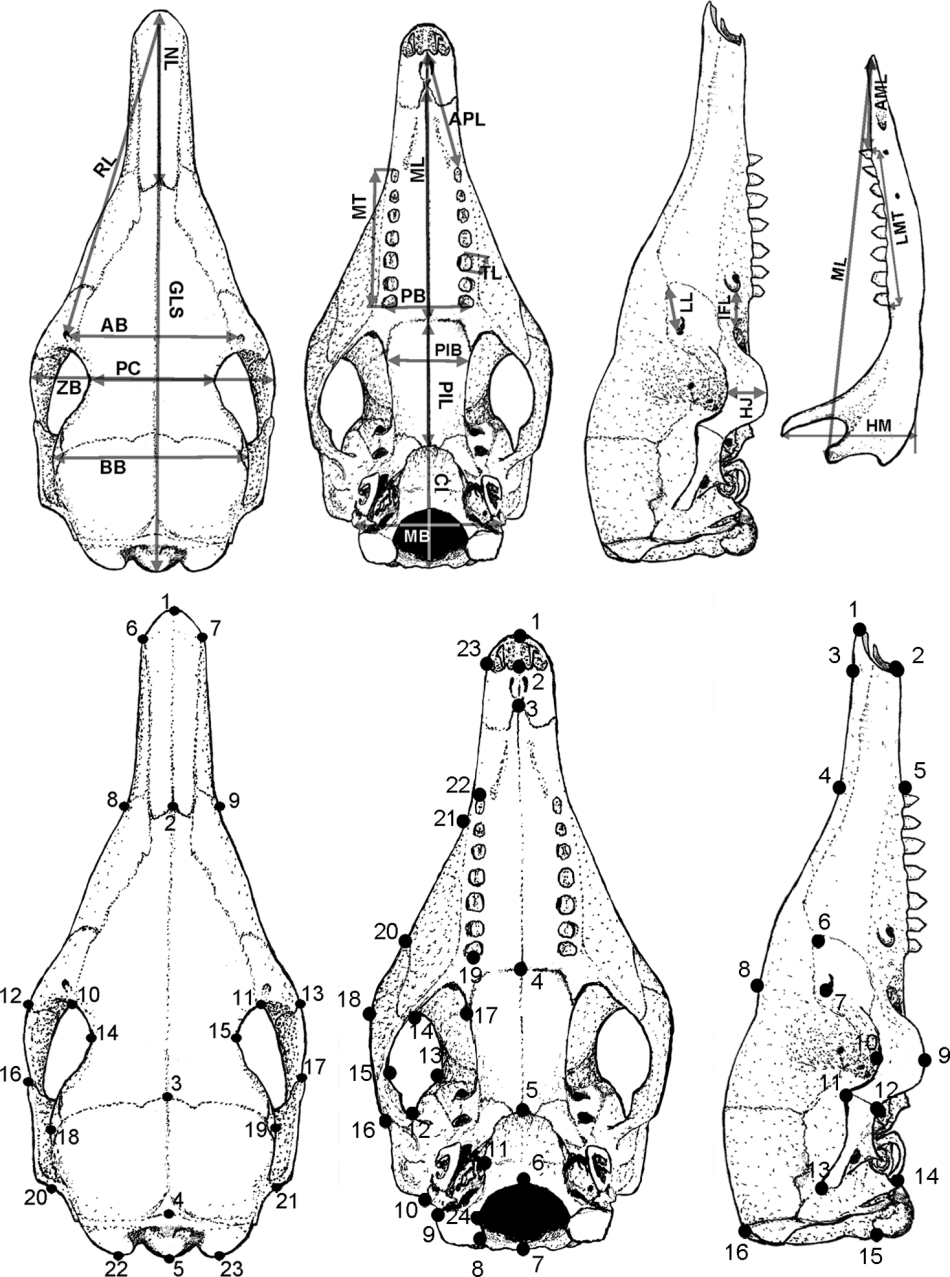
**Top: Scheme of the cranial measurements used in this study and defined in the text. Bottom: Location of landmarks of the dorsal, ventral and lateral views of the skull used in this study. See S3 Appendix for landmarks definition.** Illustration: Fernando Heberson Menezes.

Twenty-four cranial measurements of 887 adult skulls were taken with digital calipers to the nearest 0.01 mm (Fig 2) following the criteria described by Feijó and Cordeiro-Estrela [3]. They are: greatest length of skull (GLS), condylobasal length (CB), anterior palatal length (APL), palatal length (PL), maxilla length (ML), palatine Length (PIL), infraorbital canal length (ICL), maxillary toothrow length (MT), nasal length (NL), lacrimal length (LL), rostral length (RL), anteorbital breadth (AB), tooth length (TL), palatal breadth (PB), palatine breadth (PIB), postorbital constriction (PC), braincase breadth (BB), zygomatic breadth (ZB), mastoid breadth (MB), height of jugal bone (HJ), mandible length (MAL), mandibular toothrow length (LMT), anterior mandibular length (AML), and height of mandible (HM).

Missing values were estimated using the Amelia package [59] in R software [60]. To assess the reliability of the estimated values, we compare, for each variable, the distribution of the inferred and observed values. All variables show similar distribution patterns between these two set of values, indicating an accurate estimation [59]. The raw data were log-transformed prior to all analyses to normalize the distribution of the measurements and to equalize variances in the dataset [61,62]. Sexual dimorphism was tested via two-way ANOVA with interaction between sex and species.

We performed two multivariate analyses that do not require *a priori* groupings: Principal component analysis (PCA) and an unsupervised model-based clustering using Gaussian mixtures analysis via Mclust 5.0.2 package [63,64]. In addition, we performed a Discriminant Analysis Through Eigenvalue Decomposition [64] and the misclassification rate of the pre-established morphogroups was assessed by a leave-one-out cross-validation analysis using the package Mclust 5.0.2 [63].

#### Geometric data and analyses

The shape and size of the skull was abstracted through 23 landmarks in dorsal view of 421 adult specimens, 24 landmarks in ventral view of 220 specimens, and 16 landmarks in lateral view of 211 specimens (Fig 2). All photos were taken following the same standardized protocol. Skulls were photographed with a Canon EOS Rebel t3i digital camera using an 18–55mm lens set at 18 mm focal length. The camera was positioned perpendicular to the table on which each skull rested at a distance of 195 mm from the focal plane. Landmarks were recorded using tpsDig version 2.17 [65]. Coordinates of each landmark were aligned, and the effects of location, orientation and scale were removed through a Generalized Procrustes Analysis [66] using the package geomorph version 3.0.3 [67].

PCA was carried out using the variance-covariance matrix of generalized least-squares superimposition residuals. Pairwise Procrustes distance was used to compare the mean shape between morphogroups using the software MorphoJ version 1.05 [68], and the results were evaluated with a permutation test (10,000 iterations). The misclassification rate of the morphogroups was assessed by a cross-validation analysis through the canonical variate analysis using the Morpho package [69]. The remarkable size variation among *Dasypus* species leads one to predict an allometric effect in our sample (p = 0.01; R^2^ = 0.35; Procrustes ANOVA analysis using geomorph package). Nevertheless, because our aim is to evaluate our “morphogroups” in a taxonomic context [70], specifically regarding species status, and considering size is a fundamental and ecologically informative trait, we do not disentangle shape variation from size effects in our analyses.

## Results

### Geographical sampling and distribution of the morphogroups

**Fig 3:**
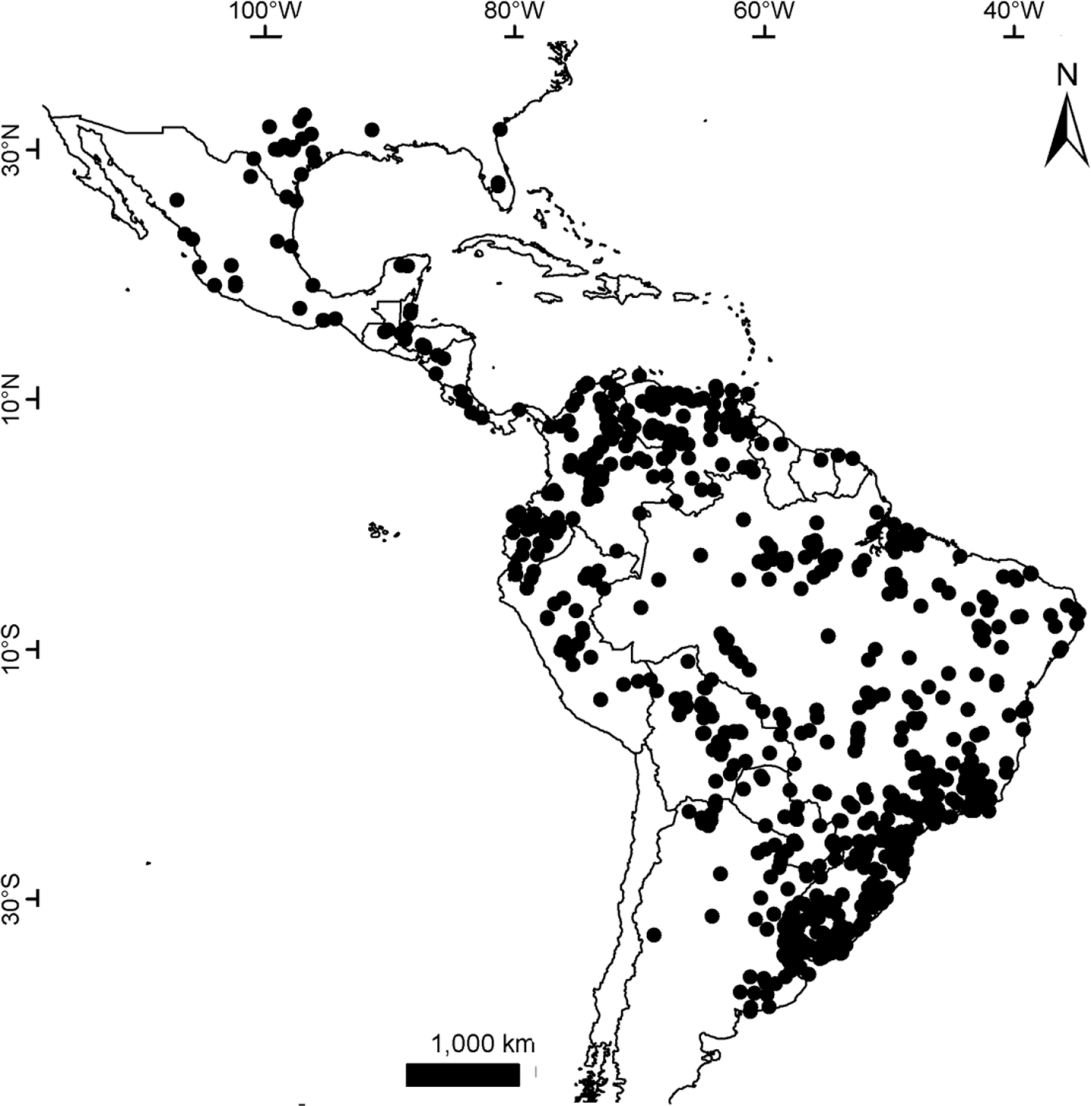
Localities (dots) of the *Dasypus* specimens examined in this study.

**Fig 4:**
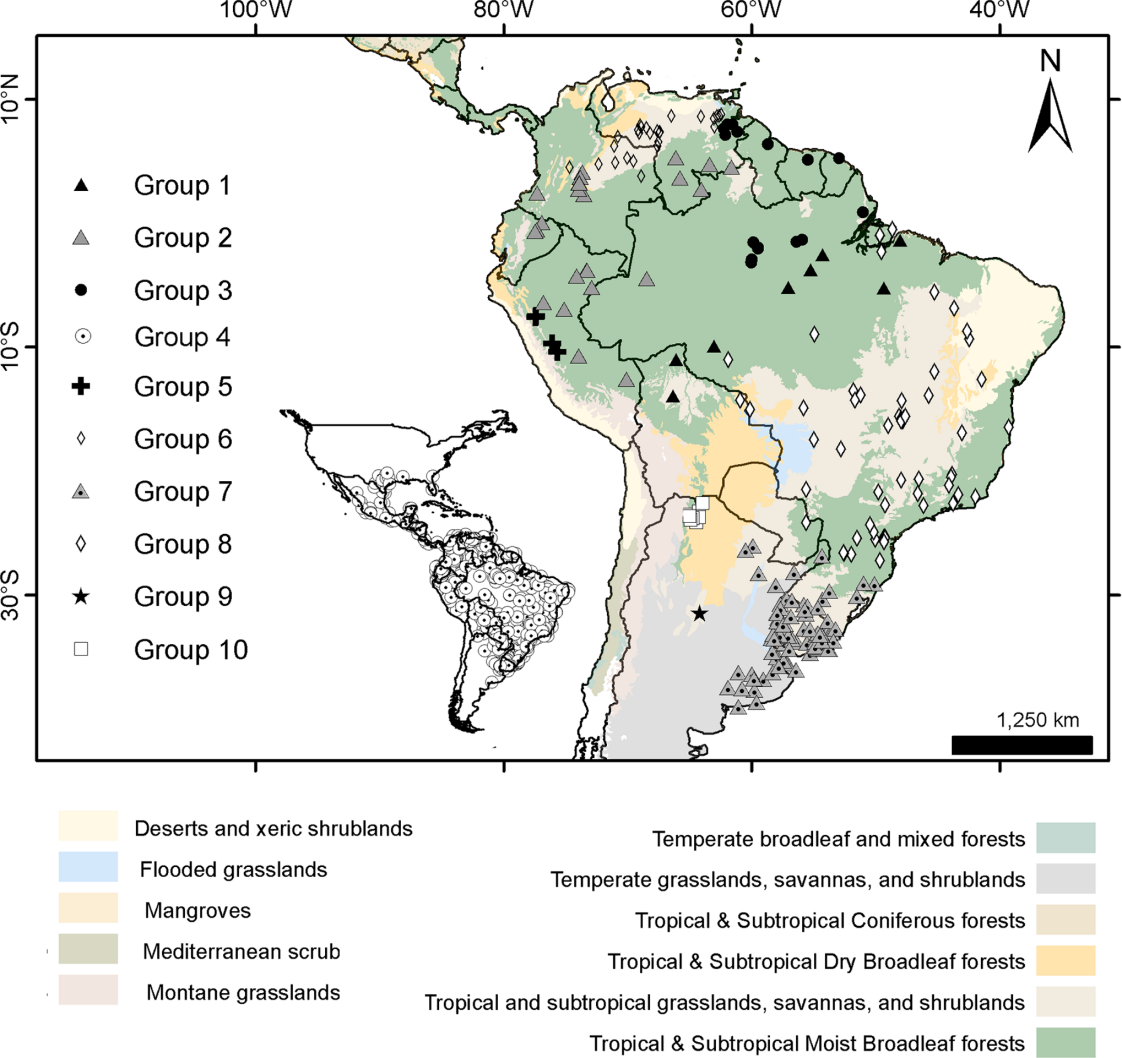
Distribution of the ten morphogroups of *Dasypus*.

The geographic records of *Dasypus* specimens show a Panamerican distribution, from central Argentina to central United States of America (Fig 3). A total of 1097 localities were represented by the specimens examined (S1 Appendix).The ten predefined morphogroups (see Material and Methods) show a mostly non-overlapping distribution (except for Group 4). The “large *Dasypus”* (Groups 1 to 5) are represented mostly by forest dwellers, while the “small *Dasypus”* (Groups 6 to 10) inhabit mainly dry forest and savannas of South America (Fig 4).

### Analyses

All 24 cranial measurements showed no sexual dimorphism and no interaction with the species factor (*P* > 0.05). Therefore, in the following analyses, we pooled together males and females and specimens of undetermined gender.

#### Linear analyses

**Fig 5:**
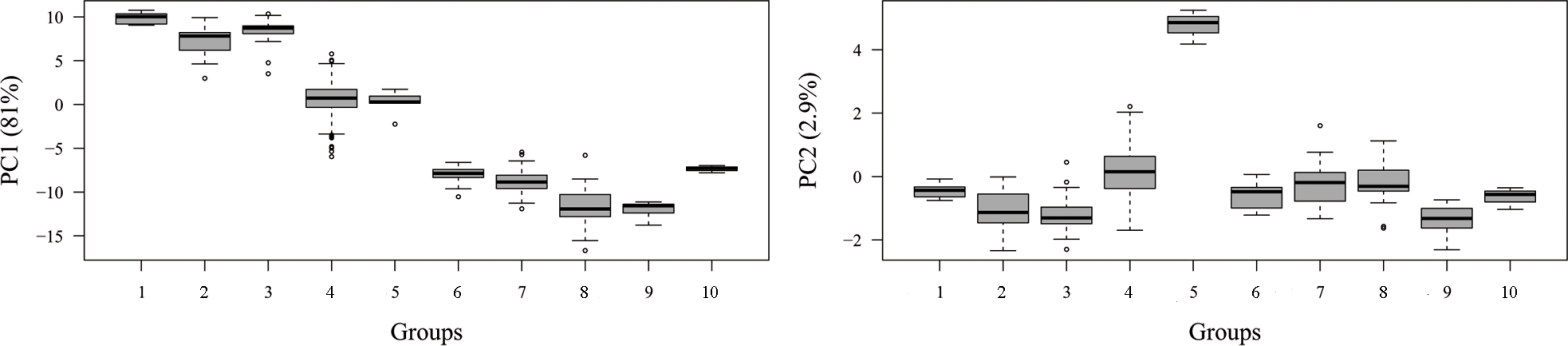
**Boxplots of the scores on PC1 (left) and PC2 (right) of ten morphogroups of *Dasypus***. Boxes delimit the 1^st^ and 3^rd^ quantiles, the heavy midline the median, and whiskers correspond to respective quantiles -/+ 1.5 IQR (interquartile range). Outliers are represented by circles.

The first principal component (PC1) of log-transformed linear skull measurements from the 887 adults explained 81% of overall variation, while the second principal component (PC2) explained only 2.9% (Table 1). We interpret the first component as a measure of skull size, because all loadingss are negative and with similar magnitude [59], and there is a Pearson’s correlation of 0.97 between PC1 and total length of the skull. PC2 summarizes the robustness of the skull, judged from the fact that width-related measurements have negative loadings and length-related measurements show positive loadings (Table 1). Accordingly, we use the scores of both components in a boxplot to visualize differences in size and robustness among groups (Fig 5). PC1 scores form three well defined clusters. Groups 1, 2 and 3 show the highest values of the first principal component; Groups 4 and 5 show intermediate values, whereas the smallest values cluster specimens of Groups 6 through 10. For PC2 scores, there is considerable overlap among groups, except for Group 5, which can be easily differentiated by its high positive scores. It is also noteworthy that, among the “small *Dasypus*” (Groups 6 to 10), Group 9 shows the smallest scores on the second principal component (Fig 5).

**Table 1:** Loadings of the first two principal components extracted from the 887 log-transformed skull measurements.

Variables	PC1	PC2
GLS	-0.2238	-0.0584
CB	-0.2238	-0.0641
APL	-0.2044	-0.3381
PL	-0.2218	-0.0411
ML	-0.2149	-0.1527
PIL	-0.1777	0.3399
IFL	-0.1410	-0.4330
MT	-0.2063	0.0564
NL	-0.2082	-0.2028
LL	-0.2019	0.0191
RL	-0.2216	-0.1008
AB	-0.2165	-0.0182
TL	-0.1687	0.3640
PB	-0.1745	0.2346
PIB	-0.1963	0.3579
PC	-0.2090	-0.0037
BB	-0.2138	0.1303
ZB	-0.2174	0.0631
MB	-0.2157	0.0837
HJ	-0.1901	0.0867
MAL	-0.2235	-0.0858
AML	-0.1972	-0.3516
LMT	-0.2081	0.0836
HM	-0.1993	0.0769
**Variance (%)**	**81**	**2.9**

The unsupervised clustering analysis yielded seven clusters composed either by mixed morphogroups or with a given morphogroup split into two or more clusters. The misclassification rate of the discriminant cross-validated analysis was 3.9%; all specimens from Groups 1 and 5 were correctly classified, while Groups 9 and 10 had the highest misclassification percentages, 57.1% and 66.7%, respectively (Table 2).

**Table 2:** Classification of morphogroups resulting from the cross-validated discriminant analysis from linear measurements of the skull of *Dasypus*, with sample size (N), correct classification percentage (Correct %).

			Cross-validation Classification									
GROUPS	N	% Correct	1	2	3	4	5	6	7	8	9	10
**1**	8	100	8	-	-	-	-	-	-	-	-	-
**2**	31	83.9	-	26	5	-	-	-	-	-	-	-
**3**	33	91	-	3	30	-	-	-	-	-	-	-
**4**	702	99.3	-	-	-	697	-	1	1	-	-	-
**5**	5	100	-	-	-	-	5	-	-	-	-	-
**6**	23	95.7	-	-	-	-	-	22	1	-	-	-
**7**	50	88	-	-	-	-	-	3	44	3	-	-
**8**	25	72	-	-	-	1	-	-	6	16	2	-
**9**	7	42.9	-	-	-	-	-	-	3	1	3	-
**10**	3	33.4	-	-	-	-	-	2	-	-	-	1

#### Geometric analyses

Regarding the shape of the skull, permutation tests with 10,000 randomization of pairwise Procrustes distances show that most groups are statistically different from each other, except for Group 6 and Group 10, Group 7 and Group 9, and Group 9 and Group 10 in dorsal and ventral views. Group 8 does not differ from Group 9 in any view (Table 3).

**Table 3:** Pairwise distances between mean shape of morphogroups. Numbers in boldface represent significant values (p<0.05 based on a permutation test with 10,000 randomizations).

Dorsal Shape									
	Group 1	Group 2	Group 3	Group 4	Group 5	Group 6	Group 7	Group 8	Group 9
Group 2	**0.042**								
Group 3	**0.030**	**0.025**							
Group 4	**0.051**	**0.039**	**0.041**						
Group 5	**0.088**	**0.078**	**0.085**	**0.110**					
Group 6	**0.095**	**0.081**	**0.082**	**0.050**	**0.153**				
Group 7	**0.109**	**0.088**	**0.094**	**0.061**	**0.156**	**0.031**			
Group 8	**0.118**	**0.096**	**0.103**	**0.071**	**0.163**	**0.035**	**0.019**		
Group 9	**0.117**	**0.097**	**0.102**	**0.070**	**0.165**	**0.032**	0.018	0.016	
Group 10	**0.105**	**0.088**	**0.091**	**0.058**	**0.159**	0.019	**0.024**	**0.030**	0.028
Lateral shape									
	Group 1	Group 2	Group 3	Group 4	Group 5	Group 6	Group 7	Group 8	Group 9
Group 2	**0.038**								
Group 3	**0.035**	**0.017**							
Group 4	**0.076**	**0.065**	**0.067**						
Group 5	**0.099**	**0.086**	**0.089**	**0.113**					
Group 6	**0.106**	**0.092**	**0.095**	**0.035**	**0.136**				
Group 7	**0.130**	**0.116**	**0.120**	**0.060**	**0.150**	**0.042**			
Group 8	**0.135**	**0.121**	**0.126**	**0.064**	**0.155**	**0.042**	**0.018**		
Group 9	**0.134**	**0.118**	**0.123**	**0.063**	**0.155**	**0.040**	**0.027**	0.026	
Group 10	**0.100**	**0.091**	**0.092**	**0.031**	**0.138**	**0.027**	**0.045**	**0.047**	**0.045**
Ventral shape									
	Group 1	Group 2	Group 3	Group 4	Group 5	Group 6	Group 7	Group 8	Group 9
Group 2	**0.036**								
Group 3	**0.037**	0.016							
Group 4	**0.059**	**0.063**	**0.063**						
Group 5	**0.101**	**0.095**	**0.097**	**0.115**					
Group 6	**0.091**	**0.093**	**0.092**	**0.039**	**0.141**				
Group 7	**0.110**	**0.108**	**0.108**	**0.061**	**0.152**	**0.034**			
Group 8	**0.112**	**0.109**	**0.110**	**0.067**	**0.156**	**0.039**	**0.022**		
Group 9	**0.118**	**0.117**	**0.118**	**0.069**	**0.162**	**0.039**	0.020	0.025	
Group 10	**0.090**	**0.092**	**0.092**	**0.039**	**0.145**	0.015	**0.038**	**0.041**	**0.040**

**Fig 6:**
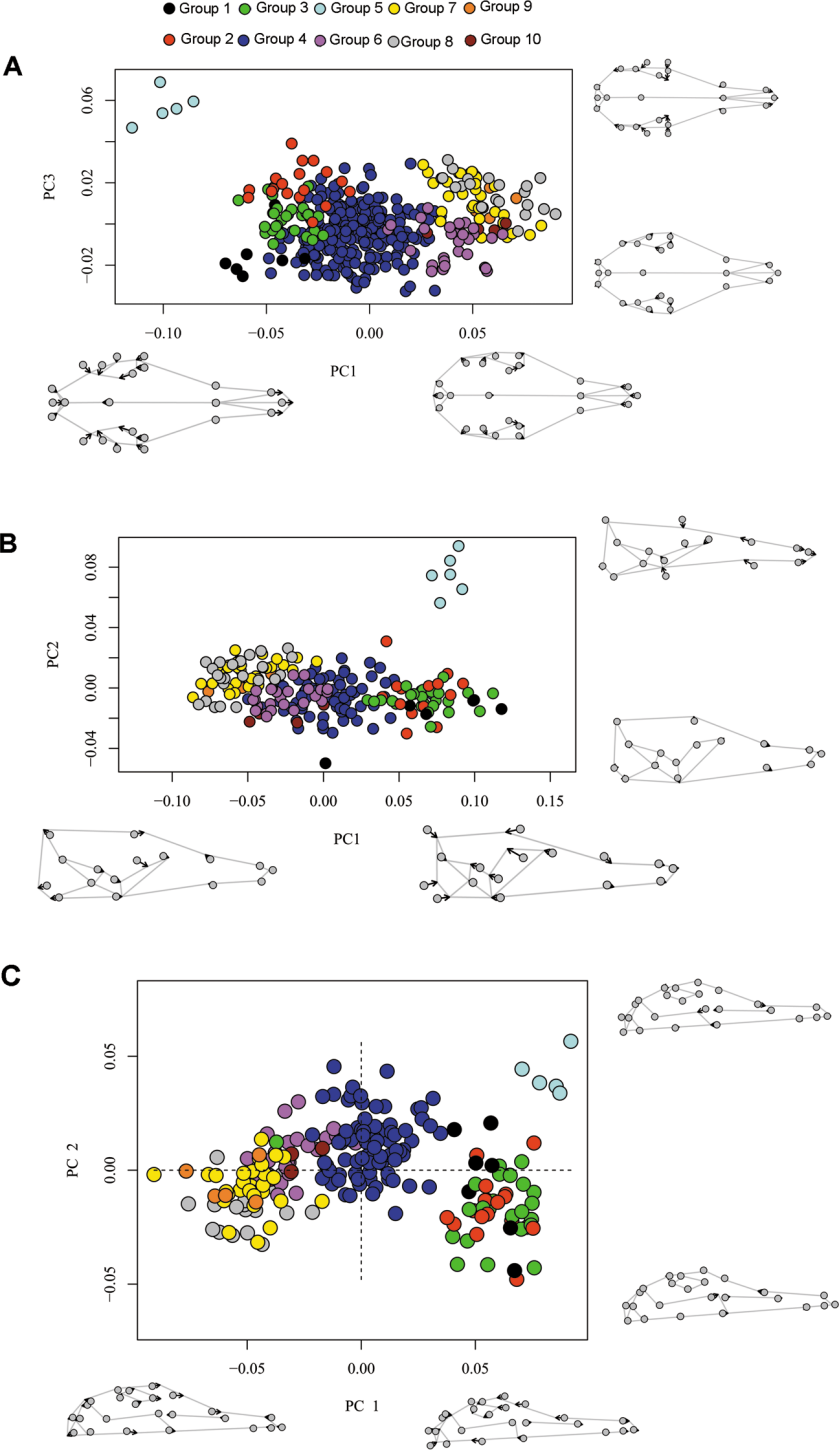
Scatterplot of principal components of shape data of ten morphogroups of *Dasypus*. (A) Dorsal view (B) right lateral view (C) right ventral view. Deformation shape at extreme values of PC1 and PC3 axes (A) or PC1 and PC2 (B, C) are shown. Gray dots represent the mean shape and arrows represent the changes in the landmarks position along the PCs as the score changes.

PC1, PC2 and PC3 of the dorsal shape coordinates of 421 adults explained 48.9%, 16.5% and 9% of shape variance respectively; together, the first three principal components sum to 74.4% of dorsal shape variation. Specimens with positive scores on PC1 and PC2 tend to present shorter nasals and larger braincases than specimens with negative scores on these axes, whereas specimens with positive scores on PC3 exhibit larger nasals. A plot of PC1 and PC3 depicts a clear separation of Group 5 from all others in the dorsal view (Fig 6A). Along PC1, there are three main clusters, one with Groups 1, 2, 3 with negative scores on the axis, one with Group 4 centrally located, and a third cluster with strongly positive scores for Groups 6 to 10. Along the PC3 axis, specimens of Groups 1, 2 and 3 are grouped into a distinct cluster each, Group 1 showing the most negative values, Group 3 with intermediate scores, and Group 2 with the most positive values. The PC3 axis also differentiated Group 6 from Groups 7, 8, and 9.

In lateral view, PC1, PC2 and PC3 account for 62.2%, 8.17% and 6.5% of shape variance respectively; together, the first three principal components represent 76.8% of the lateral shape variation. Specimens with positive scores on PC1 tend to exhibit larger jugal and lachrymal bones and a more markedly sigmoidal dorsal profile of the skull in comparison to specimens with negative scores on the same axis, while specimens with positive scores on PC2 exhibit a shorter, more slender skull and a shorter jugal (Fig 6B). Group 5 is clearly separated from all others along PC2. Specimens of the Group 6 clearly separated from Groups 7, 8 and 9, but overlaps with Group 4. Groups 7, 8 and 9 show a large overlap on both PC1 and PC2.

In ventral view, PC1 accounted for 52.9% of overall variation, PC2 explained 9.4% and PC3 explained 7.6%; together, the first three principal components represent 69.9% of ventral shape variation. Specimens with positive scores on PC1 tend to exhibit narrower orbit and jugal bones, a longer and narrower rostrum, a longer palantine and a shorter toothrow in comparison with specimens with negative scores on the same axis. Along PC2, the differences are mainly related to palatine breadth and lateral expansion of the maxilla (Fig 6C). Groups 1, 2, and 3 are clearly separated from other groups but overlap with each other. Group 5 is totally separated with highly positive scores on PC2 and PC1. Among the “small *Dasypus*”, specimens of Group 8 show more negative values than those of Group 7 on the PC2, but without a clear separation.

The misclassification percentages of the cross-validated discriminant analysis based on dorsal, lateral, and ventral shape data were respectively 8.1%, 14.7%, and 6.9%. All specimens of Group 5 were correctly classified in the dorsal and ventral view. In the dorsal view, the highest rates of correct classification were in decreasing order for Group 6, Group 4, Group 2, and Group 3, whereas the lowest rates were for Group 9, Group 10, and Group 8, respectively. In lateral view, the highest rates of correct classification were for Group 3, Group 7, and Group 4, and the lowest rates were for Group 9 and Group 10. In the ventral view, the highest rates of correct classification were for Group 1, Group 9, Group 4, Group 2, and Group 7, while the lowest was for Group 10 (Table 4).

**Table 4:** Results of the leave-one-out cross-validation analysis based on dorsal, lateral and ventral shape with the percentages of correctly classified specimens.

			**Dorsal Shape–Specimen classification per morphogroup**										
**Groups**	**N**	**Correct (%)**	**1**	**2**	**3**	**4**	**5**	**6**	**7**	**8**	**9**	**10**	**None**
1	7	85.7	6		1								
2	19	89.5		17	2								
3	26	88.9	1	1	23								1
4	269	95.5		2	2	259		5				2	1
5	5	100					5						
6	29	96.5						28				1	
7	33	81.8							27	3	1	1	1
8	21	76.2							2	16			3
9	6	66.7							1		4		1
10	6	66.7						1	1			4	
			**Lateral Shape–Specimen classification per morphogroup**										
**Groups**	**N**	**Correct (%)**	**1**	**2**	**3**	**4**	**5**	**6**	**7**	**8**	**9**	**10**	**None**
1	5	80	4										1
2	14	85.7		12	1								1
3	24	92		1	22								1
4	68	90				61		4				1	2
5	5	83.3					4						1
6	25	84				2		80				2	
7	30	90.3							27	1	1		1
8	20	65.2							2	13	2	1	2
9	5	80								1	4		
10	4	60				1						2	1
			**Ventral Shape—Specimen classification per morphogroup**										
**Groups**	**N**	**Correct (%)**	**1**	**2**	**3**	**4**	**5**	**6**	**7**	**8**	**9**	**10**	**None**
1	7	100	7										
2	18	94.4		17	1								
3	25	92		1	23						1		
4	80	96.2				77							3
5	5	100					5						
6	26	84.6						22				3	1
7	30	93.3							28	1			1
8	20	90							1	18			1
9	5	100									5		
10	4	75						1				3	

#### Carapace measurements

**Fig 7:**
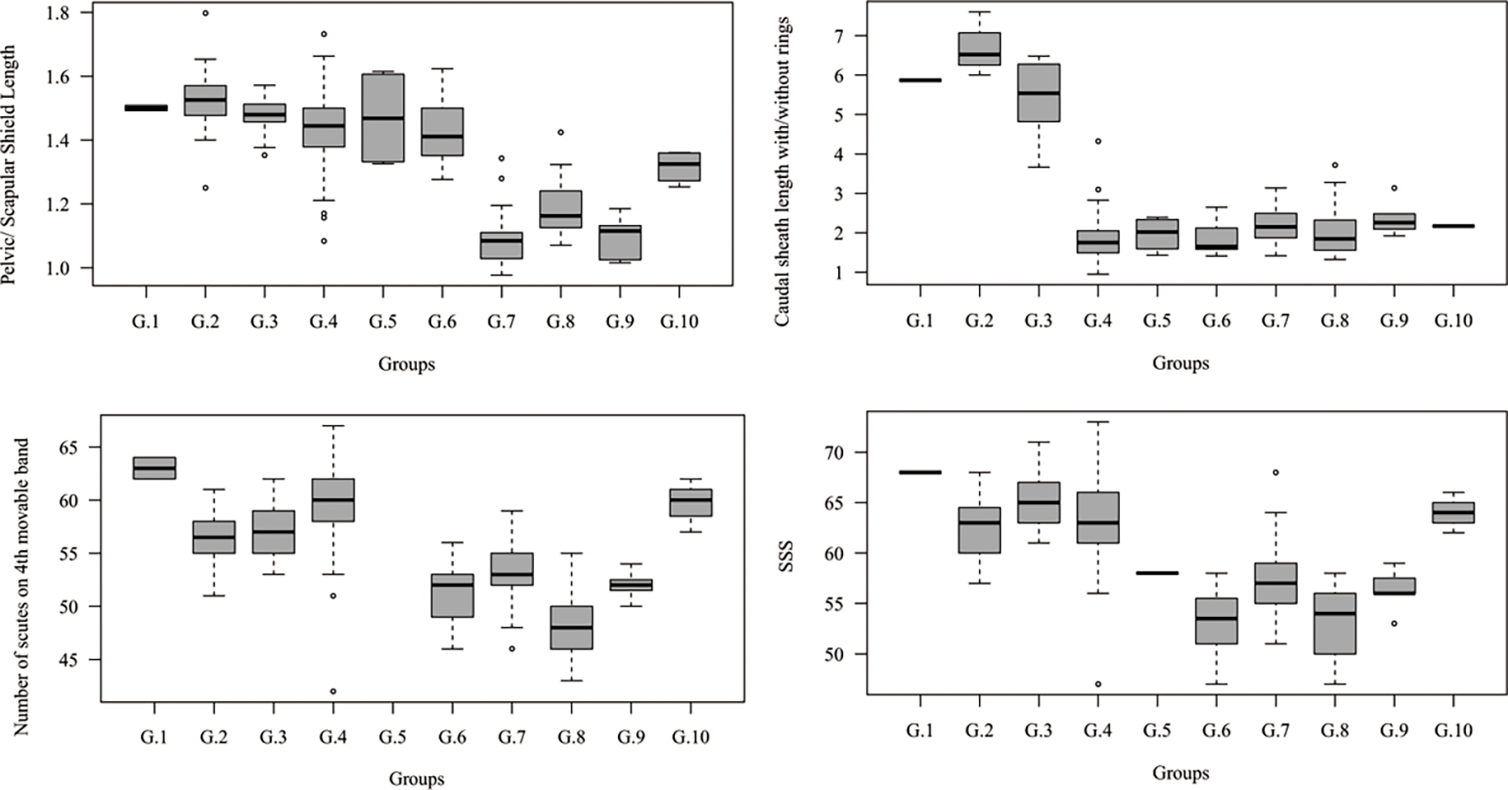
Boxplots of diagnostic carapace measurements of morphogroups of *Dasypus*. Boxes delimit the 1^st^ and 3^rd^ quantiles, the heavy midline the median, and whiskers correspond to respective quantiles -/+ 1.5 IQR. Outliers are represented by circles.

Among the carapace measurements, the size related measures show an expected pattern, similar to the PC1 in Fig 5. However, the ratio of the pelvic and scapular shield dorsal length shows a different organization (Fig 7). Groups 7, 8, and 9 exhibit the lowest values, close to one, indicating that the two shields are comparable in length, whereas the other groups show mean values around 1.5, indicating that the pelvic shield is longer than the scapular shield. In this same sense, the ratio of the caudal sheath length with/without rings clusters together specimens of Groups 1, 2, and 3, and clearly separated them from all others, indicating that the proportion of the ringed tail is larger in these groups (Fig 7). The number of scutes on the 4^th^ movable band and on the posterior border of the scapular shield (SSS) differentiated Group 1 from Groups 2 and 3 among the “large” *Dasypus*, and also differentiated Group 10 from the other “small” *Dasypus* (Fig 7).

### Taxonomic implications

The species is the most fundamental taxonomic unit and may be briefly defined as a separately evolving metapopulation lineage [71]. However, the criteria used to recognize species vary depending on the species concept adopted [71,72]. A vast and rich literature about the various definitions of species is available elsewhere (e.g. [71,73,74,75,76,77,78,79,80], and references therein). Because “species” is a conventionally fixed delimitation of an ongoing dynamic evolutionary process, every concept is a viewpoint of when a group of populations (constituting an evolving lineage) should be recognized with a binomial name. Hence, the definitions are not mutually exclusive.

Regardless of the species concept, complementary lines of evidence are desired in order to strengthen any taxonomic/systematic hypothesis. When results based on distinct approaches and systems converge in explaining a biological process, ideally reflecting a coherent biogeographic pattern, the hypothesis becomes harder to refute. In this sense, we based our conclusions on three complementary data sets (discrete characters of the skull and body, carapace measurements, size and shape of skull) that cover a variety of morphological structures of a large, geographically extensive sample of individual, local, and geographic variation. In addition, we also took into account the available molecular evidence.

Specimens of Groups 1, 2 and 3 are the largest *Dasypus* and share unique characters for the genus. They are the only ones to show an angular emargination of the anterior border of the scapular shield, enlarged projecting scales at the knee, absence of a well-defined occipital lobe on the cephalic shield, and a non-rounded lateral border of the palatine. Unsurprisingly, these three groups exhibited some morphometric overlap, although they were recovered with strong support in the cross-validation tests both via linear and geometric approaches and were differentiated based on Procrustes distances (Table 3). In addition, the result of the PCA on the shape of the skull in dorsal view shows a clear separation of these three groups (Fig 6A). Complementing these differences, each of these groups has exclusive external and cranial qualitative characters (see “Species Account”) and mirrors a well-known Amazonian biogeographic pattern. Therefore, in accordance to Feijó and Cordeiro-Estrela [3], we recognize them as three species of greater long-nosed armadillos. All three have available names that were assigned to our morphogroups based on agreement with their original descriptions, type specimen traits, and type localities. For Group 1, the oldest name available is *Dasypus beniensis* Lönnberg, 1942, for Group 2 is *Dasypus pastasae* (Thomas, 1901), and for Group 3 is *Dasypus kappleri* Krauss, 1862.

Group 4 exhibits high variability of the characters scored. Nevertheless, the specimens share unique characters (see Species Account) and are strongly differentiated from the other nine groups in the linear and geometric analyses. Therefore, we recognize it as a distinct species, for which the oldest available name is *Dasypus novemcinctus* Linnaeus, 1758. The intrapopulational variation in the nine-banded armadillo is remarkable, which has led to the description of numerous related taxa, currently regarded as synonyms of *D*. *novemcinctus* [29,33,81]. However, considering the external and cranial characters evaluated, we do not find consistent morphologic patterns within this species that justify any split. A similar conclusion was reported by Alston [29], who recognized a single species of nine-banded armadillo from Central and South America. Lönnberg [33] also made detailed comparisons of the putative taxa of nine-banded armadillo that were then recognized, stating that their presumed diagnostic traits all constitute intraspecific variation.

Group 5 exhibits the most striking traits, and in all analyses this group was clearly differentiated from all other groups. The oldest name available for these armadillos is *Dasypus pilosus* (Fitzinger, 1856).

Groups 6 and 10 are the only small-sized specimens that have 7 to 9 movable bands and a sigmoid dorsal profile of the skull. They are also very similar morphometrically, as evidenced by the linear and geometric cross-validation analyses (Tables 2 and 4). In any case, their widely disjunct geographic distributions (Fig 4) preclude any possible genetic flow between them; some diagnostic traits (see Species Account) permit each group to be recognized as a distinct species. The senior name for specimens of Group 6 is *Dasypus sabanicola* Mondolfi, 1968, and for the armadillos of Group 10 is *Dasypus mazzai* Yepes, 1933.

Groups 7, 8 and 9 comprise small-sized armadillos with 6–7 movable bands and a straight dorsal profile of the skull. Unlike *D*. *sabanicola* and *D*. *mazzai*, Groups 7 and 8 have neighboring distribution limits, whereas Group 9 represents an isolated population (around 550 km from its nearest Group 7 neighbours in Cordoba, central Argentina) (Fig 4). The external and cranial qualitative characters we employed were ambiguous in assigning specimens to each of these three groups. In the morphometric analyses, Groups 7, 8, and 9 show great similarities, overlapping to greater or lesser degrees (Figs 5, 6 and 7; Tables 2, 3 and 4; see Species Account). In spite of their resemblances, each of these three groups has a singular identity (see Species Account) and has a unique geographic distribution. O'Brien and Mayr [82] proposed the following set of requirements for the recognition of subspecies: they must have a unique geographic range, represent a group with concordant phenotypic traits, and have a unique natural history relative to other subdivisions of the species. In addition, subspecies should “intergrade almost unnoticeably … [where] there is distributional continuity” ([74]; p. 106) and so are not “clear-cut units which can easily be separated from one another” ([74]; p. 106). Therefore, we choose to recognize each of the three groups as part of a single polytypic species, whose oldest available name is *Dasypus septemcinctus* Linnaeus, 1758. Thus, based on the principle of priority, specimens of the Group 7 should be called *Dasypus septemcinctus hybridus* (Desmarest, 1804), Group 8 represents the nominate subspecies *D*. *septemcinctus septemcinctus* Linnaeus, 1758, and Group 9 constitutes a new subspecies described in the Species Account.

In summary, we recognize eight living species of *Dasypus* and three subspecies of *D*. *septemcinctus*. Detailed information about each of these taxa is provided in the Species Account section.

### Species account

**Fig 8:**
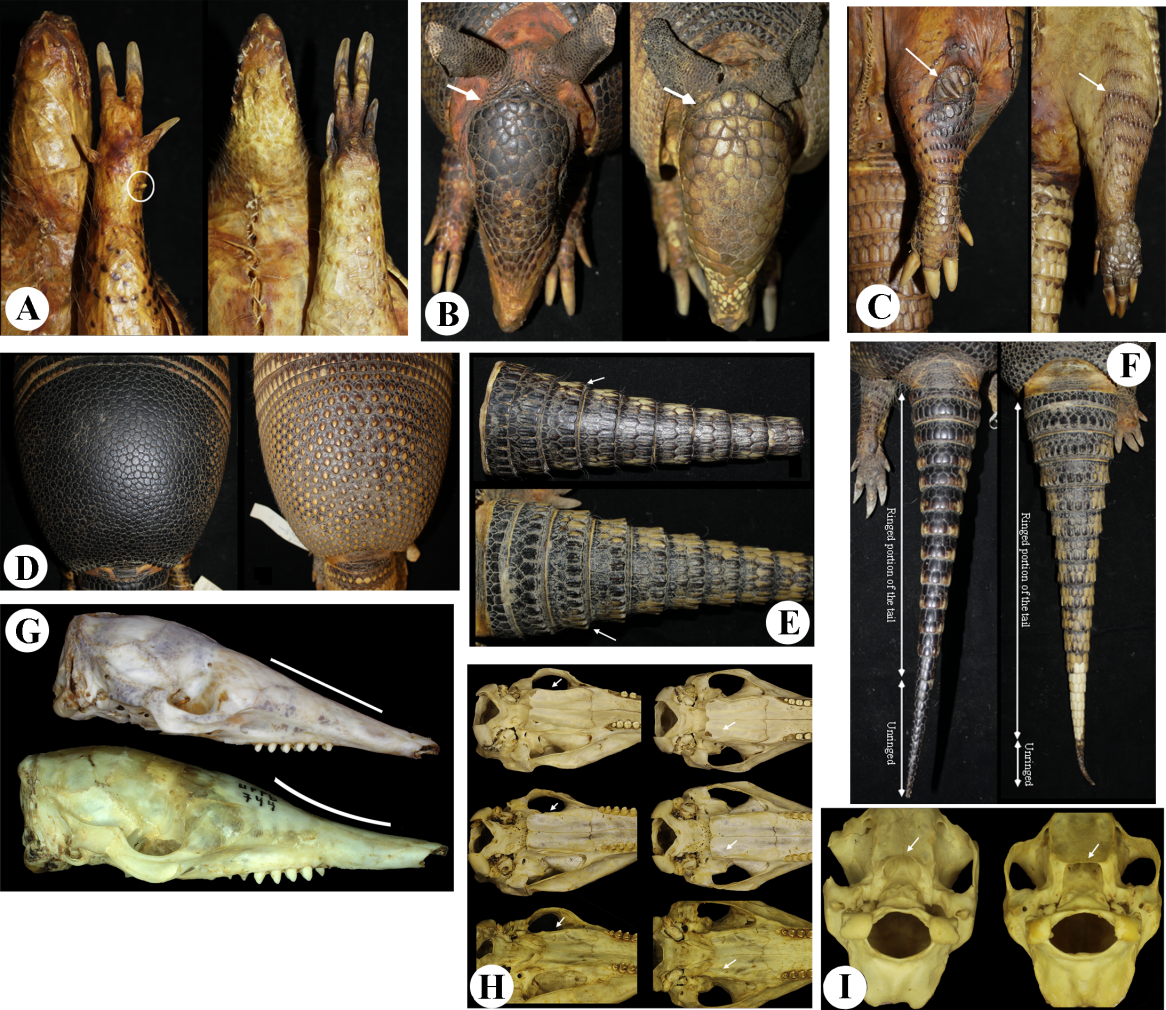
Diagnostic traits in the species of the genus *Dasypus*. **See Table 7.** A: Five (left) and four (right) digits in the forefoot; B: Poorly developed (left) and well-developed occipital lobe on cephalic shield; C: Well-developed (left) and poorly developed (right) scales at the knee; D: Smooth (left) and rough (right) scales in the pelvic shield; E: Flattened (up) and keeled (bottom) scales in the caudal sheath; F: Ring and unriged portion of the tail; G: Straight (upper) and sigmoid (bottom) dorsal profile of the skull; H: Erected and swollen (upper), erected and thin (middle) and smoothly inclined (bottom) lateral margin of the palatine; I: convex (left) and straight (right) posterior border of the palatine.

In the following section we provide general external and cranial descriptions of *Dasypus*, compare it to other Cingulata groups, and offer a brief taxonomic history of the genus. Then, we provide an account for each species of *Dasypus*, including its synonyms, type material, type locality, diagnosis, geographic distribution, taxonomic history, and remarks. External and cranial measurements of the species of *Dasypus* are in Tables 5 and 6 and were obtained from the specimens examined in this study. The list of specimens examined and list of localities are provided in the S1 Appendix. Table 7 and Fig 8 summarize the main diagnostic traits between the species of *Dasypus*. Synonymies were largely based on Cabrera [35] and Wetzel et al. [1].

**Table 5:** External measurements (in millimeters) and weight (in grams) for the *Dasypus* species.

Variables	*D*. *beniensis*	*D*. *pastasae*	*D*. *kappleri*	*D*. *novemcinctus*	*D*. *pilosus*	*D*. *sabanicola*	*D*. *septemcinctus*	*D*. *mazzai*
**TL**	932.5±31.8	971.9 ± 66.4	958.6±128.4	775.7±65.8	660.2±65.4	505.4±29.3	453.4±32.8	-
	910-955(2)	854–1052 (9)	801-1300(14)	600-1080(172)	593-750(4)	452-549(22)	380-525(46)	
**HB**	557.5±3.5	537.4±35	578.8±118.6	431.9±56.3	380.7±44.3	294.4±12.8	292.11±35.3	-
	555-560(2)	481-580(9)	496-900(14)	320-802(172)	333-440(4)	269-314(22)	244-475(44)	
**TA**	375±35.4	434.4±43.7	379.9±38	348.3±37.9	279.5±21.9	211±25.7	168.7±17.3	-
	350-400(2)	370-483(9)	305-430(14)	285-450(171)	260-310(4)	173-260(22)	128-205(43)	
**HF**	127.5±3.5	113.7±22.5	104±20	89±13.6	74±4.5	58.8± 9.7	57.3±9.9	-
	125-130(2)	63-135(8)	60-125(14)	33-120(220)	70-80(4)	37-71(20)	30-72(42)	
**E**	75(1)	53.4±7	48.6±3.5	42.12±4	44±4	27.8±3	25.9±3.6	39(1)
		45-61(7)	40.7-53(12)	30.5-56(198)	41-50(4)	24-37(21)	20-35(42)	
**W**	12000(1)	8274±1473	9032±2888	4208.7±996.4	1856.6±460	1634.3±183	1467.5±465	-
		5900-9800(5)	4000-15000(13)	1600-6550(203)	1400-2320(3)	1426-1950(6)	670-2500(23)	

All measurements are mean ± standard deviation/ minimum-maximum (N).

TL: total length; HB: head-body length, TA: tail length, HF: hind foot length, E: ear length, W: weight.

**Table 6:** Cranial measurements (in millimeters) for the *Dasypus* species.

Variables	*D*. *beniensis*	*D*. *pastasae*	*D*. *kappleri*	*D*. *novemcinctus*	*D*. *pilosus*	*D*. *sabanicola*	*D*. *septemcintus*	*D*.*mazzai*
**GLS**	133.6±3.5	122.7±5.9	128.1±5	97±5.2	107.6±4	71.9±2.1	68.9±4.8	73.3±1.2
	129.3–141.3(8)	108.9–133.2(31)	111.1–135.2(33)	78.7–111.7(702)	101–111.2(5)	67.3–75.9(23)	57.7–81.1(81)	72–74.2(3)
**CB**	120.2±3.1	112±5.3	116±4.7	89±5	98.8±4.2	65.1±1.7	63.5±4.4	66.8±1.3
	115.6–126.6(8)	99.1–119.9(31)	100.3–123.9(33)	71.6–104.6(702)	92.5–103.1(5)	61.7–68.1(23)	52.2–72.5(81)	65.5-68(3)
**APL**	25.8±0.6	24.6±2.4	25.8±2.1	21.8±1.8	31.2±2.1	15.1±0.7	15.3±1.6	14.9±0.7
	24.8–26.9(8)	20.5–31.9(31)	20–31.8(33)	14.4–27.9(702)	28.8–33.7(5)	13.5–16.1(23)	11.8–18.7(81)	14.3–15.6(3)
**PL**	89.5±2.9	85.1±4.6	87.8±4	64.1±4	74±4.3	45.7±1.9	43.6±4.3	46.2±1.2
	85.4–94.3(8)	76–91.5(31)	75.8–95.5(33)	50.6–77.5(702)	68.9–80.3(5)	41.1–49.1(23)	27.8–52.6(81)	45.5–47.6(3)
**ML**	53±2	49.3±3.6	53.3±4.4	37.5±3	48.5±2.2	26.7±1.6	24.9±2.5	26.6±0.8
	49.5–55.1(8)	41.1–55.4(31)	45.8–67.8(33)	26.7–51.2(702)	46.4–51.5(5)	23.4-29(23)	19.4–30.7(81)	25.8–27.4(3)
**PIL**	26.3±1.6	25.7±2.8	25.6±3.2	14.4±1.2	15.7±2.2	13.2±1.3	13.5±1.9	14.3±1.4
	24.4-29(8)	19.8–30.6(31)	13.5–31.3(33)	8.1–23.4(702)	13.2–19.1(5)	10.9–15.6(23)	7.9–17.5(81)	13.1–15.9(3)
**ICL**	11.6±1.3	7.8±1.5	7.4±1	7.5±1.4	6.2±0.4	5.8±0.9	4.7±1	6±1
	9.1-13(8)	5.2–11.2(31)	5.6–9.3(33)	1.7–12.3(702)	5.7–6.6(5)	4.4–7.6(23)	2.7–7.2(81)	5–7.1(3)
**MT**	30.1±1.5	29.8±2.1	29.5±1.8	23.3±1.8	19.1±0.8	17.4±0.9	15.9±1.4	17.7±0.8
	27.5–31.9(8)	23.8–33.2(31)	24.9–33.5(33)	16.8–29.1(702)	18.4–20.3(5)	15.7–19.2(23)	12.6–19.5(81)	16.8–18.4(3)
**NL**	47.4±2.5	43.4±3.1	42.9±2.6	32.2±3.1	42.4±2.9	22.8±1.3	21.2±2.1	22.2±1.5
	44.4–51.4(8)	37.3–50.2(31)	34.9–46.9(33)	13.8–42.7(702)	39–46.1(5)	20.5–25.1(23)	16–25.5(81)	20.6–23.4(3)
**LL**	19.5±1.6	14±2.1	15.2±2.3	10.5±1.4	11.6±1.9	7.4±0.8	5.8±1	7.7±0.6
	17.4–21.2(8)	9.9–17.9(31)	8.7–19.1(33)	5.1–14.7(702)	8.4–13.3(5)	19.8-23(23)	3.3–11.2(81)	7.2–8.4(3)
**RL**	87±3	78.7±4.8	81.6±3.9	58.6±4.1	72.3±4.1	41±1.8	38.8±3.2	41.3±0.6
	83–92.9(8)	67.5–86.6(31)	69–88.4(33)	39–70.9(702)	65.7-76(5)	37.2–43.8(23)	31.2–45.6(81)	40.6–41.6(3)
**AB**	43.2±1.4	37.1±2	39.5±2	32.5±2.1	27.4±1.7	24.2±1.3	22.6±1.6	25.7±0.5
	41.4–45.5(8)	33.7–40.9(31)	32.3–42.2(33)	23.4–39.6(702)	25.2–29.5(5)	21.9–26.2(23)	18.5–26.4(81)	25.2–26.1(3)
**TL**	3.2±0.3	2.6±0.2	2.8±0.3	2.1±0.3	1.1±0.1	1.6±0.2	1.4±0.2	1.6±0.2
	2.7–3.5(8)	2.1–2.9(31)	2.1–3.4(33)	1.2–3.3(702)	1–1.2(5)	1.3–1.9(23)	0.7-2(81)	1.5–1.8(3)
**PB**	20.7±0.8	19.3±1.2	19.3±2.2	14.6±2	11.7±0.7	12.1±0.7	10.5±1.6	11.3±0.5
	19.5–21.9(8)	16.7–21.4(31)	11.3–22.1(33)	8.2–19.8(702)	10.5–12.1(5)	10.5–13.7(23)	6.4–13.8(81)	10.8–11.6(3)
**PIB**	19.5±0.7	19.5±1.2	21.1±1.1	18±2.2	11.5±0.9	11.8±0.8	11.2±1	11.8±0.5
	18.5–20.7(8)	17.4–21.7(31)	18.2–23.4(33)	7.1–25.4(702)	10.2–12.3(5)	9.8-13(23)	8.2–14.1(81)	11.3–12.2(3)
**PC**	30.2±1.2	25.6±1.1	27.4±1.2	23.2±1.4	20.3±0.8	18.7±1	17.4±1	19.6±0.6
	27.9–31.7(8)	23–27.6(31)	23.9–30.1(33)	18.6–34.9(702)	19.3–21.2(5)	16.4–20.6(23)	15.1–20.2(81)	19–20.1(3)
**BB**	38.4±0.7	36.1±1.5	38±1.7	31.1±1.5	27.7±1.2	26.1±1	24.7±1.3	26.6±0.326.2–26.8(3)
	37.2–39.2(8)	33.1–39.1(31)	33.6–41.6(33)	19.3–41.5(702)	26.1–29.1(5)	24.2–27.8(23)	21.3–27.3(81)	21.3–27.3(81)
**ZB**	54.4±1.9	47.9±2.9	50.7±2.7	40.8±2.6	35±1.7	31.8±1.4	29.6±2.3	31.4±1.3
	51.7-58(8)	41.2–53.8(31)	41.9–54.8(33)	26.6–49.1(702)	32.2–36.6(5)	29.2–34.2(23)	24-34(81)	30.1–32.6(3)
**MB**	34.7±0.7	33.1±1.4	34±1.4	27.5±1.5	25.5±0.8	21.8±0.8	21.5±1.4	23±0.5
	33.3–35.6(8)	29.8–36.3(31)	30.1–37.2(33)	20.6–36.7(702)	24.5–26.3(5)	19.8-23(23)	18.5–24.9(81)	22.5–23.4(3)
**HJ**	9.7±1	9.2±1	9±1.2	6.7±1	6±0.8	5.3±0.7	4.3±0.8	5.4±0.1
	8.8–11.5(8)	7.1–11.3(31)	5.9–12.2(33)	4.2–10.5(702)	4.7–6.6(5)	4.2–6.9(23)	2.4–5.9(81)	5.3–5.5(3)
**MAL**	104.9±3	97.1±4.9	101.5±4.4	76.7±4.6	87.5±3.9	55.8±1.8	53.5±4.2	56.8±0.7
	100.6–111.1(8)	83.3–104.1(31)	86.9–111.4(33)	57-5-89.8(702)	81–90.7(5)	51.3-59(23)	40.7–61.2(81)	56.1–57.5(3)
**LMT**	32.4±1.1(8)	31.2±2.2	31.6±1.7	24.9±1.8	20.9±3	18.6±1.1	17.2±1.4	19.5±0.6
	31.1–34.2	25.2–35.1(31)	27.6-35(33)	17.5–30.4(702)	15.8–23.5(5)	16.3–20.6(23)	12.8–20.9(81)	7.2–8.4(3)
**AML**	23.7±0.5	22.2±2.1	23.7±2.3	19.9±1.9	28.2±1.8	13.9±0.9	14.2±1.8	14.4±0.6
	22.9–24.4(8)	23.4–32.9(31)	17.9–29.3(33)	11.7–28.2(702)	25.5–29.9(5)	14.8–15.4(23)	8.9–17.6(81)	13.8-15(3)
**HM**	26.4±2.4	27.8±2.1	29.4±2	22.2±2	20.5±1.7	16.7±1.2	16.9±1.9	16.8±1.1
	23.5–30.5(8)	23.4–32.9(31)	25.1–32.6(33)	16.9–28.5(702)	17.6–21.9(5)	14.3+18.7(23)	11.5–22.6(81)	15.9–18.1(3)

All measurements are mean ± standard deviation/ minimum-maximum (N).

GLS: Greatest length of skull, CB: Condylobasal length, APL: Anterior palatal length, PL: Palatal length, ML: Maxilla length, PIL: Palatine Length, ICL: Infraorbital Canal length, MT: Maxillary toothrow length, NL: Nasal length, LL: Lacrimal length, RL: Rostral length, AB: Anteorbital breadth, TL: Tooth length, PB: Palatal breadth, PIB: Palatine breadth, PC: Postorbital constriction, BB: Braincase breadth, ZB: Zygomatic breadth, MB: Mastoid breadth, HJ: Height of jugal bone, MAL: Mandible length, LMT: Mandibular toothrow length, AML: Anterior mandibular length, HM: Height of mandible.

**Table 7:** Comparison of diagnostic traits in the species of the genus *Dasypus* (see Fig 8).

Species		Traits												
	Size	Digits on forefoot (Fig 8A)	Occipital lobe on cephalic shield (Fig 8B)	Scales at the knee (Fig 8C)	Scales in pelvic shield (Fig 8D)	Scales in caudal sheath (Fig 8E)	Relative length of ringed portion of tail (Fig 8F)	Scutes on posterior border of scapular shield	Movable bands	Scutes on the 4th movable band	Hairy carapace	Dorsal profile of skull (Fig 8G)	Lateral margin of palatine (Fig 8H)	Posterior border of palatine (Fig 8I)
***D*. *beniensis***	Large (932 mm)	5	Poorly developed	Well developed	Rough	Flattened	85%	68	7–8	62–64	Absent	Sigmoid	Smoothly inclined	Convex
***D*. *pastasae***	Large	5	Poorly developed	Well developed	Rough	Flattened	85%–88%	57–68	7–8	51–61	Absent	Sigmoid	Erect and thin	Straight
	(972 mm)													
***D*. *kappleri***	Large	5	Poorly developed	Well developed	Smooth	Keeled	78%–86%	61–71	7–8	53–62	Absent	Sigmoid	Erect and swollen	Straight
	(958 mm)													
***D*. *novemcinctus***	Medium	4	Well developed	Poorly developed	Smooth	Flattened	52%–75%	56–73	8–10	51–67	Absent	Sigmoid	Rounded	Convex
	(775 mm)													
***D*. *pilosus***	Medium	4	Well developed	Poorly developed	Smooth	Flattened	59%-70%	~58	9–11	-	Present	Sigmoid	Rounded	Convex
	(660 mm)													
***D*. *sabanicola***	Small	4	Well developed	Poorly developed	Smooth	Flattened	58%–72%	47–58	7–9	46–56	Absent	Sigmoid	Rounded	Convex
	(505 mm)													
***D*.*septemcinctus***	Small	4	Well developed	Poorly developed	Smooth	Flattened	56%–76%	47–68	6–7	43–59	Absent	Straight	Rounded	Convex
	(457 mm)													
***D*. *mazzai***	Small	4	Well developed	Poorly developed	Smooth	Flattened	-	62–66	8–9	57–62	Absent	Sigmoid	Rounded	Convex

Numbers in parenthesis are the mean of total length of the body.

***Dasypus* Linnaeus**, **1758**

*Dasypus* Linnaeus, 1758: 50; type species *Dasypus novemcinctus* Linnaeus, 1758 by Linnean tautonomy.

*Tatus* Fermin, 1769: 110; unavailable name due to the non-binominal work [83].

*Tatu* Frisch, 1775: Table; unavailable name due to the non-binominal work [84].

*Tatu* Blumenbach, 1779: 74; type species *Tatu novemcinctus* by monotypy.

*Tatus* Olfers, 1818: 220 part; incorrect subsequent spelling of *Tatu* Blumenbach

*Cataphractus* Storr, 1780: 40 part; no species mentioned; name included all then-known armadillos.

*Loricatus* Desmarest, 1804: 28; type species *Loricatus niger* Desmarest, 1804 designated by Wetzel et al. [1].

*Tatusia* Lesson, 1827: 309; part; type species *Dasypus peba* Desmarest, 1822 designated by Wetzel et al. [1].

*Cachicamus* McMurtrie, 1831: 163; type species *Dasypus novemcinctus* Linnaeus, 1758, designated by Wetzel et al. [1].

*Cachicama* P. Gervais in I. Geoffroy St. Hilaire, 1835: 53; invalid emendation of *Cachicamus* McMurtrie, 1831.

*Zonoplites* Gloger, 1841: 114; no species mentioned, name proposed for armadillos with four toes on forefeet, the two middle toes being longer than outer toes.

*Praopus* Burmeister, 1854: 295; type species *Dasypus longicaudus* Wied-Neuwied, 1826 by monotypy.

*Cryptophractus* Fitzinger, 1856: 123; type species *Cryptophractus pilosus* Fitzinger, 1856 by monotypy.

*Hyperoambon* Peters, 1864: 180; type species *Dasypus pentadactylus* Peters, 1864 designated by Wetzel and Mondolfi ([36]; p. 56).

*Muletia* Gray, 1874: 244; type species *Dasypus septemcinctus* Gray, 1874 by monotypy (= *Loricatus hybridus* Desmarest, 1804; not *Dasypus septemcinctus* Linnaeus, 1758).

*Tatua* Robinson and Lyon, 1901: 161; incorrect subsequent spelling of *Tatu* Blumenbach.

*Mulletia* Yepes, 1928: 506; incorrect subsequent spelling of *Muletia* Gray.

*Mulietia* Talmage and Buchanan, 1954: 80; incorrect subsequent spelling of *Muletia* Gray.

**Type species:** *Dasypus novemcinctus* Linnaeus, 1758 by Linnaean tautonomy.

**Content**: Eight living species (*D*. *beniensis*, *D*. *pastasae*, *D*. *kappleri*, *D*. *novemcinctus*, *D*. *pilosus*, *D*. *sabanicola*, *D*. *mazzai*, and *D*. *septemcinctus*) plus two extinct species (*D*. *bellus* and *D*. *punctatus*, see Castro [85]).

**Description:** *Dasypus* comprises small to large armadillos (Total length: 380–1300 mm; weight: 1–15 kg). The head is conical, with a long and tubular rostrum. The ears have a conical shape and are located dorsally, with little spacing between them and the ear canal is directed laterally. A cephalic shield is closely attached to the skull and covers almost the entire dorsal surface of the head, except for the anteriormost portion. The body has both the dorsum and lateral aspects completely covered by a bony carapace composed of scutes. The carapace is tripartite, comprised of scapular and pelvic shields, separated by transverse, overlapping movable bands. The length of pelvic shield is as big or bigger than the scapular shield. Movable bands are transverse rows laterally linked by overlapping scutes. Bands are separated by soft tissue, allowing the animal to bend the body. In *Dasypus*, the number of bands range from six to eleven. Externally, the movable bands exhibit two juxtaposed triangular types of scutes, one pointed anteriorly with a large base, the other shorter and directed posteriorly. The ventral region of the body is almost bare, but has subtle, small transverse rows of osteoderms, from which long whitish hairs emerge. The tail is totally covered by osteoderms forming the caudal sheath. The osteoderms are arranged in nine to 19 concentric rings that collectively cover 50% to 89% of the tail. Rings are separated from each other by a tiny band of tissue, making the tail flexible. Each ring is formed by two to three rows of juxtaposed transversely arranged osteoderms, the posteriormost row exhibiting larger and more rectangular scutes. On the distal half of the tail, there are two to four shallow sagittal sulci. The hindlimbs are longer and more robust than the forelimbs. The forefeet have four or five fingers, the second and third are the largest with modest claws, and the fifth, when present, is rudimentary. The hindfeet have five toes, the middle is the largest.

The color of the carapace varies from uniform grayish or brownish-gray to having a darkened dorsum with a yellowish lateral stripe, which can cover half to two-thirds of the carapace. On this lateral stripe, scutes of the movable bands are yellowish posteriorly but brownish anteriorly, and on the scapular and pelvic shield, the central region of each scute is yellowish whereas the peripheral area is brownish. The dorsal color of the tail can be totally black, dark brown, or bicolored, with the anterior half of caudal rings brownish and their posterior yellowish. Ventrally, the tail is uniformly yellowish.

In *Dasypus*, the external appearance of scutes covered by epidermal horny scales does not completely resemble the actual shape of bone osteoderm. The osteoderms of the cephalic shield have a polygonal outline and are juxtaposed, but loosely attached on the anterior portion. At its posterior margin, there is a transverse row of mostly rectangular osteoderms -the occipital lobe- separated from the rest by a shallow sulcus, which can be either conspicuous or barely perceptible. In lateral view, small osteoderms of the head are loosely distributed in rows around the eyes. The osteoderms on the scapular and pelvic shield are hexagonal or pentagonal in shape and tightly juxtaposed, forming a rigid structure. Each osteoderm, on its external surface, has a central and peripheral area, divided by a principal sulcus. The central area can be either circular or polygonal. The peripheral area has 3 to 4 small trapezoidal sections located anterior and lateral to the central region, each divided by radial sulci. In some specimens, the sulci are indistinct or absent, leaving a uniform surface. External surface foramina in the principal sulcus are usually present, and number from three to 30. The osteoderms of the movable bands are rectangular, divided into two parts: a small flattened anterior base, attached ventrally to the previous band, and a longer posterior part, which is exposed and has a horny epidermal cover. The longer part has a triangular central main figure, divided from the peripheral region by principal sulci, which can be connected anteriorly or not. Foramina are present both in the sulci and on the posterior margin of osteoderm.

The skull is conical, with an elongate rostrum (41% to 70% of greatest skull length). Nasals are long, narrow, and extended beyond the premaxilla. The maxilla is expanded laterally on its posterior half, making a notable angle on the sides of the rostrum. Frontal is well developed and slightly vaulted. The sagittal crest is usually absent or barely noticeable. In dorsal view, the zygomatic arches are parallel to each other or slightly convergent posteriorly. The nuchal crest is present and can be well developed. The external occipital crest is large and robust. The palatine is long, extending to the level of the squamosal process of the zygomatic arch, and either flattened or concave. The maxillo-palatine suture lies at or beyond the level of the last teeth. The posterior border of the palatine is variably connected to the pterygoid, whereas the lateral border can be rounded or exhibit an erect crest. *Dasypus* exhibits tooth replacement (diphyodonty). Permanent teeth are molariforms and euhypsodont. The most anterior ones are laterally compressed and the central ones are the largest, with both lingual and labial cusps. The upper teeth are restricted to the maxillary bone. The number of teeth varies from six to nine on each quadrant of the upper and lower jaws. Specimens with bilateral asymmetries in the number of teeth on skull and mandible, as well as occlused alveoli mainly at end of toothrow, are common. The ectotympanic is ring-shaped, which partially covers the entotympanic. The petrosal is small, ovoid and robust. In lateral view, the dorsal profile of the skull is sigmoidal, but in *D*. *septemcinctus* it is straight. The middle portion of the jugal is expanded ventrally and its dorsal profile is concave. The jugal-squamosal suture is vertical or slightly oblique in orientation. The lacrimal is rounded or triangular shape. The anterior border of the infraorbital foramen is aligned with the anterior border of the lacrimal, either slightly posterior or anterior to it. The mandible is slender, slightly curved, and dorsoventrally shallow. The mandibular symphysis is weakly fused and long, extending nearly to the toothrow. From the body to the vertical ramus there is a smooth slope. The coronoid process is very long, narrow, posteriorly inclined, sharpened, and located well above of toothrow. The condylar process is very short, slightly above the toothrow, and posteriorly oriented. A deeply curved acute angle separates the coronoid and condylar processes. The angular process is abrupt and separated from the condyloid by a short, shallow curve.

**Comparisons:** *Dasypus* is easily distinguished from other genera by the dorsal position of ears; conical head and slender face; ringed arrangement of osteoderms on more than 50% of the length of the tail; and osteoderms of the carapace closely juxtaposed to each other. In addition, it differs from *Euphractus*, *Chaetophractus*, and *Zaedyus* by the absence of teeth on the premaxilla; an ectotympanic ring-shaped; a maxillo-palatine suture positioned posterior to the toothrow; teeth less robust; a slender mandible, with a posterior sloping orientation of the vertical ramus; and a condyloid process distant from the coronoid process. *Dasypus* differs from *Cabassous* by the presence of caudal sheath; modest claws; and greater height of the coronoid process relative to the condylar process. *Priodontes* is distinguished by its larger size (adults can reach more than 50 kg), higher numbers of teeth (65–98) and movable bands (11 to 13), and smaller coronoid process. *Tolypeutes* has the fewest movable bands (three) and a very short tail (50–70 mm). *Calyptophractus* and *Chlamyphorus* are the smallest Cingulata (head and body length around 150 mm), have vestigial eyes and ears, the sides and venter of the body totally covered by white hairs; and a peculiar vertical, flat and large independent portion of the carapace covering the rump.

**Brief taxonomic history:** The nomenclature of the long-nosed armadillos had a dynamic history until the beginning of the XX^th^ century. The taxonomic history of *Dasypus* began when Linnaeus ([20]; p. 50–51) described six species: *D*. *unicinctus* (= *Cabassous unicinctus*), *D*. *tricinctus* (= *Tolypeutes tricinctus*), *D*. *quadricinctus* (= *Tolypeutes tricinctus*), *D*. *sexcinctus* (= *Euphractus sexcinctus*), *D*. *septemcinctus*, and *D*. *novemcinctus*. Later, Fermin [86] and Frisch [87] used the names *Tatus* and *Tatu*, respectively, although both names are unavailable because they did not follow the principles of binominal nomenclature [1,83,84]. Blumenbach ([88]; p. 74) was the first to properly use the name *Tatu*, with [*Tatu*] *novemcinctus* (= *D*. *novemcinctus*) as its type species by monotypy. Other names available, but barely used, are *Cataphractus* Storr, 1780, *Loricatus* Desmarest, 1804, and *Cachicamus* McMutrie, 1831 (see synonymy for the complete list).

Lesson ([21]; p. 307) classified the armadillos into four genera, *Dasypus*, *Chlamyphorus*, *Priodontes* and *Tatusia*. The first included only *Dasypus encoubert* Desmarest, 1822 (= *Euphractus sexcinctus* (Linnaeus, 1758)). The name *Tatusia* was clearly based on “Tatusies” of Cuvier ([89]; p. 197) in reference to the species without teeth in the premaxilla, and included *D*. *novemcinctus*, *D*. *septemcinctus*, and *D*. *hybridus* Desmarest, among others. Soon after, Wagler [22] proposed a different arrangement, and placed Linnaeus’ *D*. *septemcinctus*, *D*. *octocinctus* (= *D*. *novemcinctus*), and *D*. *novemcinctus* into *Dasypus*, while allocating *Dasypus sexcinctus* (= *Euphractus sexcinctus*) into the new genus *Euphractus*.

Both Lesson’s classification and Blumenbach’ *Tatu* were largely followed by subsequent authors (e.g. [25, 26,27,28,29,30,31], until Thomas ([32]; p. 141), employing the principle of tautonomy, selected *Dasypus novemcinctus* as type species of *Dasypus* Linnaeus, 1758. Since then, long-nosed species of armadillos have been consistently referred to *Dasypus*.

The integrity of the genus *Dasypus* as currently constituted has long been debated. Burmeister ([23]; p. 276) divided the genus into two groups: *Dasypus*, with five species, and *Praopus*, represented only by *Dasypus longicaudus* (= *D*. *novemcinctus*). Gray [25,26] allocated the species into three genera: *Tatusia*, including *T*. *peba*, *T*. *leptorhynchus*, *T*. *mexicana*, *T*. *boliviensis*, *T*. *leptocephala*, *T*. *granadiana* and *T*. *brevirostris* (all now *D*. *novemcinctus*); *Muletia*, including *Muletia septemcincta* (= *D*. *septemcinctus* and *D*. *hybridus*); and *Praopus*, including *P*. *kappleri* (= *D*. *kappleri*). Fitzinger [90] described a new genus and species, *Cryptophractus pilosus*, and reported its similarities to *D*. *novemcinctus*. Peters [24] named a new genus, *Hyperoambon*, for his new species, *Dasypus pentadactylus* (= *D*. *kappleri*). Rhoads [91], taking into account the variability of diagnostic traits listed by Gray [26], placed *Muletia* as subgenus of *Tatusia*. Talmage and Buchanan [81] listed the Fitzinger’s hairy armadillo as a subgenus, *Dasypus (Cryptophractus) pilosus*. In the last comprehensive revision of the genus, Wetzel and Mondolfi [36] recognized three subgenera: *Hyperoambon* Peters, 1864, *Crypthophractus* Fitzinger, 1856, and *Dasypus* Linnaeus, 1758.

Recently, Rincón et al. [54] noted similarities of *D*. *kappleri* with extinct *Propraopus* and *Dasypus bellus*, and suggested that they belong to the same taxon, recognizable either as a subgenus or genus. Castro et al. [6] proposed recognizing *D*. *(Crypthophractus) pilosus* as a distinct monotypic genus, based on its exclusive traits and its external position in their cladistic morphological analysis. Curiously, in their phylogenetic tree based on morphological characters, *D*. *kappleri* showed the most recent divergence, together with the extinct *D*. *punctatus*, contrary to the view that *D*. *kappleri*, *Dasypus bellus*, and *Propraopus* share unique features that unite them at the generic or subgeneric level [54]. Subsequently, Gibb et al. [10] published a phylogenetic tree of xenarthran species based on mitogenomes. In their phylogeny, *D*. *kappleri* (sensu lato) is the sister taxon of all other *Dasypus* species, while *D*. *pilosus* is the last lineage to diverge, contrary to the results of Castro et al. [6].

The genus is the most important higher category [92] and should include monophyletic sets of species that share common traits. The debate, however, starts in deciding whether one or few species are different enough to be recognized as a distinct genus. According to Mayr [74], “the degree of differences between two species is not necessarily a generic criterion, because species are more distinct to the taxonomist in some families than genera are in other families.” In similar fashion, Garbino [92] suggested multiple sources of evidence are needed to properly substantiate generic rank. In addition, stability is also an important feature related to the genera, as they are the linchpins of binominal nomenclature.

A current major challenge of taxonomic classification is combining the nuanced structure of phylogenies with the practical limitations of a Linnean hierarchical system. Because *Dasypus* is the only living representative of an ancient lineage of armadillos, and its species are not only morphological and molecular relatives but also share a unique reproductive system and ecological traits, we here consider *Dasypus* as a polytypic genus, with eight extant species. We do not follow the subgeneric classification used by Wetzel and Mondolfi [36], as those groups are not monophyletic according to available phylogenetic studies [6,10].

***Dasypus beniensis* Lönnberg, 1942**

Figs 9 and 10

**Fig 9:**
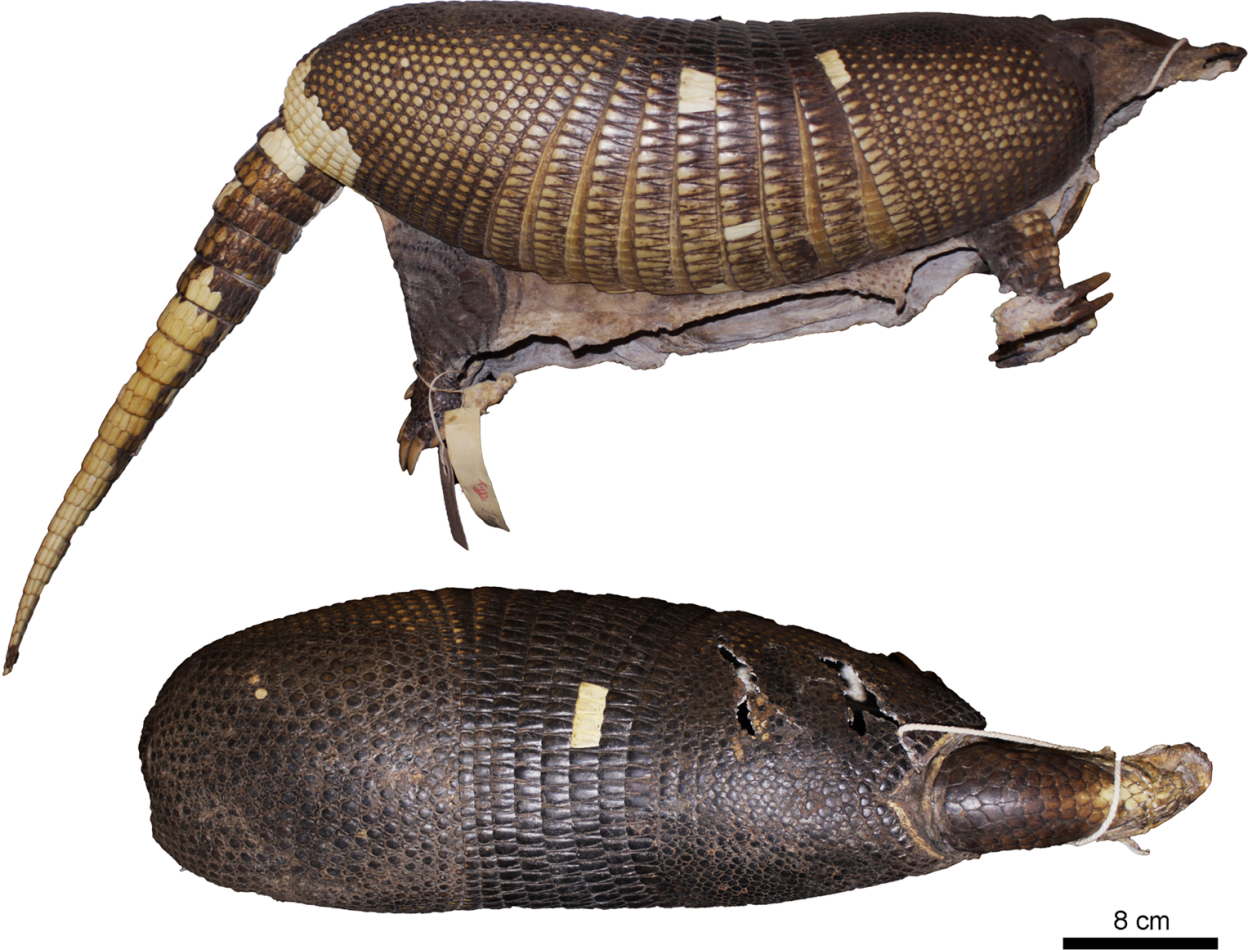
Lateral and dorsal view of the carapace, tail, and limbs of the holotype of *Dasypus beniensis* (NRM 583386).

**Fig 10:**
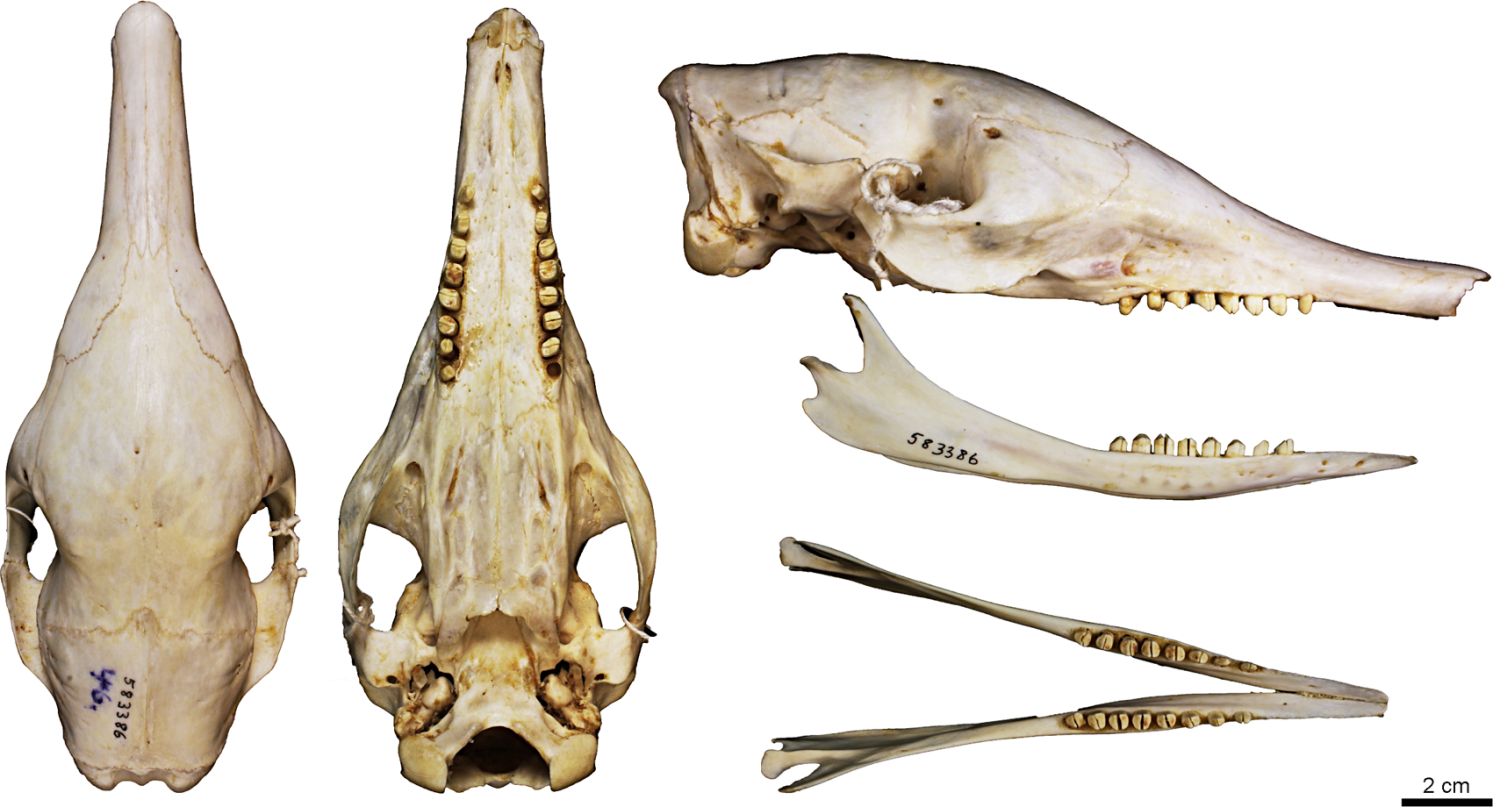
Skull and mandible of the holotype of *Dasypus beniensis* (NRM 583386).

*Dasypus kappleri beniensis* Lönnberg, 1942:49; original description.

**Type:** Lönnberg [51] mentioned an adult female specimen collected on 25 October 1937 by A. M. Olalla, but no collection number was cited. The holotype by monotypy is registered at Swedish Museum of Natural History, Stockholm as NRM 583386. It consists of an open carapace with the tail, limbs and cephalic shield attached, and separated skull and mandible (Figs 9 and 10).

**Type locality:** “[N]ear the confluence of Rio Madre de Dios with Rio Beni, Victoria, Bolivia” ([51]; p. 49). Anderson ([93]; p.118) stated that the type locality is “3 km from the left bank of río Beni and about 9 km from confluence with río Madre de Dios”, Victoria, Pando, Bolivia.

**Diagnosis:** *D*. *beniensis* is easily distinguished by a forefoot with an externally visible fifth digit, by the absence of a well-defined occipital lobe on the cephalic shield, an angular emargination of the anterior margin of the scapular shield, enlarged projecting scales at the knee, rough scales on the pelvic shield, and flattened scales in the proximal rings of the tail, a smoothly inclined lateral palatine crest, a convex posterior margin of the palatine, a well-developed and smoothly curved lacrimal bone, and a pentagonal, weakly developed tentorial process of the parietals (see Table 7).

**Fig 11:**
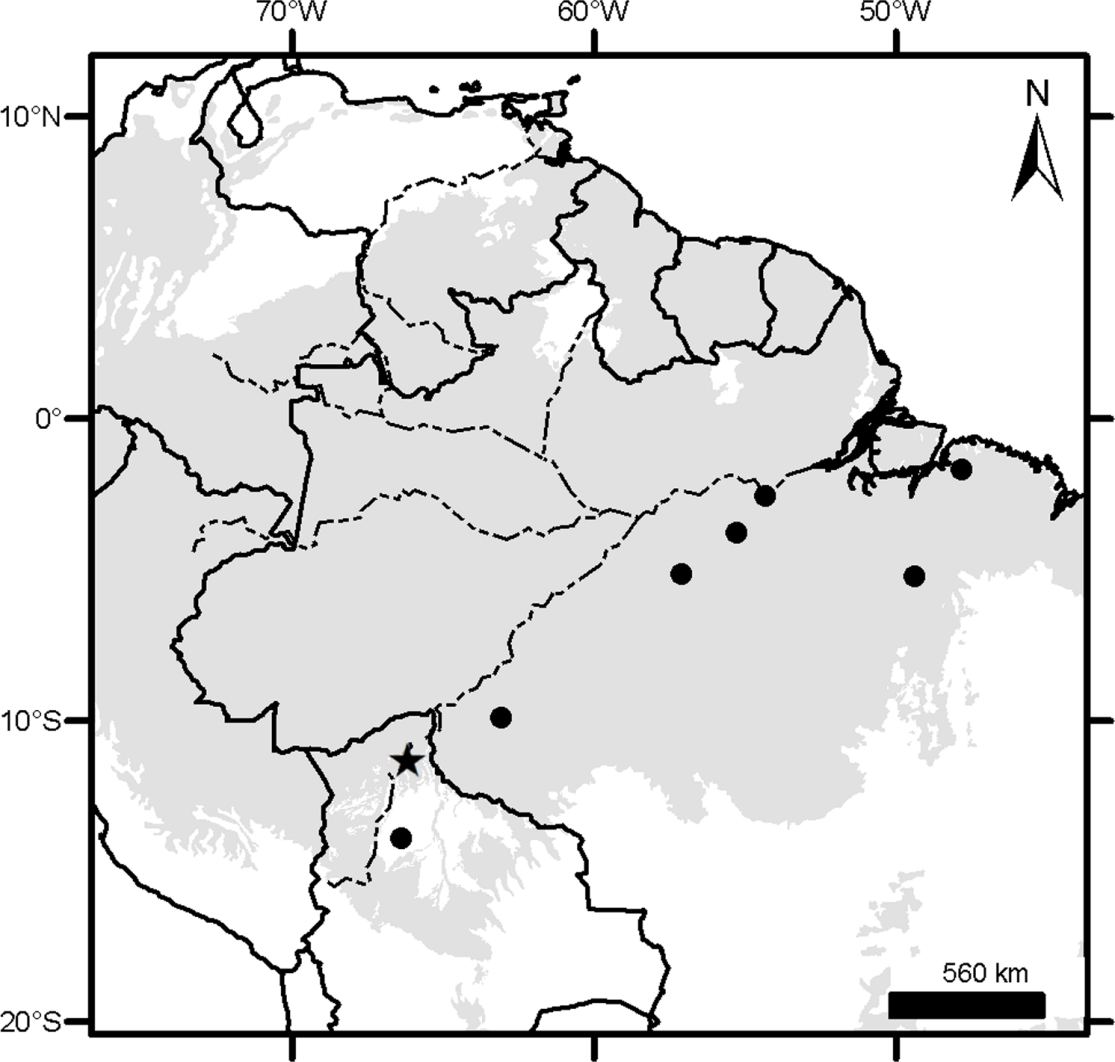
Localities recorded for *Dasypus beniensis*. Gray shading represents the moist broadleaf forest from central and northwestern of South America and dotted lines are the main rivers. Star represents the type locality.

**Distribution:** *Dasypus beniensis* is known from the right bank of the lower Amazon and Madeira rivers in Brazil and the right bank of the Madre de Dios River in Bolivia (Fig 11). These three major rivers appear to represent northern geographic limits for this species; the southern barrier seems to be dry forests and savannas in Bolivia (Chaco) and Brazil (Caatinga and Cerrado).

**Taxonomic history:** Lönnberg [51] described a subspecies of the *kappleri*-group from Bolivia as *Dasypus kappleri beniensis*. Among the characters listed, Lönnberg [51] highlighted “the most striking differences” as being the structure of the post-dental portion of the palate, a reference to the lateral margin of the palatine. Later, without explanation, Cabrera [35] treated it as a junior synonym of *D*. *kappleri pastasae* (Thomas, 1901), which was followed without comment by all subsequent authors, including Wetzel and Mondolfi [36], Gardner [38], and Wetzel et al. [1]. Feijó and Cordeiro-Estrela [3] revised the taxonomy of the ‘*kappleri-*group’ and found that the smoothly sloping border of the palatine is an exclusive and consistent trait found in all specimens distributed on the right margins of the Madre de Dios, Madeira and lower Amazon rivers. Together with other external and cranial characters and morphometric data, they proposed the species rank for Lönnberg’s supposed subspecies.

**Remarks:** Besides the diagnostic traits listed above, Lönnberg [51] pointed out other external and cranial characters to differentiate *D*. *beniensis* from other taxa in the *kappleri-*group. Externally, he mentioned the number of scutes on the posterior border of the scapular shield, on the 3^rd^ and 4^th^ movable bands, and on the border of the pelvic shield; the first ring completely formed on the base of the tail; the more rounded shape of the base of the tail; the number of rings on the tail; and the shape of the scutes on movable bands as diagnostic characters. For the skull, Lönnberg [51] cited the ventral edges of the zygomatic arches at the level of the palate and the size of the teeth. However, all these traits exhibit high intraspecific variation, and therefore are with little taxonomic value.

Most of the specimens of *D*. *beniensis* tend to have a narrower basisphenoid-basioccipital suture in relation to *D*. *kappleri* (e.g. MPEG 12331, MPEG 4678, MPEG 4676), although some specimens depart from that pattern (e.g. MPEG 8481).

***Dasypus pastasae* (Thomas, 1901)**

Fig 12

**Fig 12:**
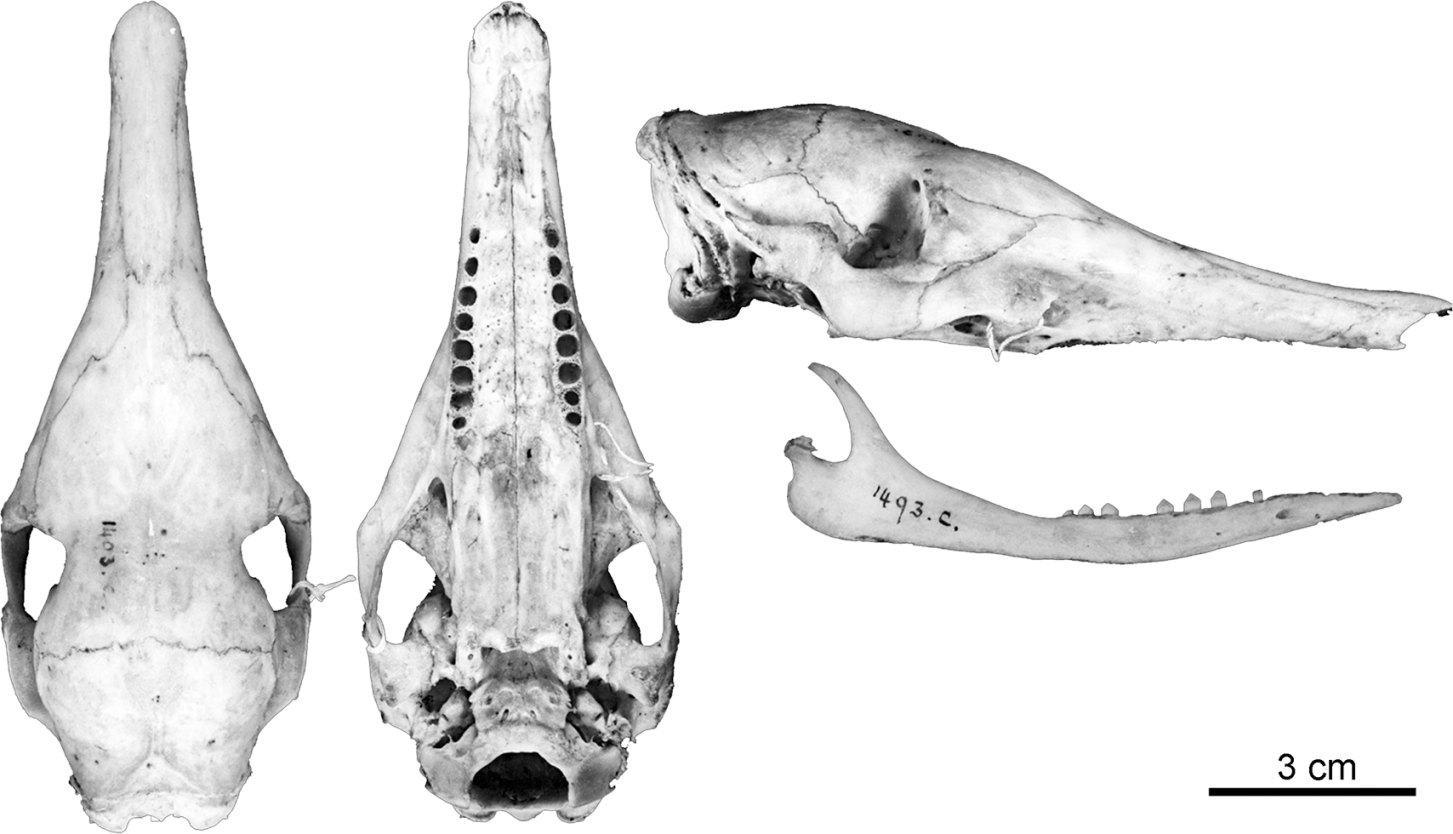
Skull of the holotype of *D*. *pastasae* (BM 80.5.6.71).

*Tatusia kappleri*: Thomas, 1880:402; part, not *Dasypus kappleri* Krauss, 1862

*Tatu pastasae* Thomas, 1901:370; original description

*Dasypus kappleri pastasae*: Lönnberg, 1928:9; name combination

*D*[*asypus*]. *k*[*appleri*]. *peruvianus* Lönnberg, 1928:10; type locality “Roque in Eastern Peru, S.E. of Moyobamba at an altitude of about 1030 m.,” San Martin, Peru.

*Dasypus pastasae*: Sanborn, 1929:258; name combination

**Type:** The holotype (BM 80.5.6.71) determined by Thomas [31] consists of the skin and skull (Fig 12) of an adult collected by Mr. Clarence Buckley.

**Type locality:** “Sarayacu, upper Pastasa River,” Pastaza, Ecuador.

**Diagnosis:** The main diagnostic external features of *D*. *pastasae* are an externally visible fifth digit on the forefoot, the absence of a well-defined occipital lobe on the cephalic shield, an angular emargination of the anterior border of the scapular shield, enlarged projecting scales at the knee, and the scales of the pelvic shield and tail rings. This species has non-uniform scales on the pelvic shield in both size and texture (roughness), where the central ones are larger and protrude beyond the smaller peripheral ones. Further, the posterior scales on the proximal rings of the tail are flattened. Cranially, the lateral margin of palatine has a prominent but uninflated crest and the posterior end of palatine is straight.

**Fig 13:**
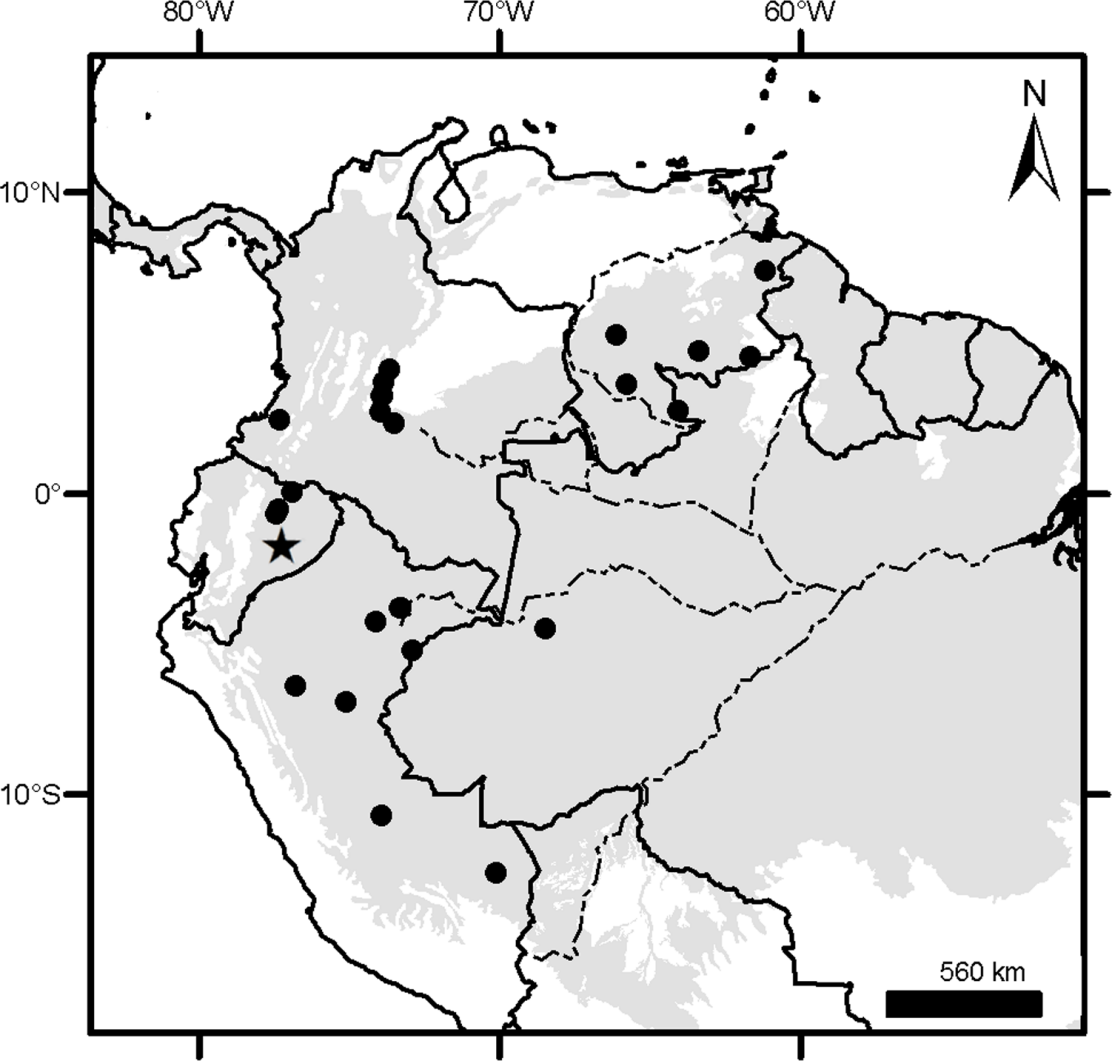
Localities recorded for *Dasypus pastasae*. Gray shading represents the moist broadleaf forest from central and northwestern of South America and dotted lines are the main rivers. Star represents the type locality.

**Distribution:** *Dasypus pastasae* is distributed from the foothills of the eastern Andes in Peru, Ecuador, Colombia, and Venezuela south of the Orinoco River into the western Amazon of Brazil, between the Madeira and Branco rivers (Fig 13). Despite the absence of records from Bolivia, it is likely that *D*. *pastasae* also occurs in the extreme north of that country, on the left bank of the Madre de Dios River. This species is sympatric with *D*. *kappleri* in eastern Venezuela [3].

**Taxonomic history:** Thomas [31] described a new species of armadillo from Ecuador that resembled *D*. *kappleri* and named it *Tatu pastasae*. However, Lönnberg [33] did not consider it different enough to warrant species rank and treated it as subspecies of *D*. *kappleri*, an opinion followed by Cabrera [35], Gardner [38], and Wetzel et al. [1]. On the other hand, Hamlett [34] questioned the validity of this taxon, regarding the differences of *D*. *pastasae* listed by Thomas [31] as individual variation and including Thomas’ species as a synonym of *D*. *kappleri*, a viewpoint followed by Talmage and Buchanan [81] and Wetzel and Mondolfi [36]. Recently, Feijó and Cordeiro-Estrela [3] showed that several characters noted by Thomas [31] and other newly identified cranial traits consistently distinguish it from *D*. *kappleri* Kraus, arguing for it to be accorded a species distinction.

**Fig 14:**
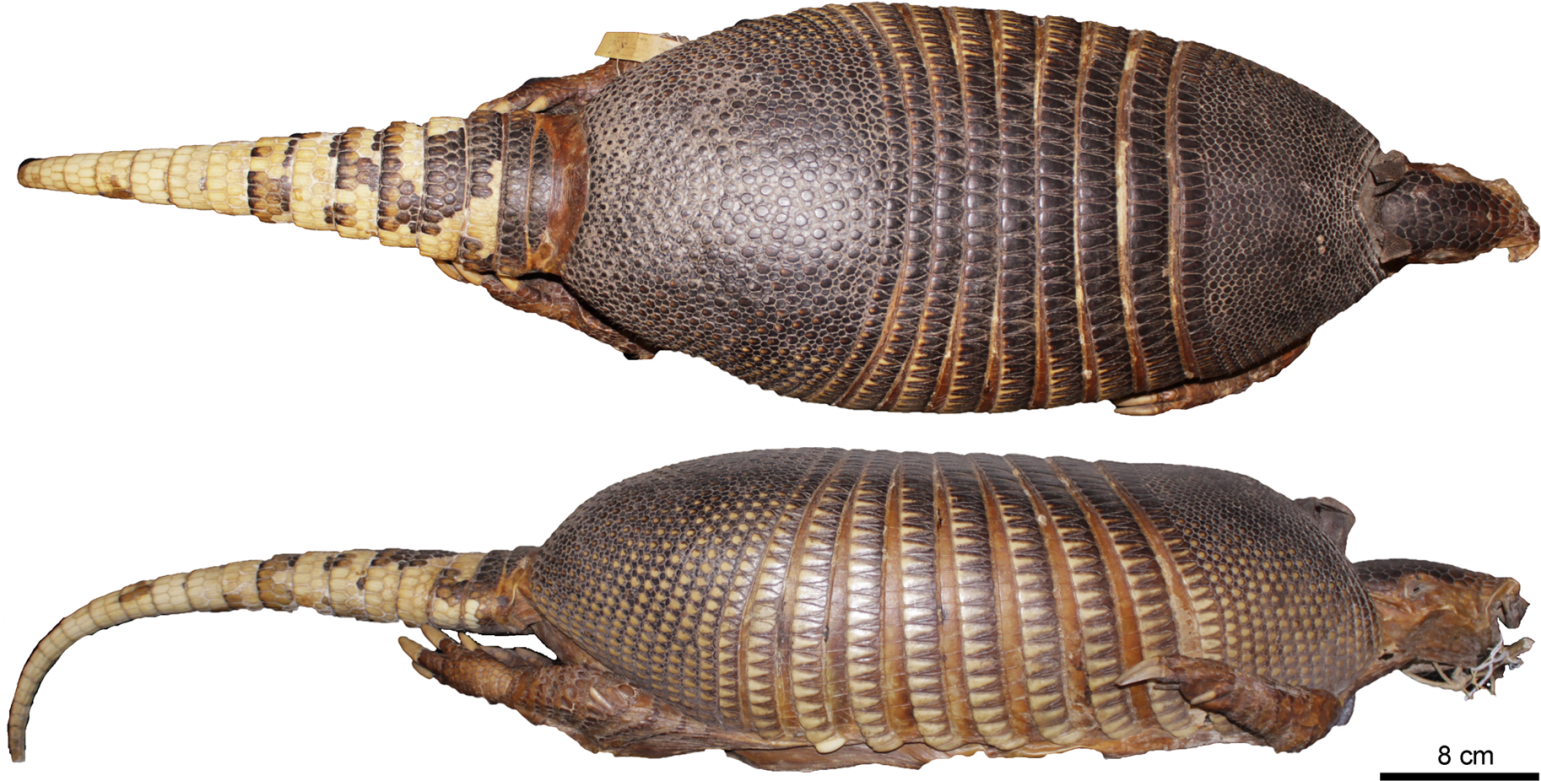
Dorsal and lateral view of the holotype skin, limbs, and tail of *Dasypus kappleri peruvianus* Lönnberg (1928) [= *D*. *pastasae* (Thomas, 1901)].

Lönnberg [33] described a subspecies from Peru, *D*. *kappleri peruvianus*, based on slight cranial differences and on the number of scutes on movable bands and the pelvic and scapular shields. We examined the holotype (Fig 14) of *D*. *k*. *peruvianus* (NRM 631235) as well as other specimens from Peru (MUSM 697, MUSM 23073, AMNH 98812, AMNH 98464, AMNH 76574, AMNH 76573, MUSM 11081, AMNH 268227, AMNH 268228) that resemble the traits exhibited in *D*. *pastasae*. The diagnostic characters mentioned by Lönnberg [33] show no geographic pattern and are subject to intrapopulational variation. Therefore, we agree with previous authors, and treat Lönnberg’ *D*. *k*. *peruvianus* as a junior synonym of *D*. *pastasae*.

**Remarks**: One specimen (QCAZ 3370) from Napo, Ecuador has a peculiar lower crest on the middle portion of the lateral border of the palatine that slightly resembles *D*. *beniensis*, however its posterior end is thin and raised as in typical *D*. *pastasae*. We interpreted this unique shape as individual variation, as another specimen collected at the same locality (QCAZ 3376) shows the typical pattern of *D*. *pastasae*. In one specimen (FMNH 87916) from Meta, Colombia, the anterior lateral portion of the tail shows a subtle posteromedial elevation of some scutes; although different from the typical pattern in the species (i.e., totally flattened), it is not as well marked as in *D*. *kappleri*.

***Dasypus kappleri* Krauss, 1862**

Figs 15 and 16

**Fig 15:**
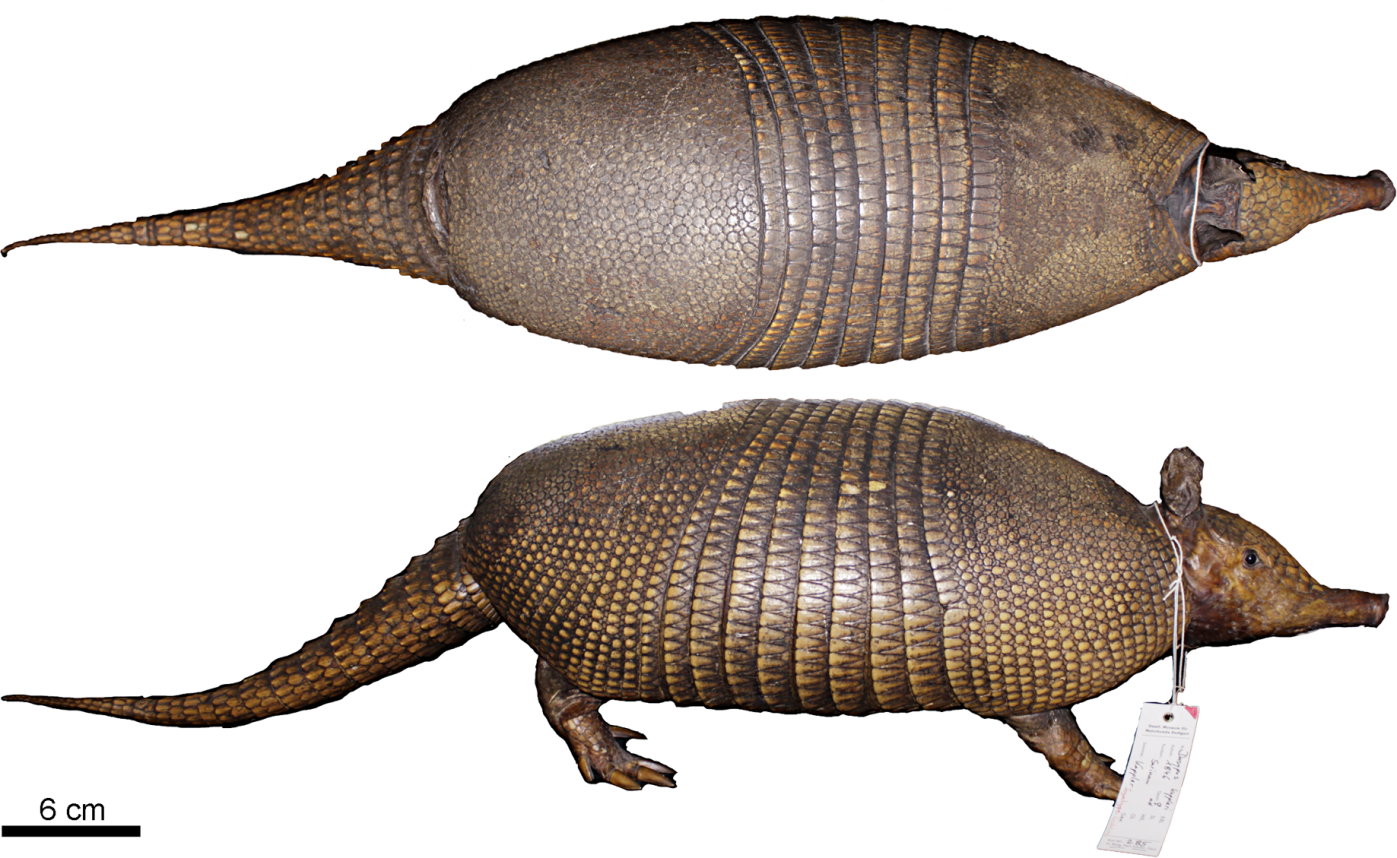
Dorsal and lateral view of the lectotype of the mounted skin of *Dasypus kappleri* (SMN 285).

**Fig 16:**
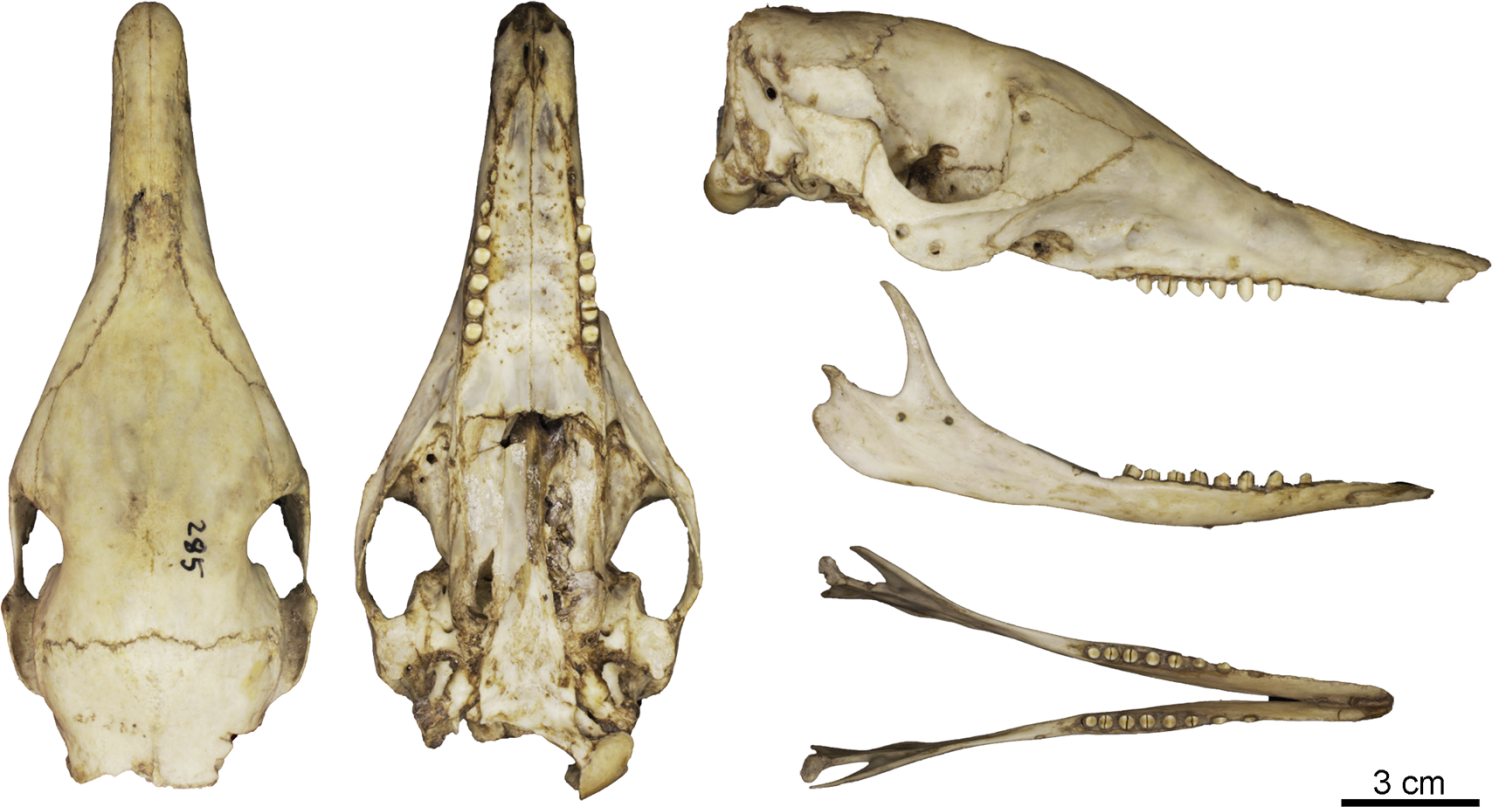
Skull and mandible of the lectotype of *Dasypus kappleri* (SMN 285).

*Das*[*ypus*]. [*p*]*eba*: Burmeister, 1848:199; not *Dasypus peba* Desmarest, 1822

*Dasypus kappleri* Krauss, 1862:20; original description

*Dasypus pentadactylus* Peters, 1864:179; type locality “Guiana”; name based on a specimen identified by Cabanis ([94]; p. 782) as *D*. *peba* from “am Demerara und auf der Savanne am Berbice,” Guyana.

*Praopus kappleri*: Gray, 1873:16; name combination

*Tatusia kappleri*: Thomas, 1880:402; name combination

*[Tatusia (Tatusia)] kappleri*: Trouessart, 1898: 1139; name combination

*[Tatusia (Tatusia)] pentadactylus*: Trouessart, 1898: 1140; name combination

*T*[*atu]*. *Kappleri*: Thomas, 1901:371; name combination

*[Tatus (Tatus)] kappleri*: Trouessart, 1905:814; name combination

*[Tatus (Tatus)] pentadactylus*: Trouessart, 1905:814; name combination

*Dasypus [(Hyperoambon)] kappleri*: Wetzel and Mondolfi, 1979:56; name combination.

**Type:** Krauss [95] listed four specimens of *Dasypus kappleri* (one adult male, one adult female, and two juvenile skulls), collected by A. Kappler in 1846. Wetzel and Mondolfi [36] selected the adult female specimen, SMN[S] 285 (Stuttgart Museum), as the lectotype of *Dasypus kappleri* Krauss, 1862. The lectotype consists of mounted skin and separated skull (palatine and right side of condyle broken) and mandible (Figs 15 and 16).

**Type locality:** “Aus den Urwäldern des Marowiniflusses in Surinam.” Husson [96] suggested that Krauss [95] probably described this species from “the neighborhood of Albina, near the mouth of Marowijne river, Surinam.”

**Diagnosis:** *Dasypus kappleri* is recognized by its large size (see Table 5), absence of a well-defined occipital lobe on the cephalic shield, an angular emargination of the anterior border of the scapular shield, enlarged projecting scales at the knee, and more rings on the tail (covering 78 to 86% of the tail), by its unique pattern of smooth, flattened, and uniform scales on the pelvic shield, with the central and peripheral scales at the same level, and by keeled scales on the proximal tail rings. Cranially, *D*. *kappleri* has a prominent lateral palatine crest raised above the bony surface and its posterior terminus is conspicuously swollen; the posterior border of the palatine is straight and exhibits a prominent, rectangular tentorial process of the parietals (see [3] for a detailed description of diagnostic characters).

**Fig 17:**
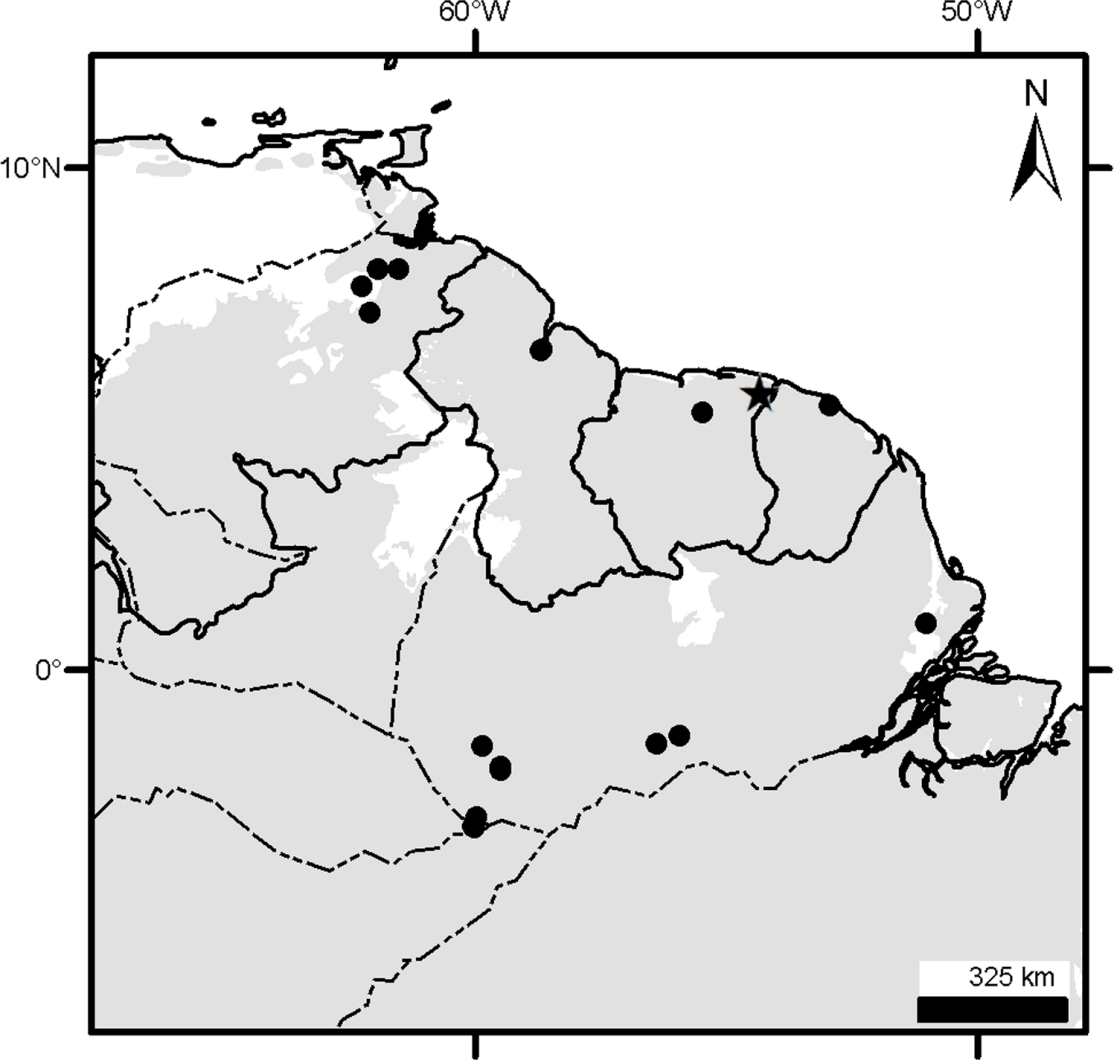
Localities recorded for *Dasypus kappleri*. Gray shading represents the moist broadleaf forest from central and northwestern of South America and dotted lines are the main rivers. Star represents the type locality.

**Distribution:** *Dasypus kappleri* occurs in French Guiana, Suriname, Guyana, eastern Venezuela, and Brazil; in the latter country, it is found east of the Rio Negro-Rio Branco and north of the lower Amazon rivers, and so throughout the entire region known as the Guiana Shield (Fig 17).

**Taxonomic history:** Burmeister [97] was the first to describe a larger armadillo from “Guyana” (Surinam and Cayenne) and named it *Das*[*ypus*]. [*p*]*eba*. However, this same name had been used by Desmarest [98] with reference to *Dasypus novemcinctus* Linnaeus, 1758, which makes Burmeister’s species name unavailable. Later, Krauss [95] provided a detailed description of a new species from Suriname called *Dasypus kappleri*. Interestingly, Krauss [95] made comparisons between *D*. *kappleri* and five specimens housed in the Stuttgart Museum referred by him as Burmeister’s *D*. *pe[b]a*. Nevertheless, Peters [24] claimed that he had access to Burmeister’s original specimens, which undoubtedly represent the species described by Krauss. We had access to the Burmeister’s type at the Martin-Luther-Universität Halle-Wittenberg and agree with Peters’ conclusion. In the same work, Peters [24] also described *Dasypus pentadactylus*, which resembles *D*. *peba* Burmeister but with five digits (not four) on the forefoot. Moreover, he considered these two species distinct enough to justify placing them both in a new genus, *Hyperoambon*. However, Peters overlooked that Krauss had previously mentioned five digits in *D*. *kappleri*, making *D*. *pentadactylus* a junior synonym of *D*. *kappleri*.

The taxonomy of *D*. *kappleri sensu lato*, which formerly included two subspecies [1,35,38], had never been properly revised. Through integrative morphological and morphometric analyses, Feijó and Cordeiro-Estrela [3] recognized three allopatric groups, now restricting *D*. *kappleri* to the Guiana Shield.

**Remarks:** The presence of a fifth digit on the forefoot is usually listed as an exclusive trait of the *kappleri*-group relative to other *Dasypus*. However, we found six adult specimens of *D*. *kappleri*, including the lectotype (MPEG 8907, EBRG 1415, MPEG 7127, SMNS 285, ZMB_Mam_6162, MPEG 7128), and one of *D*. *pastasae* (AMNH 98464) that have only four digits. On the other hand, five specimens of *D*. *novemcinctus* (AMNH 95111, AMNH 208100, EBRG 780, EBRG 2272, EBRG 2271) and one of *D*. *hybridus* (AMNH 205714) have five digits on the forefoot. Remarkably, one *D*. *novemcinctus* (MHNCI 3895) from Paraná, Brazil and one *D*. *kappleri* (EBRG 1490) from El Palmar, Venezuela have five digits on the right forefoot and four on the left. Costa and Vizcaíno [99] reported a fifth digit in a juvenile of *D*. *novemcinctus* and hypothesized that it could be either lost during ontogeny as a consequence of digging behavior or this supernumerary digit could be confined to an individual or a population. Our results refute both hypotheses and suggest that the presence of a fifth digit is probably a plesiomorphic character of *Dasypus* that is preserved in most of the *kappleri*-group specimens, but lost in specimens of most of the other *Dasypus* taxa. We regard this trait as imperfectly diagnostic of the *kappleri*-group, because among 2126 *Dasypus* specimens examined, only 15 (0.7%) deviated from this pattern.

The inflation in the posterior lateral border of the palatine varies in its development, either less inflated (e.g., EBRG 582, MZUSP 19967, MPEG 8907) or more swollen (e.g. AMNH 48132, EBRG 1415, EBRG 1416). This is also true of the development of keels on the posterior border of scutes on the tail, which in some specimens are highly acute and prominent (e.g., EBRG 1490, EBRG 1238, AMNH 64119) and less developed in others (e.g., AMNH 48222, EBRG 582, EBRG 1415, EBRG 1416).

***Dasypus novemcinctus* Linnaeus, 1758**

Figs 18, 19 and 20

**Fig 18:**
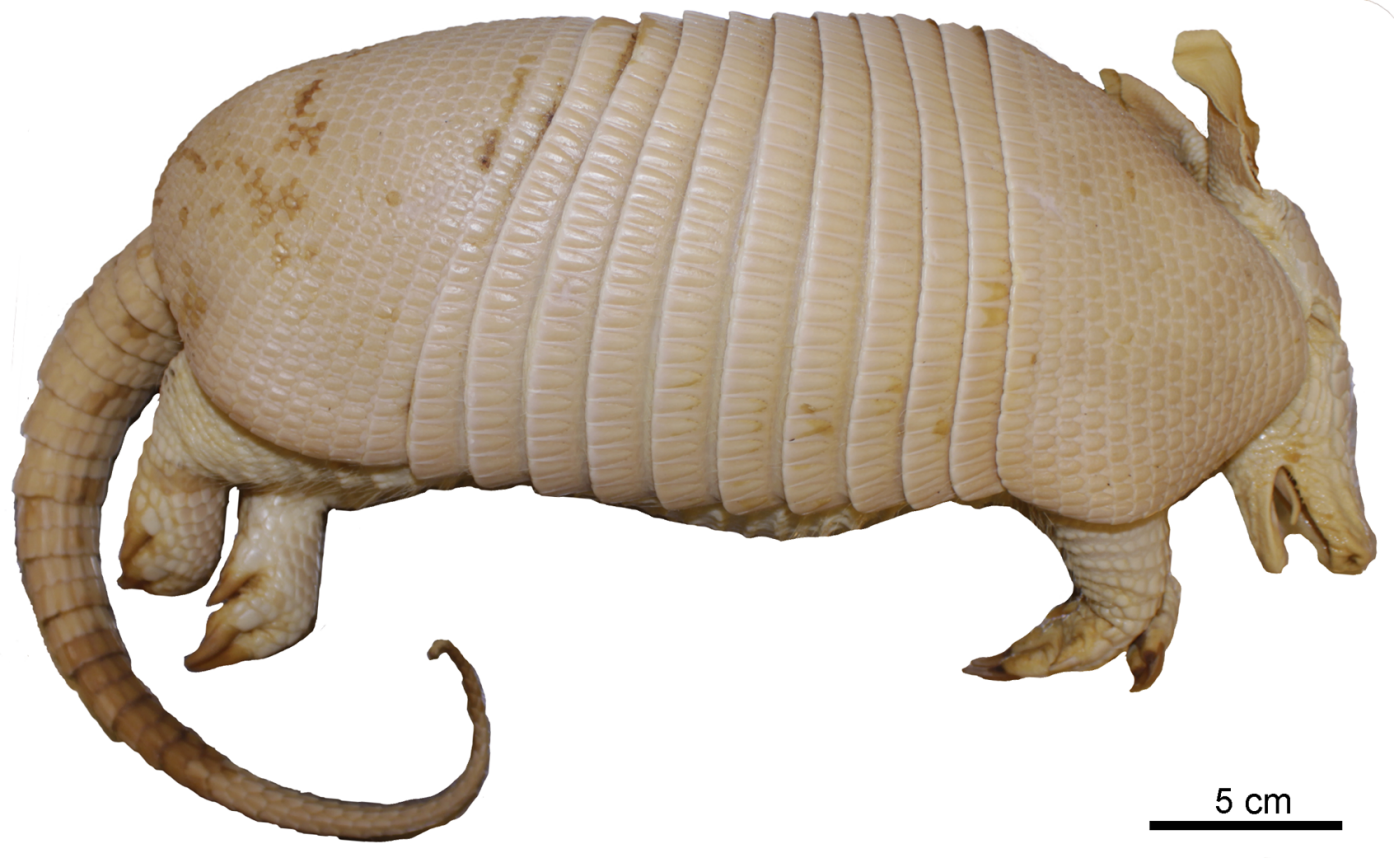
Paralectotype of *Dasypus novemcinctus* Linnaeus, 1758 (NRM 532077), the Linnaeus extant specimen originally preserved in the collection of the King A. Fredrik.

**Fig 19:**
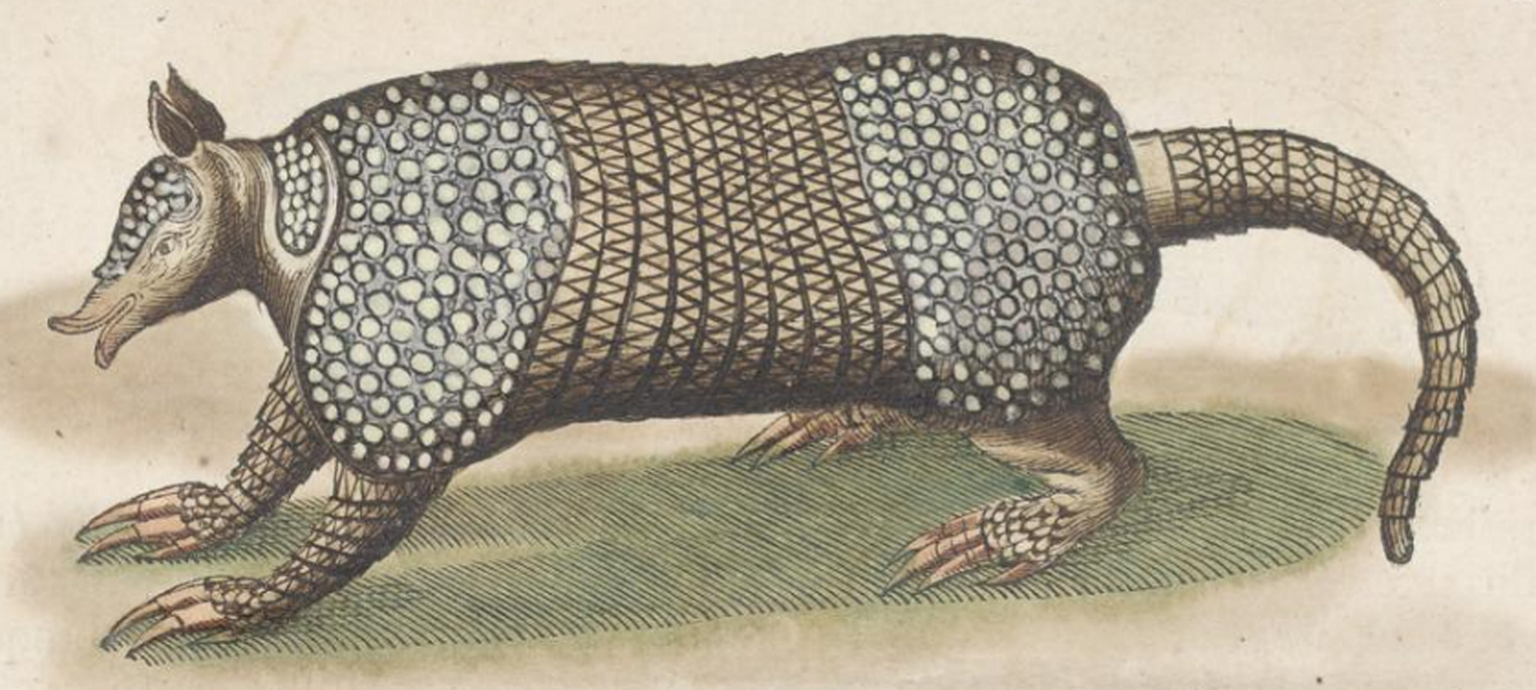
Lectotype of *D*. *novemcinctus* herein designated. Xylogravure reproduced from Marcgrave’s “Tatu-ete” (1648: 231).

**Fig 20:**
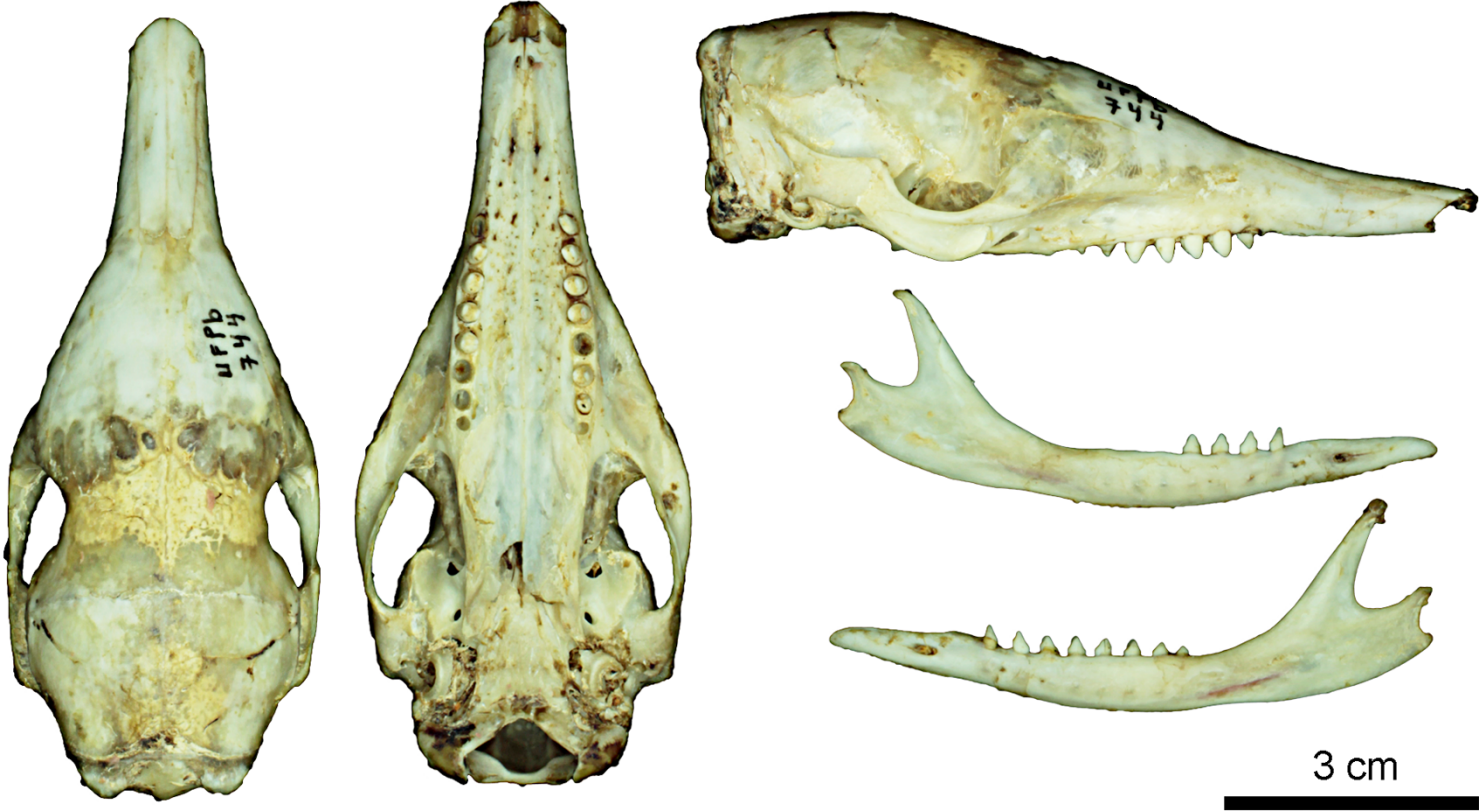
Skull and mandible of the *Dasypus novemcinctus* Linnaeus, 1758 (UFPB 744) from Paraiba, Brazil [ca. 50 km from the type locality].

*[Dasypus] novemcinctus* Linnaeus, 1758:51; original description.

*Tatus minor* Fermin, 1769:110; unavailable name [83].

*Dasypus octocinctus* Schreber, 1774:pl.lxxiii; no locality given.

*[Tatu] Novemcincta*: Blumenbach, 1779:74; name combination.

*Dasypus longicaudatus* Kerr, 1792:112; type locality “America”

*Dasypus novenxinctus* Peale and Palisot de Beauvois, 1796:18; incorrect subsequent spelling of *Dasypus novemcinctus* Linnaeus.

*Dasypus longicaudatus* Daudin in Lacépède, 1802:173; no locality given; based on Buffon’s “*Le tatou à longue queue*” ([100]; p. 168, pl. 22) of unknown province; preoccupied by *Dasypus longicaudatus* Kerr.

*lor[icatus]*. *niger* Desmarest, 1804:28; no type locality given; based on “*Le tatou noir de d’Azara*, *le tatueté et cachicame de Buffon*.”

*[Dasypus] serratus* Fischer, 1814:128; type localities “Paraquaia, inprimis in provincial Buenos-Ayres (Boni Aëris).”

*Dasypus decumanus* Illiger, 1815:108; nomen nudum.

*Dasypus decumanus* Olfers, 1818:219; nomen nudum.

*T[atus]*. *niger* Olfers, 1818:220; type localities “Paraguay, Brasilien”; preoccupied by *Loricatus niger* Desmarest.

*Dasypus niger* Lichtenstein, 1818:20; type locality not given; based on *D*. *novemcinctus* Linnaeus; therefore, the type locality is “Pernambuco, Brazil” (lectotype designated here); preoccupied by *Loricatus niger* Desmarest.

*Dasypus peba* Desmarest, 1822:368; type localities “Le Brésil, le Guyane, le Paraguay…On ne le trouve pas dans la province de Buenos-Ayres.”

*Dasypus longicaudus* Schinz, 1824:253; in synonymy, unavailable name.

*D[asypus]*. *longicaudus* Wied-Neuwied, 1826:531; type locality “In den Waldern am Mucuri”; identified by Ávila-Pires ([101]; p. 12) as Morro d’Arara, Rio Mucuri, Bahia, Brazil.

*Tatusia peba*: Lesson, 1827:311; name combination

*Dasypus [(Cachicamus)] novemcinctus*: McMurtrie, 1831:163; name combination.

*Dasypus uroceras* Lund, 1839[1841]:pl. 12, Fig 5; type locality “Rio das Velhas, Floddal” (p. 73), Lagoa Santa, Minas Gerais, Brazil.

*D[asypus] uroceros* Burmeister, 1848:199; incorrect subsequent spelling of *Dasypus uroceras* Lund.

*Praopus longicaudatus* Burmeister, 1854:298; name combination

*Cachicamus novemcinctus* Degland, 1854:125; name combination

*Dasypus pepa* Krauss, 1862:19; incorrect subsequent spelling of *Dasypus peba* Desmarest.

*D[asypus]*. *Longicaudatus* Peters, 1864:179; incorrect subsequent spelling of *Dasypus longicaudus* Wied-Neuwied; not *D*. *longicaudatus* Kerr.

*D[asypus]*. *longicaudatus* Peters, 1864:179; incorrect subsequent spelling of *Dasypus longicaudus* Wied-Neuwied; not *D*. *longicaudatus* Kerr.

*Dasypus fenestratus* Peters, 1864:180; type locality “Costa Rica”; restricted to San José, Costa Rica by Wetzel and Mondolfi ([36]; p. 50).

*Dasypus novemcinctus* var. Mexicanus Peters, 1864:180; type locality “Mexico”; restricted to Colima, Mexico, by Bailey ([102]; p. 52); later further restricted to Matamoras [*sic*], Tamaulipas, Mexico by Hollister ([103]; p. 60)

*Dasypus mexicanus*: Fitzinger, 1871:332; name combination.

*Dasypus Lundii* Fitzinger, 1871:340; type locality “Brasilien.”.

*Tatusia platycercus* Hensel, 1872:105; type locality “Urwald von Rio Grande do Sul,” Brazil.

*Tatusia mexicana*: Gray, 1873:14; name combination.

*Tatusia granadiana* Gray, 1873:14; type locality “Concordia,” Antioquia, Colombia

*Tatusia leptorhynchus* Gray, 1873:15; type locality “Guatemala”.

*Tatusia brevirostris* Gray, 1873:15; type localities “Rio de Janeiro,” Brazil, and “Bolivia”; type locality not restricted to Rio de Janeiro by Wetzel and Mondolfi ([36]; p. 50), because of their false assumption of holotype (ICZN [104]: Art. 74.5). Wetzel et al. [1] selected the specimen from Rio de Janeiro (skin BM 44.3.7.2; skull: BM 46.5.13.16) as the lectotype.

*Tatusia leptocephala* Gray, 1873:16; type locality “Brazils.”

*Tatusia boliviensis* Gray, 1873:16; type locality “Bolivia.”

*T[atusia]*. *leptorhinus* Gray, 1874:246; incorrect subsequent spelling of *Tatusia leptorhynchus* Gray.

*Praopus 9-cinctus* Burmeister, 1879:434; name combination

*Tatusia novemcincta*: Thomas, 1880:402; name combination.

*Tatusia longicaudatus*: Allen, 1895:187; name combination.

*[Tatusia (Tatusia)] novem-cincta*: Trouessart, 1898:1139; name combination

*[Tatusia (Tatusia)] platycercus*: Trouessart, 1898:1140; name combination.

*[Tatusia (Tatusia)] brevirostris*: Trouessart, 1898:1140; name combination.

*[Tatusia (Tatusia)] leptocephala*: Trouessart, 1898:1140; name combination.

*[Tatusia (Tatusia)] boliviensis*: Trouessart, 1898:1140; name combination.

*[Tatusia (Tatusia)] granadiana*: Trouessart, 1898:1140; name combination.

*Tatua novemcincta*: Robinson and Lyon, 1901:161; name combination.

*[Tatus (Tatus)] novem-cinctus*: Trouessart, 1905:814; name combination.

*[Tatus (Tatus)] platycercus*: Trouessart, 1905:814; name combination.

*[Tatus (Tatus)] brevirostris*: Trouessart, 1905:814; name combination.

*[Tatus (Tatus)] leptocephalus*: Trouessart, 1905:814; name combination.

*[Tatus (Tatus)] boliviensis*: Trouessart, 1905:814; name combination.

*[Tatus (Tatus)] granadianus*: Trouessart, 1905:814; name combination.

*Tatu novemcinctum texanum* Bailey, 1905:52; type locality “Brownsville, Texas”.

*Tatusia novemcincta* var. *mexianae* Hagmann, 1908:29; type locality “Insel Mexiana,” Pará, Brazil.

*Dasypus boliviensis*: Grandidier and Neveu-Lemaire, 1908:5; type locality “environs d’Uyuni,” Potosí, Bolivia; preoccupied by *Tatusia boliviensis* Gray.

*Dasypus novemcinctus hoplites* Allen, 1911:195; type locality “hills back of Gouyave, island of Grenada”, Lesser Antilles.

*Dasypus novemcinctus aequatorialis* Lönnberg, 1913:34; type locality “Peruchu, altitude 7–9,000 feet,” Pichincha, Ecuador.

*D[asypus]*. *longi-cauda* Larrãnaga, 1923:343; type locality “provincial paracuarensi”; based on Azara’s ([105]; p. 144) “Negro”; a junior synonym and homonym of *Dasypus longicaudus* Wied-Neuwied.

*D[asypus]*. *brevirostris*: Yepes, 1933:230; name combination.

*Dasypus novemcinctus davisi* Russell, 1953:21; type locality “Huitzilac, 8500 feet, Morelos, Mexico”.

**Type:** Linnaeus [20] based the name *Dasypus novemcinctus* on five references: “Mus[eum]. Ad[olph]. Fr[idericianu]. 6; Syst[ema]. nat[urae]. 6; Seb[a]. mus. I. p. 45 t. 29 f. 1 & t. 53. f. 6; Marcgr[ave]. bras[iliae]. 231; Raj[o]. 9. quadr[upedum]. 233.; and Hern[andez]. mex[icanorum] 314”. According to Articles 73.2.1 and 72.4.1 of the International Code of Zoological Nomenclature [104], all specimens mentioned in these references used by Linnaeus are syntypes.

The first citation refers to a catalogue of the King of Sweden Adolf Fredrik collection published by Linnaeus [106]. The Adolf Fredrik collection is one of the XVIII^th^ century collections to form the bases of the Swedish Museum of Natural History, which contains many type specimens of animals described by Linnaeus [20,107]. In this collection, there is a specimen (NRM 532077) of *Dasypus novemcinctus* that is preserved from King A. Fredrik's collection (E. Åhlander, collection manager of Swedish Museum of Natural History, pers. comm.) with the characteristics described by Linnaeus ([20]; p. 51): “D. cingulis novem, palmis tetradactylis, plantis pentadactylus” and is, therefore, one of the syntypes of the species. The specimen is an adult male preserved intact in alcohol (with the skull included) and the carapace lacks most of the epidermal scales (Fig 18). However, this specimen lacks any label or field notes regarding its exact origin. According to E. Åhlander (pers. comm.), the major sources of specimens in the King’s collection were from the Dutch East India Company and the Dutch West India Company. The latter included colonies in northeastern Brazil, Suriname, and Guyana, where *Dasypus* occurs. However, the use of the alcohol preservation technique only began after the end of the Dutch colonial period in northeastern Brazil in 1654 (E. Åhlander, pers. comm.). The specimen’s preparation therefore rules out Brazil as the origin of NRM 532077. Although it is impossible be sure about its precise origin, Suriname seems likely, as according to Thomas [32] it was the principal source of Dutch collections from South America.

According to Article 76.2 of the ICZN [104], by selecting a lectotype, its place of origin becomes the type locality of the nominal species-group taxon, regardless of any previously published statement. Therefore, if we select Linnaeus’ specimen NRM 532077 as the lectotype of *D*. *novemcinctus*, the type locality becomes Suriname. Recent mitogenomic data suggest that specimens identified as *Dasypus novemcinctus* from French Guiana represent a separate, distinct lineage from individuals with the same name from the United States and might represent a distinct, undescribed species (see [10]). We have also noted morphological peculiarities among French Guianan specimens (see “Remarks”). Thus, in the interest of nomenclatural stability, we deliberately **do not** select the specimen NRM 532077 as lectotype (but viewed as a paralectotype). Instead, we select the specimen illustrated (Fig 19) by Marcgrave ([108]; p. 231) as lectotype of *Dasypus novemcinctus* Linnaeus, 1758, which was used by Linnaeus to found the species, in agreement of the article 73.2.1 of the ICZN [104]. The illustration depicts an animal with nine transverse bands on the middle of the dorsum formed by triangular scutes, with concentric rings on the tail, and ears at the top of the head, and undoubtedly represents *Dasypus novemcinctus* Linnaeus.

**Type locality**: No precise collection locality of the lectotype illustrated by Marcgrave [108] is known. We suggest “Pernambuco, Brazil” as type locality because it was the main settlement of the Dutch colony in Northeastern Brazil, where Marcgrave spent most of his time [32].

**Diagnosis:** *D*. *novemcinctus* is a medium-sized armadillo (mean TL: 746 mm; see Tables 5 and 6), with 8–10 movable bands at the dorsum of the body, 51–67 number of scutes on the 4^th^ movable band, 56–73 on the posterior border of the scapular shield, well-defined occipital lobe on the cephalic shield, absence of enlarged scales on the knee, rounded lateral border of the palatine, and sigmoid dorsal profile of the skull (Fig 20).

**Fig 21:**
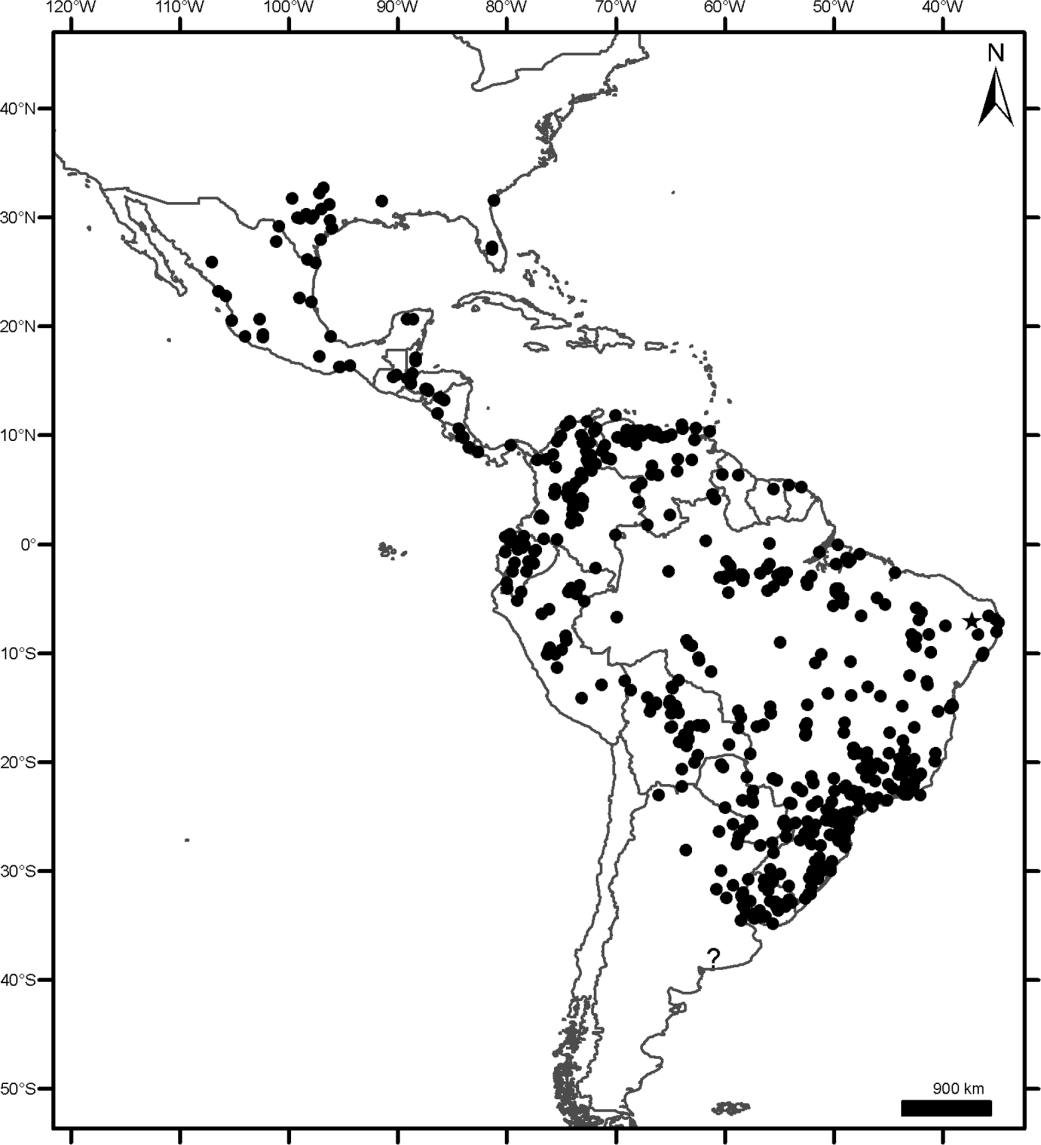
Localities recorded for *Dasypus novemcinctus*, showing specimens examined. Question mark: doubtful record from Coronel Dorrego, southeastern Buenos Aires province, Argentina. Star represents the region of the type locality.

**Distribution:** The species is distributed from Argentina to the USA, covering an area of 19,100,000 km^2^ [109] between the latitudes 38°S and 43°N [4], making it the most widely-distributed species of Xenarthra (Fig 21). Its northern distributional limits have rapidly expanded over the last century, mainly due to anthropogenic influences [4,110,111,112]. The nine-banded armadillo was originally restricted to the south bank of the lower Rio Grande, which delimits the border between Mexico and the United States (but see [113]). Recent records show that the current northern distributional limits lie in southern Wisconsin, and northern Iowa (around 43° N), and it ranges as far west as central New Mexico (around 104° W). The southernmost record of the species comes from Coronel Dorrego, southeastern Buenos Aires province, Argentina, and is based on a single animal (MLP 1.I.03.9) found alive in May 2006 [114]. Without previous records in nearby areas, this specimen might have been introduced [114,115]. Excluding this dubious record, the southern limit of the species’ distribution appears to be around 35°S, coinciding with Canelones, Uruguay and Buenos Aires, Argentina.

*D*. *novemcinctus* is an ecologically tolerant species, inhabiting a wide variety of environments, from places where temperatures fall below 0°C during the winter to those that exceed 40°C in the summer, as well as semiarid habitats with practically no precipitation in the dry season to dense humid forest with as much as 692 mm of precipitation in the wettest month. Its elevational range goes from sea level to nearly 4000 meters. However, despite its widespread distribution, the species is absent in arid zones, such as the Central Plateau of Mexico, Sonoran Desert, and western Texas, each of which acts as a barrier to dispersal for the nine-banded armadillo. The species is also absent from the Llanos of Venezuela and Colombia (Fig 21), probably due to competition with *D*. *sabanicola*.

**Taxonomic history:** The taxonomic history of the nine-banded armadillos is the most complex among extant Xenarthra. Its wide distribution coupled with the high intrapopulational morphological variation and its ease of capture are probably the main factors responsible for its long list of synonymies. At least 19 related taxa have been described since the XIX^th^ century. In his monographic listing of Xenarthra (and other taxa) housed at the British Museum, Gray [25] at once described four new species from South America (*Tatusia granadiana*, *Tatusia brevirostris*, *Tatusia leptocephala* and *Tatusia boliviensis*) and two (*Tatusia mexicana* and *Tatusia leptorhynchus*) from Central America. Years before, Peters [24] had described *Dasypus fenestratus* from Costa Rica. Those descriptions were based mainly on minor variations in carapace shape, cranial proportions, and variable cranial traits. After examining large series of specimens from Central and South America, Alston [29] stated that “Armadillos [*D*. *novemcinctus*] show so much variation in minor cranial characters that I cannot regard these [supposedly diagnostic] differences [listed by Peters and Gray] as of any real value. Accordingly we find that they are not all constant in Central-American specimens.” According to Alston [29], *Dasypus novemcinctus* was the only species of nine-banded armadillo from Texas to Paraguay. Lönnberg [33] provided detailed comparisons between most taxa of *Dasypus* then recognized, showing that many of their presumed diagnostic traits show intraspecific variation.

Hamlett [34] stated that any discussion regarding the status of the species and subspecies of *D*. *novemcinctus* related could be only made after analyses of individual variation within a large series from a single locality. Nevertheless, Cabrera ([35]; p. 224) provisionally recognized three subspecies of *D*. *novemcinctus* from South America, while Hall [116] recognized four subspecies from Central and North America. McBee and Baker [117] recognized only six subspecies, excluding *D*. *n*. *mexianae* from Cabrera’s list. Wetzel and Mondolfi [36] did not recognize or discuss any intraspecific subdivisions. Gardner [38] also listed six subspecies, but included *D*. *n*. *mexianae* and excluded *D*. *n*. *davisi* (from south-central Mexico). Subspecies were typically described based on one or few specimens without any broad geographic comparisons. Therefore, a comprehensive study aimed at testing the geographic races within the nine-banded armadillo is still needed.

**Remarks:** The number of movable bands in the nine-banded armadillos typically ranges from eight to nine, although it sometimes reaches ten. However, one specimen from Roque, Peru (NRM 582328) and one from São João do Glória, Minas Gerais, Brazil (MN 10088) were the only *D*. *novemcinctus* examined that showed seven movable bands on the dorsal midline of the body. This condition seems to result from an anomalous attachment of the last and first movable bands to the adjacent shield, because both specimens possessed the regular eight-band pattern on the flanks.

We noted, however, a non-random distribution of the eight- and nine-banded patterns in South America. There is a predominance of eight bands in the eastern and central portion of the distribution of *D*. *novemcinctus*, including Argentina, Uruguay, Paraguay, Bolivia and Brazil, up to the right bank of the Amazon River. However, in Peru, Ecuador, Colombia, Venezuela, the Guianas and northwestern Brazil, nine-banded animals predominate. In North and Central America, there is no clear pattern.

In *D*. *novemcinctus*, there is remarkable variation in the development of the pterygoid, which ranges from poorly developed and restricted to the lateral side of choanae (e.g. MN 2432, ICN 16397) to extremely well-developed and covering the entire posterior border of palatine (e.g. CBF 3963).

The only specimen (EBRG 29404) from the Paraguana Peninsula, northwestern Venezuela, exhibits some peculiar traits. Its entire carapace has protruding scutes, exhibiting a strongly granulose and rough texture; the occipital lobe is barely separated from the main cephalic shield; and there are medium-sized projecting scales at the knee. Another specimen (IAVH 3914) from Riosucio, Chocó, Colombia shows a reduced cephalic shield, covering only 2/3 of the dorsal surface of the head.

A number of specimens (not all) from the Guiana Shield possess a very robust skull, with the frontal well developed and inflated, the maxilla well developed and expanded laterally, a wide, rectangular palate, robust teeth, premaxillary-maxillary suture well beyond the incisive foramina, a markedly sigmoidal dorsal profile of the skull, and vertically expanded jugal. Interestingly, Gibb et al. [10] reported a distinct lineage of *D*. *novemcinctus* from French Guiana that they thought might represent an unknown species. In the same fashion, Guiana specimens were assigned to have peculiar configuration of the paranasal cavities [118] and cranial shape [119] in relation to the non-Guiana populations. Nonetheless, these traits are not shared by all specimens found in the Guiana Shield, some specimens in eastern Venezuela (EBRG 1034, EBRG 1888) and northern Brazil (MPEG 20152, MPEG 20159, MN 30484) are similar to the non-Guiana specimens, including their paranasal sinuses pattern. It is norteworthy, however, that such variant Guiana specimens are restricted to the peripheral area of the Guiana Shield. Therefore, further analyses including other morphological characters not assessed in this present revision together with phylogenetic analyses that include additional samples from South America (mainly northwestern Amazonia) will be needed to clarify the relationship among nine-banded armadillos from the Guianas and non-Guianan Amazonia.

Variation in body size throughout the distribution of nine-banded armadillos is conspicuous and triggered by a complex interaction of distinct selective pressures. According to Feijó [120], specimens at higher latitudes–both north and south—tend to be of medium size, whereas the largest and the smallest individuals inhabit lower latitudes. Thermal resistance, metabolic rate, and starvation time seem to be the main factors controlling body-size variation with latitude. Individuals of medium body mass appear to represent the best energetic compromise to survive the extended cold weather of temperate zones; they exhibit longer starvation times and lower metabolic rates than smaller individuals, but smaller total energy budgets than larger armadillos. Specimens from the dry seasonal forests and savannas tend to be small, while the largest specimens of *D*. *novemcinctus* are found in the climatically-stable rainforests of Amazonia. One possible explanation is the high seasonality in the dry forests, which drastically reduces insect availability and favors smaller individuals with lower overall energetic demands. Alternatively, evergreen forest areas appear to favor larger animals [120].

***Dasypus pilosus* (Fitzinger, 1856)**

Figs 22 and 23

**Fig 22:**
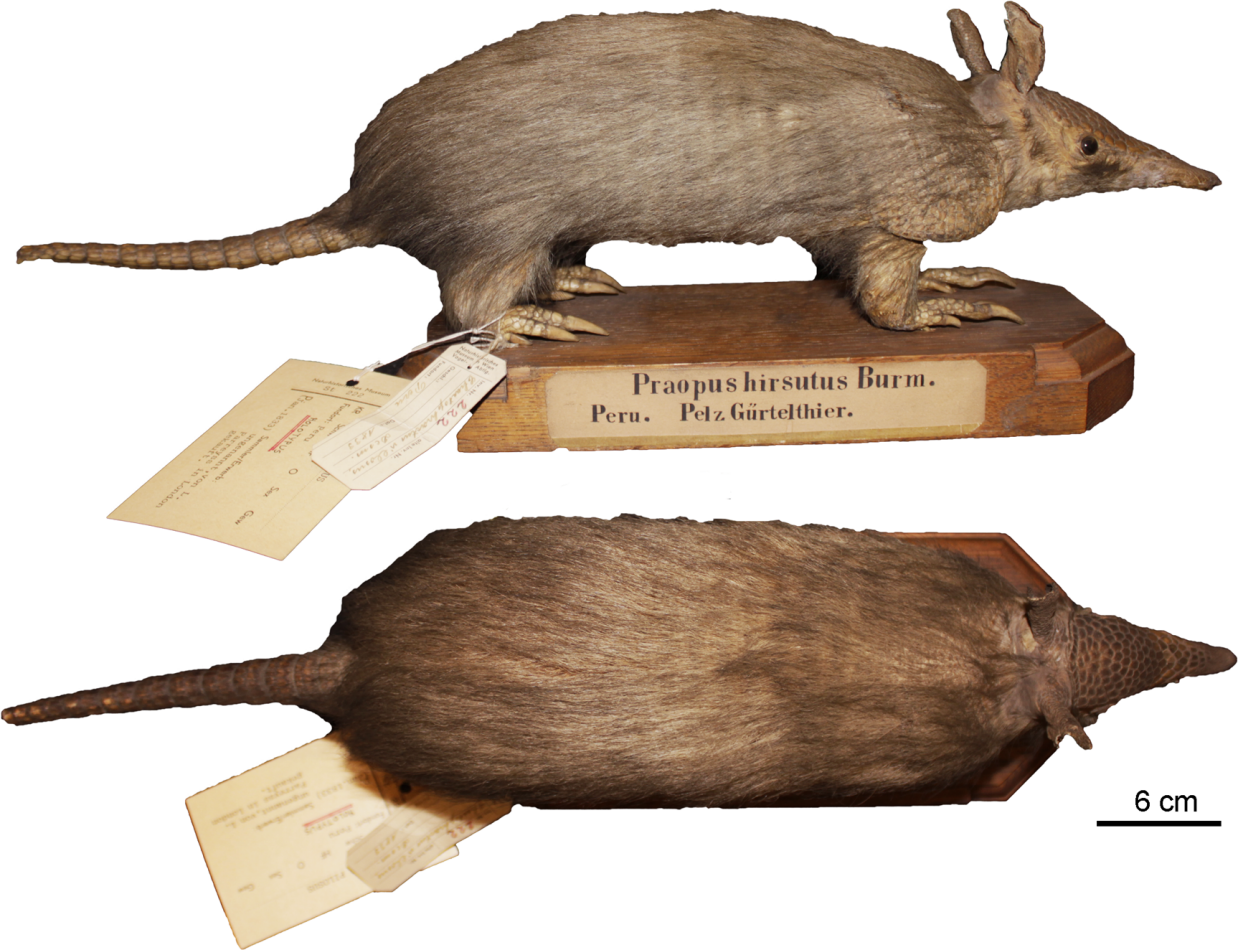
Lateral and dorsal view of the holotype of *Dasypus pilosus* (NMW ST 222).

**Fig 23:**
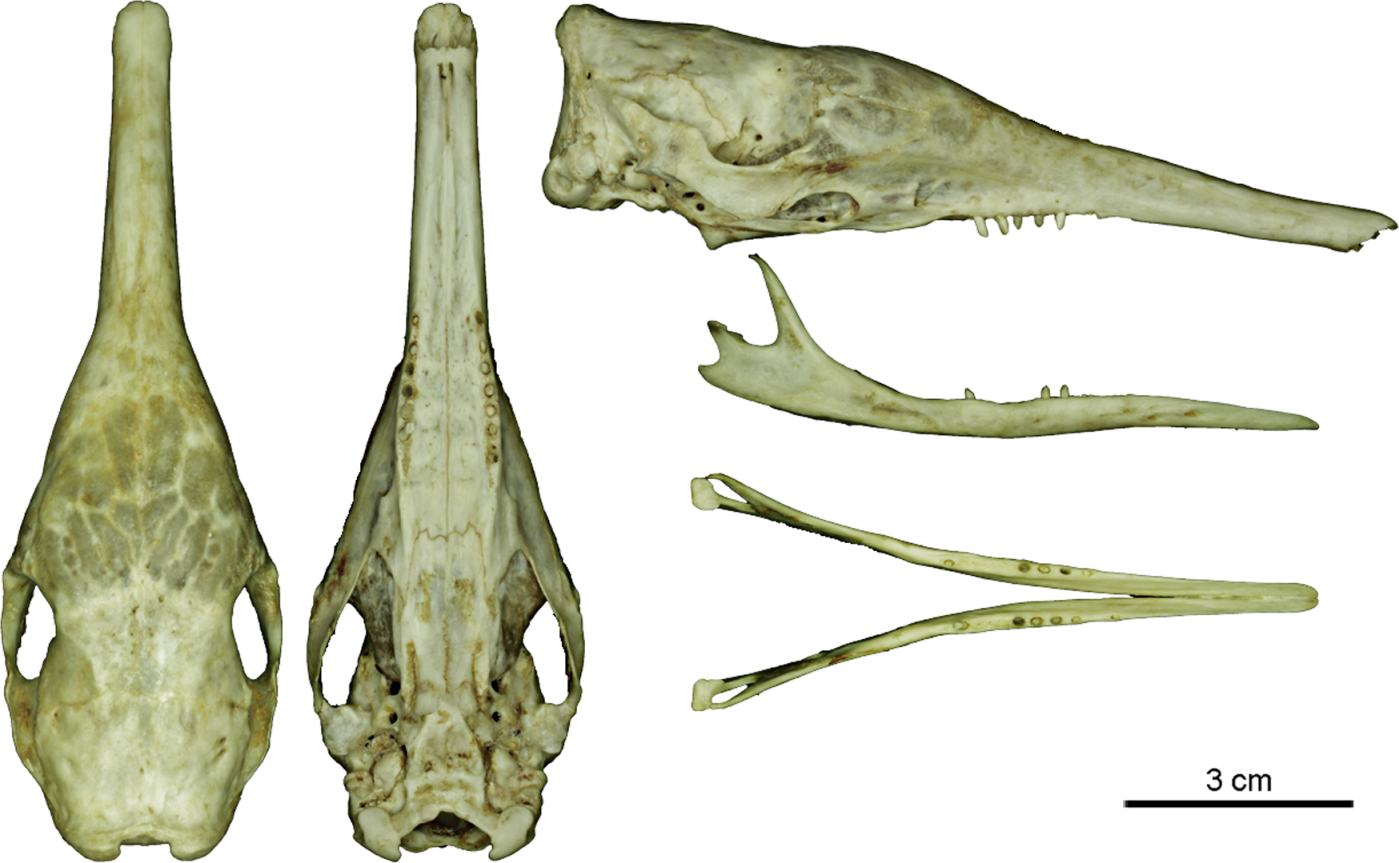
Skull and mandible of *Dasypus pilosus* from Zapatogocha, Huánuco, Peru (MUSM 2056).

*Cryptophractus pilosus* Fitzinger, 1856:123; original description.

*Praopus hirsutus* Burmeister, 1862:147; type locality “Guayaquil,” [Peru?].

*Tatusia pilosa* Flower, 1894:655; name combination.

*[Tatusia (Cryptophractus)] pilosa*: Trouessart, 1898:1140; name combination.

*[Tatus (Cryptophractus)] pilosus*: Trouessart, 1905:814; name combination.

*Tatu pilosa*: Thomas, 1927:605; name combination.

*Dasypus pilosa*: Yepes, 1928:468; name combination with incorrect gender agreement.

*Dasypus pilosus*: Frechkop and Yepes, 1949:27; gender agreement correction.

*Dasypus (Cryptophractus) pilosus*: Talmage and Buchanan, 1954:84; name combination.

*Crypophractus pilosus*: Castro et al. [6]: 34

**Type:** The holotype (NMW ST 222) by monotypy was purchased in London by L. Parreyss in January 1833 (Fig 22). The specimen consists of a well preserved mounted skin (the tip of the tail is broken). Fitzinger [90] stated that the skull is included in the skin.

**Type locality**: “Peru”, restricted to “montane Peru” by Wetzel and Mondolfi ([36]; p. 58).

**Diagnosis:** *D*. *pilosus* is distinguished from other *Dasypus* by a series of unique character states: carapace totally covered by dense long yellowish hair; 9–11 movable bands; very elongated and narrow cephalic shield; osteoderms with numerous, large foramina and without differentiation between their central and peripherals areas; very elongated rostrum and palate; diminutive teeth; and ventral portion of the rostrum concave (Fig 23).

**Fig 24:**
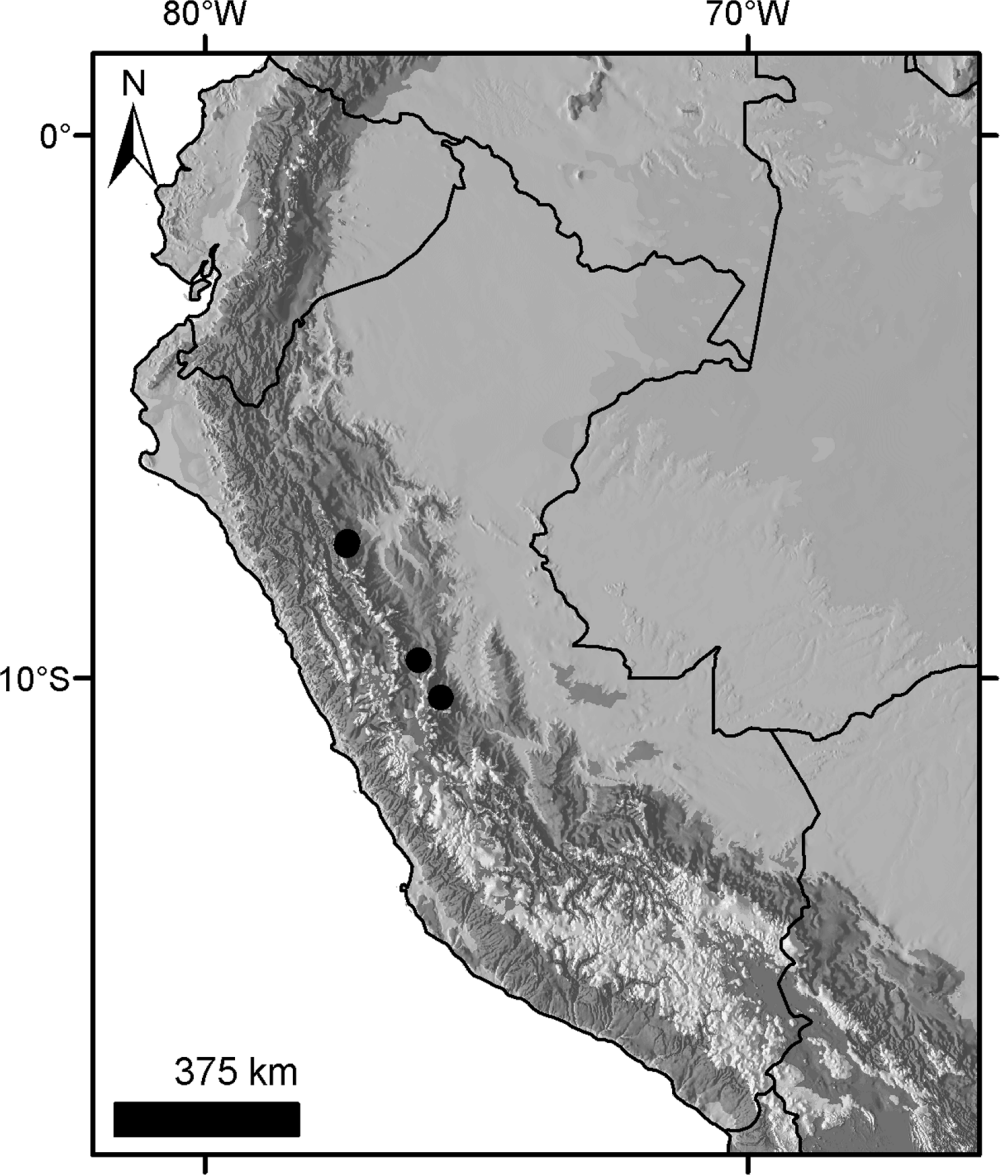
Localities recorded for *Dasypus pilosus*, all restricted to the Peruvian Andes.

**Distribution:** The species is endemic to montane cloud forests and jalca of Peru (Fig 24), occurring between 2600 m to 3400 m according to Castro et al. [6]. Fitzinger (1871) added Colombia, Ecuador, Chile; and Cabrera [35] included Bolivia to *D*. *pilosus* distribution. Nevertheless, available records are restricted to only six departments in Peru, including Amazonas, La Libertad, Huánuco, Junín, Pasco, and San Martín [1,6].

**Taxonomic history:** Fitzinger [90] designated a new genus and species of armadillo named *Cryptophractus pilosus*. The specimen (Fig 22) was so peculiar that Fitzinger [90] speculated that it might represent a transition from an armadillo to an anteater. Six years later, Burmeister [121], unaware of Fitzinger’s species, described *Praopus hirsutus* based on two specimens from “Guayaquil” [no country given] housed at the National Museum in Lima, Peru and for the first time provided an illustration of the hairy armadillo ([121]; p. 149). Wetzel and Mondolfi [36] stated that the type is probably one of two mounted specimens, MHN (now MUSM) 26 or 27, then preserved in the Museo de Historia Natural “Javier Prado.” According to Castro et al. [6], there is now only one mounted specimen (MUSM 27) in that museum, and those authors questioned its relation to the type of *P*. *hirsutus* because the museum was not founded until 1918. However, the resemblance of the preserved specimen (MUSM 27) to the animal illustrated by Burmeister ([121]; p. 149) is noteworthy: they both share the broad spoon-shape of the ears and similar position of the feet, which seems to support the conjecture of Wetzel and Mondolfi [36]. Therefore, if additional evidence confirms that Burmeister studied this mounted specimen, it should be selected as the lectotype of *Praopus hirsutus* Burmeister, 1862. Fitzinger [122] provided a detailed description of his *C*. *pilosus*, comparing it with other *Dasypus* species and including Colombia, Ecuador, Peru, and Chile in its distribution. Flower [123,124] was the first to allocate this species to the same genus of the nine-banded armadillo, naming it *Tatusia pilosa*. Yepes [125] finally placed it in the genus *Dasypus*. Recently, Castro et al. [6] proposed placing it back in its own genus *Cryptophractus* based on its exclusive traits and its external position in their cladistic morphological analysis. However, Gibb et al. [10], in their mitogenomic phylogeny, showed that *D*. *pilosus* is one of the youngest species-rank lineages of *Dasypus*, and its recognition in a distinct genus would render *Dasypus* paraphyletic.

**Remarks:** The hairs are dense, harsh, and longer on the sides of the carapace than on the dorsum, and longer on the pelvic shield than on the scapular shield. Nevertheless, in some animals the difference is more remarkable (e.g. MUSM 7499), while in others it is less evident (e.g. MUSM 2056). The color of the hair changes with age from juvenile to adult. Young specimens are grayish brown, whereas adults are golden brown. Cranially, some specimens possess a small, faint sagittal crest on the posterior portion of the parietal (e.g. MUSM 7500, MUSM 7505).

*Dasypus pilosus* is one of the least studied species of extant Cingulata [126], with virtually no information about its ecology, reproduction, and physiology. Castro et al. [6] suggested a predominantly myrmecophagous diet based on its morphological features. Interestingly, in an elevational study of termite diversity in Peru, Palin et al. [127] found the upper limit to termite distribution is 1500 m for soil-feeding termites and 1850 m for wood-feeding termites. In an elevational study of invertebrates in primary forest in Panama, Olson [128] reported very low abundances of ants, spiders, and beetles over 1250 meters. Therefore, it seems contradictory that a species could specialize on a myrmecophagous diet in a biome mostly lacking ants and termites.

On the other hand, a vermivorous diet is more likely. Some Asian rodents endemic to montane forest (e.g. *Rhynchomys*, *Sommeromys*, *Paucidentomys*) share similar morphologic traits with *Dasypus pilosus*, e.g. very elongated rostrum and palate; delicate and diminutive teeth; slender and straight mandible. A montane forest shrew-rat from Indonesia (*Paucidentomys vermidax)* has an earthworm diet [129], and the montane forest rat *Rhynchomys tapulao* from the Philippines also eats worms and mostly soft-bodied invertebrates [130]. At 2600 m in the primary forest of Costa Rica, litter invertebrates at highest abundance include Gastropoda, Diplopoda, Dermaptera, pupae of Coleoptera, and the soil invertebrates include Oligochaeta, Diplopoda, larvae and pupae of Coleoptera, and larvae of Diptera [131]. Both morphology and invertebrate ecology support our hypothesis that *Dasypus pilosus* likely bases its diet on soft-bodied invertebrates. Field studies could clarify this question and provide new evidence that could be used to explain its unique morphologic traits.

Castro et al. [6] hypothesized that the numerous unique traits exhibited by *D*. *pilosus* reflect its extended divergence from other *Dasypus* species. In contrast, Gibb et al. [10] showed that *D*. *pilosus* was one of the last species of *Dasypus* to diverge from its sister-group, *D*. *novemcinctus*, which was estimated to have taken place 2.8 Ma. We propose a different explanation of the numerous exclusive characters exhibited by *D*. *pilosus*. According to Renaud et al. [132], morphological distinctions may result from strong environmental selective pressures; these are expected to exert greater influence on ecological specialists. *Dasypus pilosus* occurs only in cloud forests of Peru (above 2600 m) and seems to be a good example of a specialized taxon with numerous unique attributes that apparently evolved in response to ecological selective pressures. Indeed, many of the remarkable traits of *D*. *pilosus* could be interpreted as adaptations to both cold, humid weather (abundant hair and many large foramina in their osteoderms) and higher abundance of soft-bodied invertebrates (delicate teeth, elongated palate and rostrum, reduced areas for muscle attachment).

***Dasypus sabanicola* Mondolfi, 1968**

Figs 25 and 26

**Fig 25:**
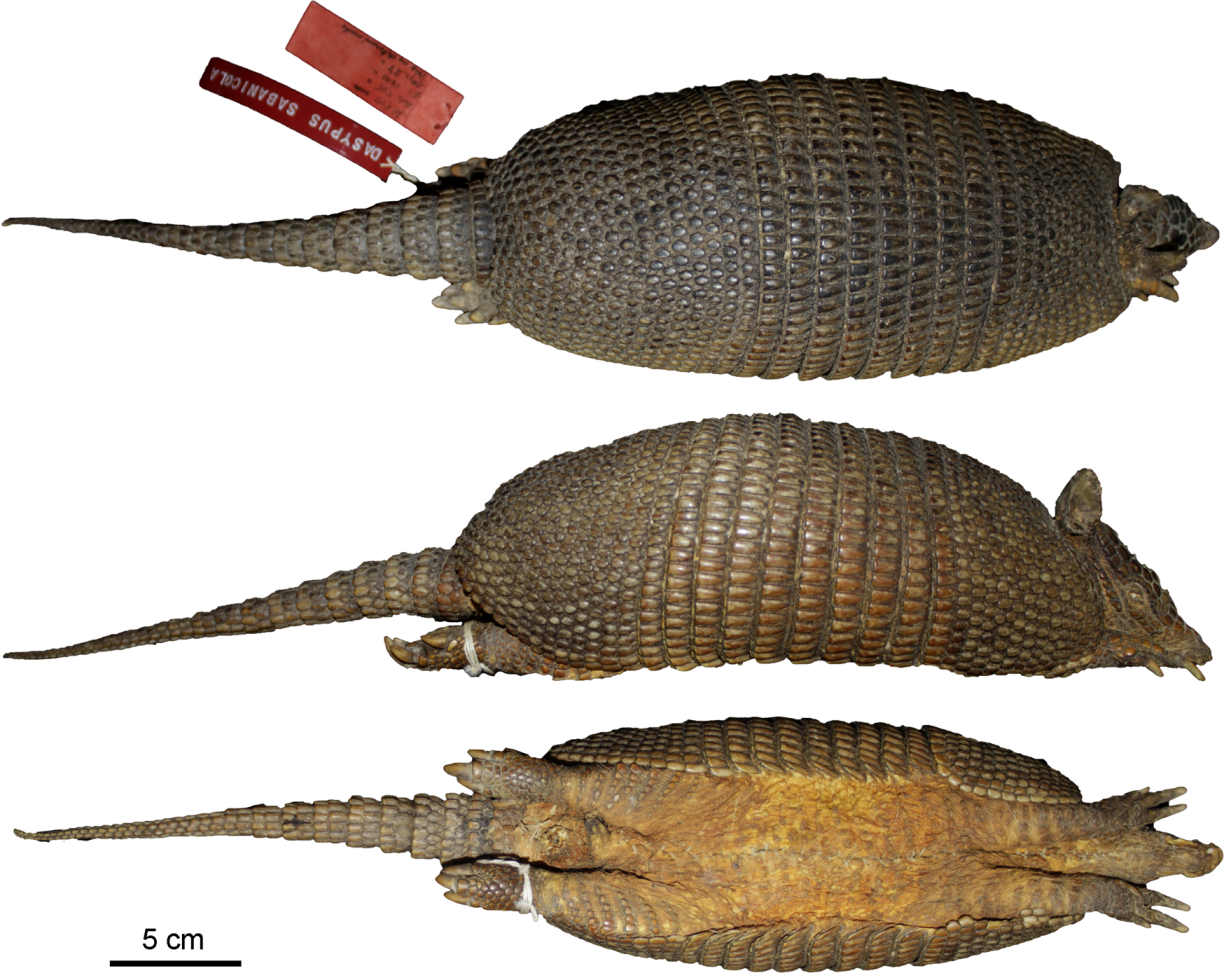
Dorsal, lateral and ventral views of the skin of the holotype of *Dasypus sabanicola* (EBRG 965).

**Fig 26:**
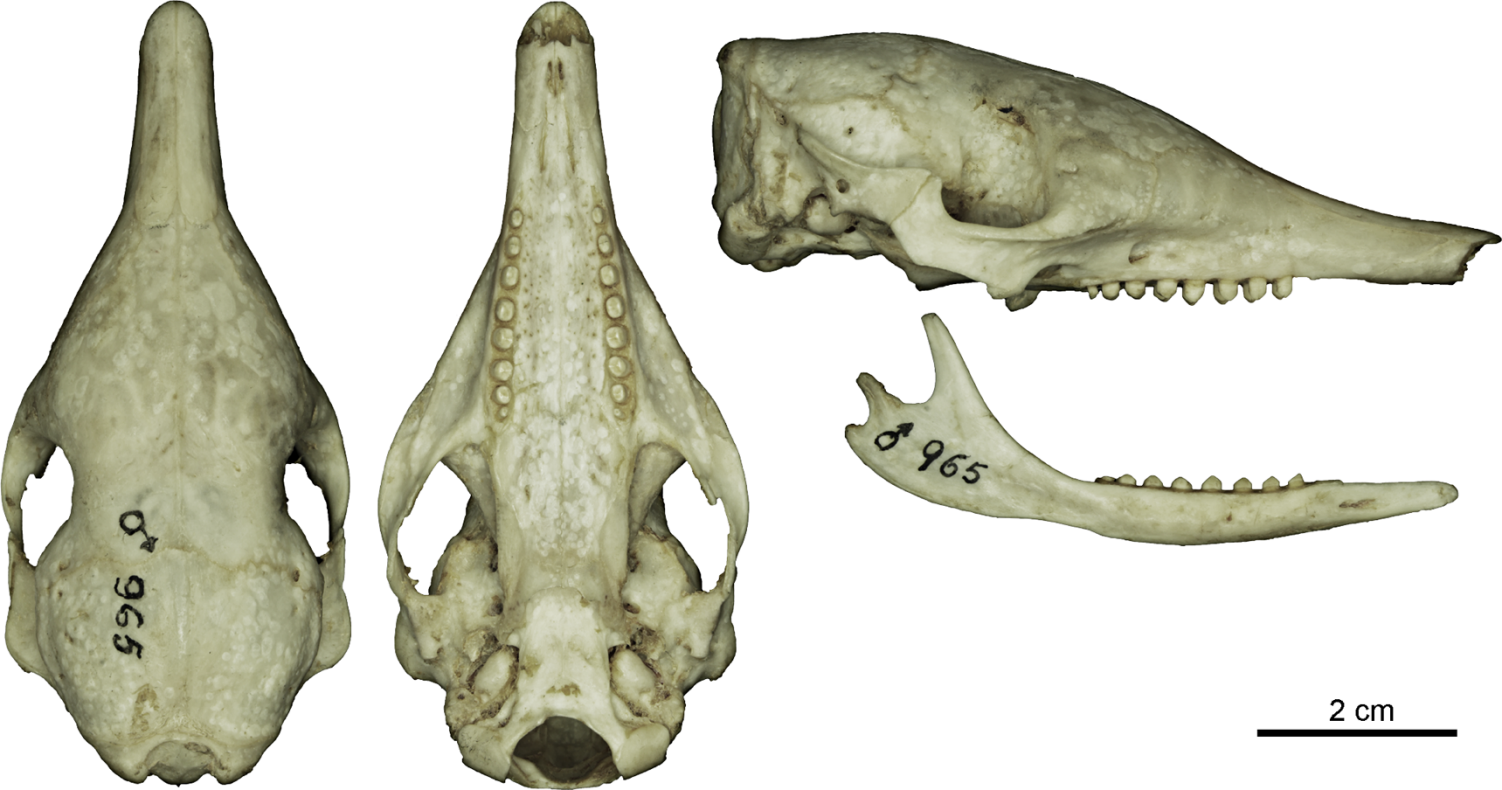
Skull and mandible of the holotype of *Dasypus sabanicola* (EBRG 965).

*Dasypus sabanicola* Mondolfi, 1968:151; original description.

*Dasypus [(Dasypus)] sabanicola*: Wetzel and Mondolfi, 1979:55; name combination.

**Type:** The holotype (M.E.B.R.G 965, nowadays EBRG 965) designed by Mondolfi [133] consists of skin (Fig 25) and separated skull (Fig 26) of an adult male collected by Juhani Ojasti on 18 January, 1968. Mondolfi [133] listed other four adult males (EBRG 783, EBRG 963, EBRG 966, EBRG 967), one adult female (EBRG 964) and one subadult male (EBRG 968) as paratypes.

**Type locality**. Hato Macanillal, Distrito Achaguas, Estado Apure, Venezuela.

**Diagnosis:** *Dasypus sabanicola* is a small-sized species (mean TL: 505 mm; see Tables 5 and 6), with 7–9 movable bands, 47–58 scutes along the posterior border of the scapular shield, 46–56 scutes on the 4th movable band, 10–14 rings on the tail, four digits on the forefoot; and a sigmoidal dorsal profile of the skull in lateral view.

**Fig 27:**
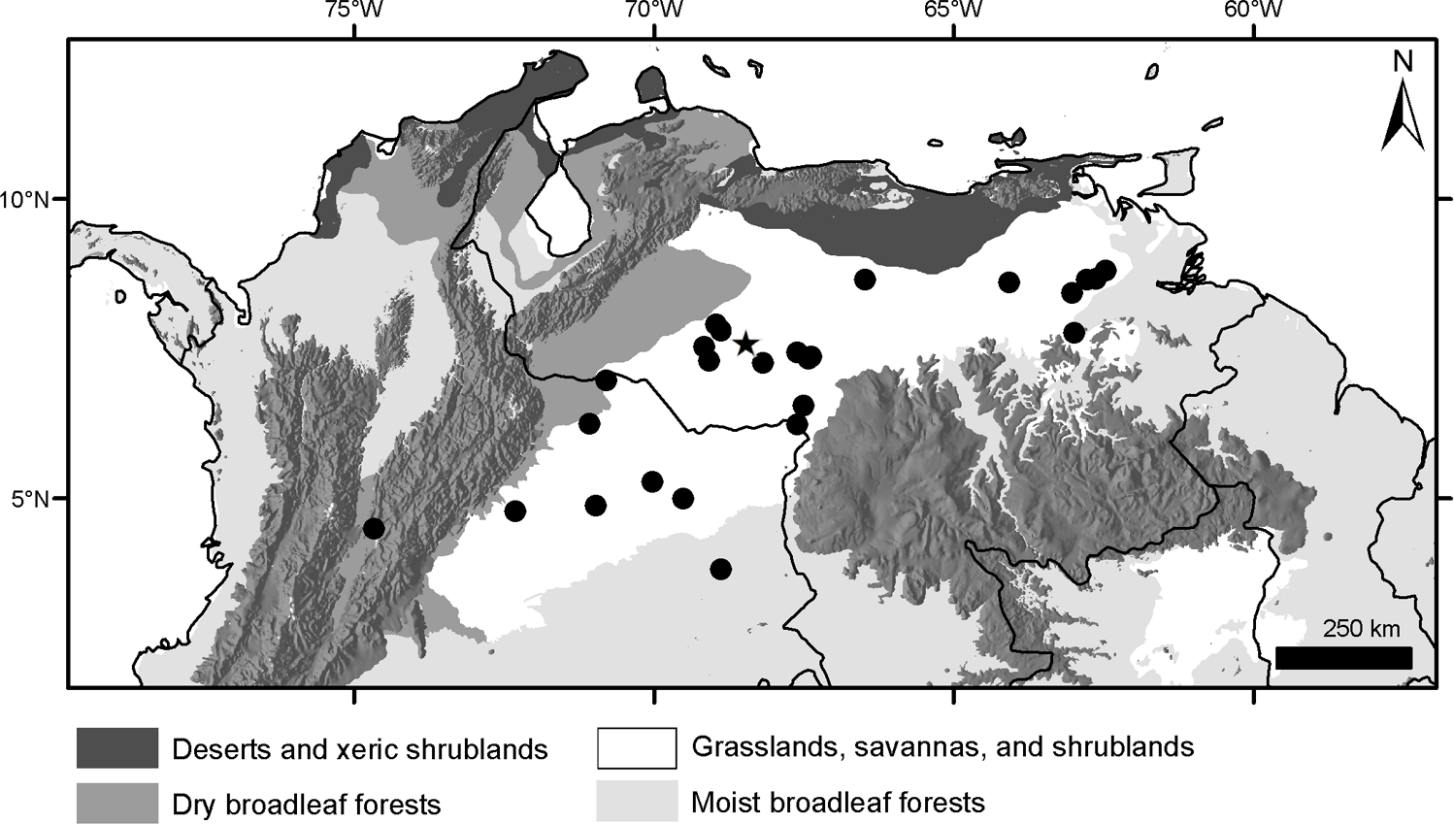
Localities recorded for *Dasypus sabanicola* in the Llanos of Venezuela and Colombia. Star represents the type locality.

**Distribution:** *D*. *sabanicola* is the smallest species of the genus and is restricted to the Llanos of Colombia and Venezuela, one of the largest areas of savanna in South America (Fig 27). The only record outside the Llanos comes from a subadult female (ICN, 1624) collected in Tocaima, Cundinamarca, Colombia. This locality is situated on western slope of central Eastern Andes on Rio Bogotá, around 500 m [41]. This record might represent an isolated population of *D*. *sabanicola*, although we can not confirm that its actual provenance was Tocaima.

**Remarks:** Two dates of publication are cited for the description of *Dasypus sabanicola*. Wetzel and Mondolfi [36], Gardner [38], Wetzel et al. [1] cited 1968, whereas Rincón et al. [54], Feijó and Cordeiro-Estrela [134], Castro [85] referred to 1967. Mondolfi’s description of *D*. *sabanicola* was published in the journal “Memoria” of the Sociedad de Ciencias Naturales La Salle (nowadays called *Memoria de la Fundación La Salle de Ciencias Naturales*), in number 78, tome XXVII, for September to December of 1967. However, the precise date of publication was September 30th, 1968 (stated on the back cover of that volume). In addition, most of the type series (including the holotype) of *Dasypus sabanicola* were collected in January, 1968; and Mondolfi mentioned information about *D*. *sabanicola* hunting provided by Ojasti on March 2th, 1968. Therefore, 1968 is the correct year for the publication of *D*. *sabanicola*.

According to Gibb et al. [10], *D*. *sabanicola* shares a large proportion of its mitogenome (98.7%) with *D*. *mazzai*, the endemic northwestern Argentinean species, leading them to question the validity of species. Indeed, the morphologic similarities between these widely disjunct species are impressive, in agreement with their recent divergence estimate. However, the number of scutes on the posterior border of the scapular shield and number of scutes on the 4^th^ movable band (Fig 7) are diagnostic traits. Such close phylogenetic relationships between species from the Llanos of Venezuela and Colombia and from the Yungas of Argentina seem unique among South American mammals; a detailed paleobiogeographic study is needed to identify possible evolutionary scenarios.

It is noteworthy that the holotype (EBRG 965) of *D*. *sabanicola* has a peculiar roughened pattern of the carapace, in which the large central portion of the osteoderms protrude prominently past the peripheral ones, whereas the paratypes show a smoother pattern. The coloration of the carapace in *D*. *sabanicola* ranges from almost uniformly brownish, with subtly paler flanks (e.g. EBRG 965, EBRG 966, EBRG 964) to a more markedly bicolored pattern with a brownish dorsum and a yellowish band on the flanks (e.g. EBRG 783, EBRG 968). There is also a variation in the angle of the dorsal profile of skull: some specimens exhibit an obtuse angle and hence a more markedly sigmoidal profile (e.g. EBRG 963, EBRG 965, EBRG 968), and others a straighter dorsal profile (e.g. EBRG 964).

One pregnant female (IAVH 534) with four fetuses was collected on 25 March 1972 from Vichada, Colombia.

***Dasypus mazzai* Yepes, 1933**

Figs 28 and 29

**Fig 28:**
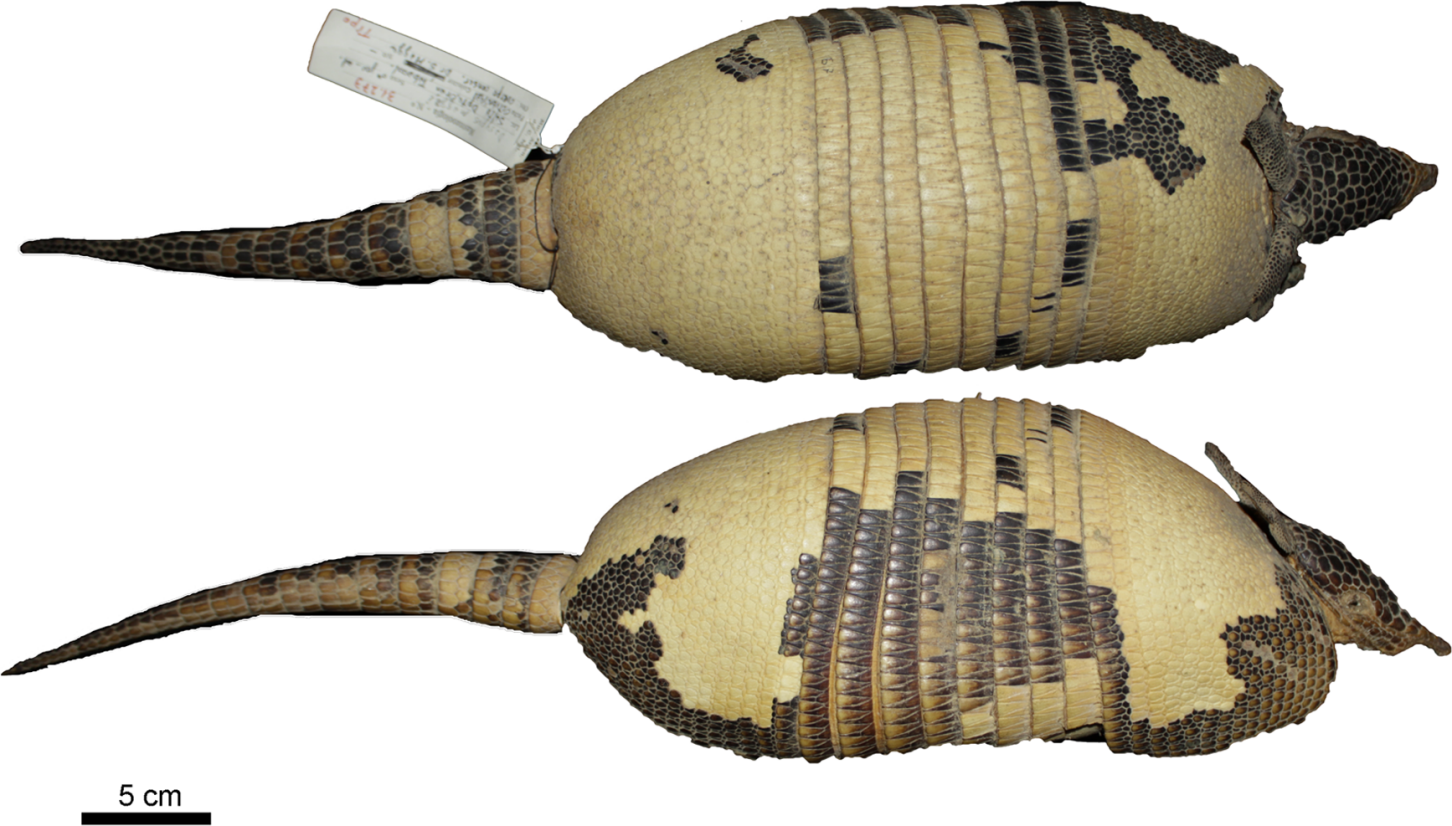
Dorsal and lateral view of the skin of the holotype of *Dasypus mazzai* (MACN 31.273).

**Fig 29:**
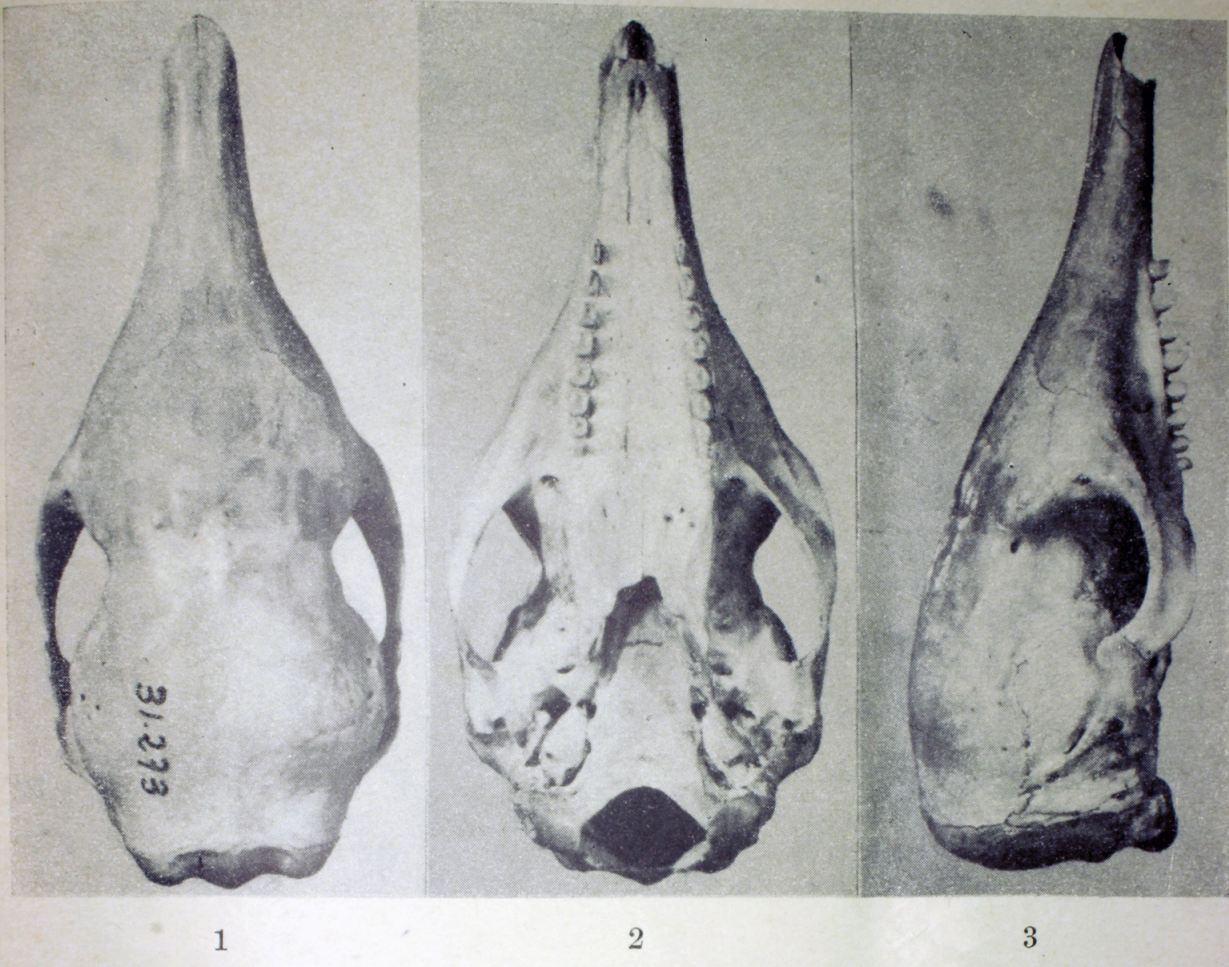
Skull of the holotype of *Dasypus mazzai* (MACN 31.273). Reproduced from Yepes (1933).

*Dasypus mazzai* Yepes, 1933:226; original description.

*Dasypus novemcinctus*: Hamlett, 1939:335; not *Dasypus novemcinctus* Linnaeus, 1758

*Dasypus novemcinctus*: Wetzel and Mondolfi, 1979:55 (part); not *Dasypus novemcinctus* Linnaeus, 1758

*Dasypus hybridus*: Wetzel and Mondolfi, 1979:55 (part); not *Loricatus hybrids* Desmarest, 1804.

*Dasypus (Dasypus) yepesi* Vizcaíno, 1995:7; type locality “San Andrés (1800 msnm), Dto. Orán, Salta, Argentina.”

**Type:** The holotype (MACN 31.273, Figs 28 and 29) designated by Yepes [50] was collected by Salvador Mazza on October 1931. It originally consisted of a carapace with the tail, cephalic shield and ears all attached, skeleton and skull [50]. The paratype is a mounted specimen originally with skull, collected at the same locality as the holotype. Yepes gave no collection number for the paratype, mentioning only the code “32.J.V.Y.”, which likely corresponds to his field number. Wetzel and Mondolfi [36] and Vaccaro and Piantanida [135] referred to the paratype as MACN 13222, and stated that both holotype and paratype skulls are missing. We had access to the paratype of *D*. *mazzai* at the Museo Argentino de Ciencias Naturales (MACN) and agree that the specimen MACN 13222 is the same one figured by Yepes ([50]; Lam. I and II).

**Type locality:** Salta, Tabacal, Departamento Orán, Argentina.

**Diagnosis:** *Dasypus mazzai* is a small-sized species (see Table 6), with eight-nine movable bands, 62–66 scutes along the posterior border of the scapular shield, 57–62 scutes on the 4th movable band, twelve rings on the tail, four digits on the forefoot, and a sigmoidal dorsal profile of the skull.

**Fig 30:**
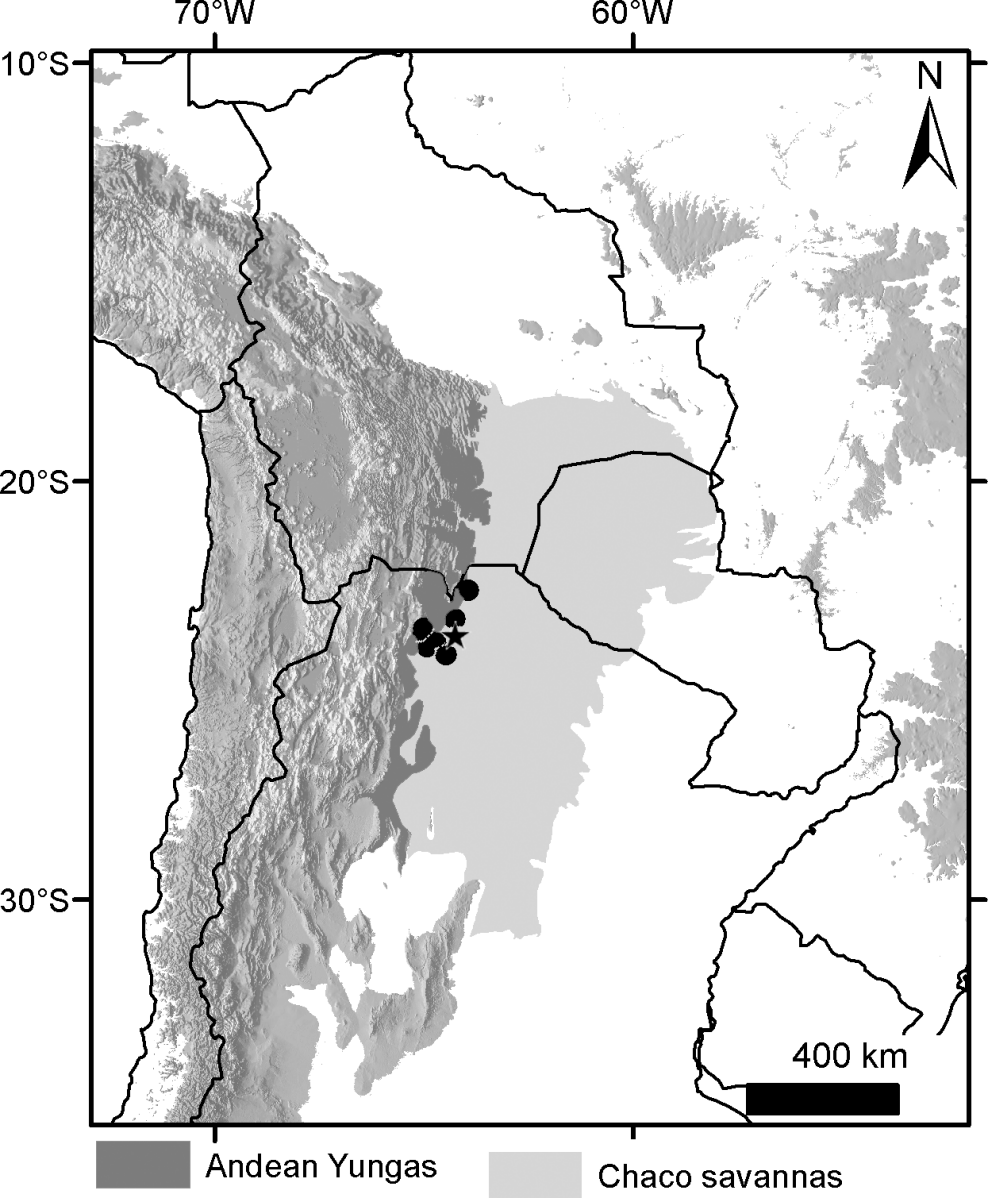
Localities recorded for *Dasypus mazzai* in the Yungas and Chaco of Argentina. Star represents the type locality.

**Distribution:** *Dasypus mazzai* is restricted to two neighboring provinces in northwestern Argentina, Salta and Jujuy (Fig 30). Its habitat includes lower xeric chacoan areas to the montane forest of Andean Yungas [1,37,136]. Despite the absence of records from Bolivia, it is likely that this species also occurs in the southwestern part of the country, as it shares the same habitats found in Salta and Jujuy and there is no apparent geographic barrier.

**Taxonomic history:** Yepes [50] described a new species of *Dasypus* from Salta, northwestern Argentina, based on two specimens (MACN 31.273 and MACN 13222). Shortly afterwards, Hamlett [34] regarded the type series as a composite of two species—he classified the holotype as *Dasypus novemcinctus*, making Yepes’ species a junior synonym of that form and recognized the paratype of *D*. *mazzai* as a new species. Nonetheless, Cabrera [35] treated *D*. *mazzai* as valid species. Wetzel and Mondolfi [36], in keeping with Hamlett, classified the holotype MACN 31.273 as an immature *D*. *novemcinctus* specimen. Their assignment of age was based on the size of the zygomatic arch reported by Yepes [50], because the skull had been lost; they also identified the paratype MACN 13222 as *D*. *hybridus*. Subsequently, Vizcaíno [37] described a new species *Dasypus yepesi* and included the paratype of *D*. *mazzai* in its hypodigm. Interestingly, the characters listed by Hamlett [34] as justifying the paratype as a putative new species were not present in the holotype of *D*. *yepesi*. Nevertheless, Vizcaíno’s species was largely accepted by subsequent authors (e.g. [1,10,38,137]). Recently, Feijó and Cordeiro-Estrela [134] showed that the holotype of *D*. *mazzai* is actually an adult specimen, and the external and cranial measurements of both holotype and paratype are intermediate in size between *D*. *novemcinctus* and *D*. *septemcinctus*. In addition, they showed that the type specimens of *D*. *mazzai* are undistinguishable from *D*. *yepesi*. Hence, *D*. *mazzai* Yepes, 1933 is the senior name for the intermediate-sized species of *Dasypus* from northwestern Argentina and *D*. *yepesi* is its junior synonym.

**Remarks:** The two characters listed by Hamlett [34] that are peculiar to the paratype of *D*. *mazzai* are: “the rounded upper edge of the cephalic shield, with absolutely no indication of a separate occipital lobe,” and “the hexagonal scale-pattern in the central, dorsal third of both scapular and pelvic shields” ([34]; p. 336). However, both traits are occasionally found in other species. For example, *D*. *novemcinctus* specimens from Pinhão, Paraná, Brazil (MHNCI 2997) and from Colombia (ICN 3707) have a similar atypical pattern of the osteoderms on the dorsal shield. In addition, the occipital lobe is completely attached (occipital sulcus absent) occasionally in *D*. *novemcinctus* (EBRG 18, EBRG 10128, MHNLS 4400, AMNH 128136) and *D*. *sabanicola* (MHNLS 5804, MBUCV 5279). Accordingly, those traits highlighted by Hamlett seem to represent unusual individual variation and are not indicative of species rank.

***Dasypus septemcinctus* Linnaeus, 1758**

Figs 31 and 32

**Fig 31:**
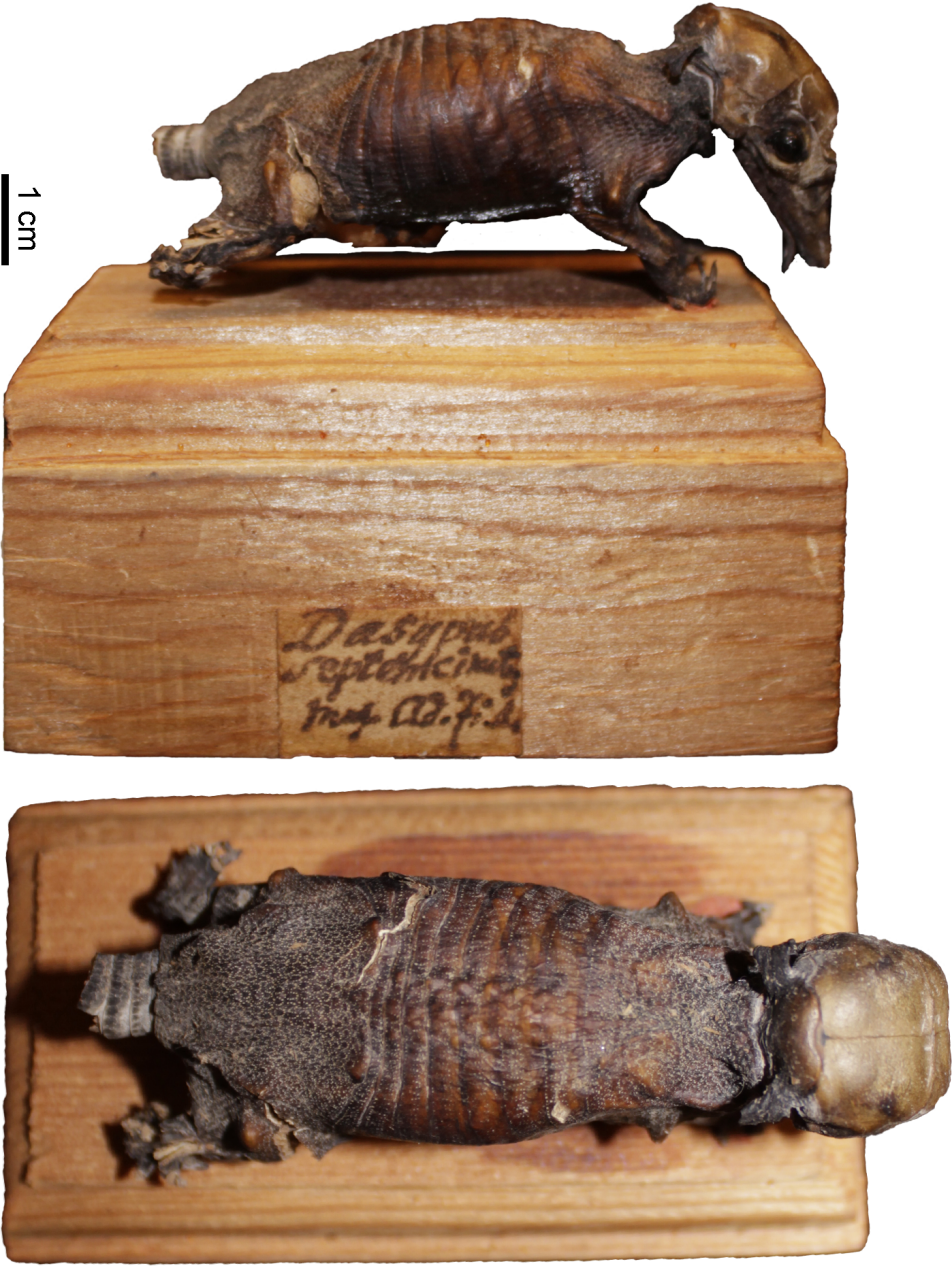
Lateral and dorsal view of the holotype (UPSZTY 24) of *Dasypus septemcinctus* Linnaeus, 1758, showing the label handwritten by C. P. Thunberg, Linnaeus’ pupil and successor as curator of the Uppsala collection.

**Fig 32:**
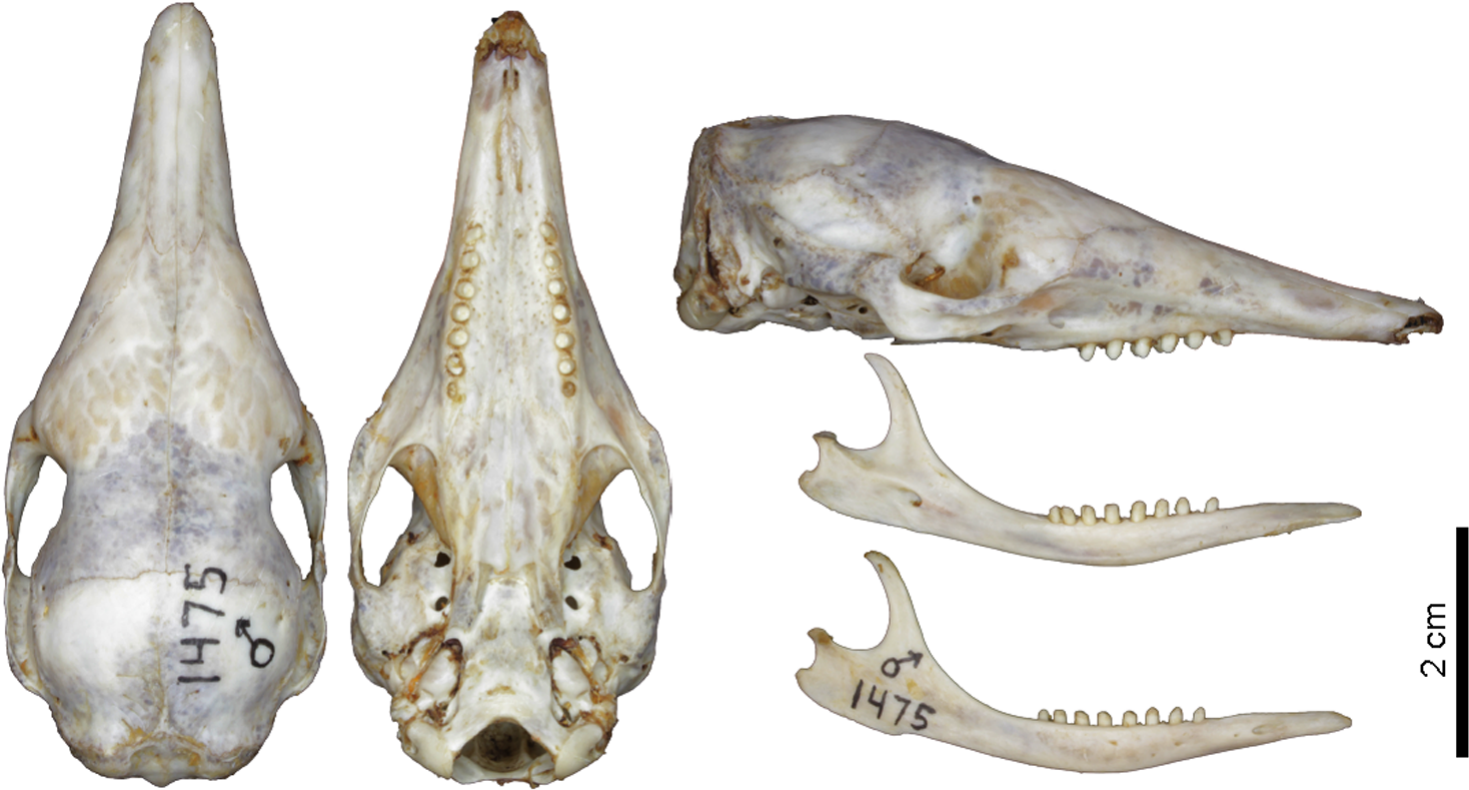
Skull and mandible of *Dasypus septemcinctus septemcinctus* from Brasilia, Brazil (UNB 1475).

**Synonyms**: See under Subspecies

**Type:** In the 10^th^ edition of *Systema Naturae*, Linnaeus ([20]; p. 51) described *Dasypus septemcinctus*, listing only two references: “Amoen[itates]. acad[emicae]. I. p. 281” and “Syst. nat. 6”. The latter refers to the sixth edition of *Systema Naturae*, where Linnaeus ([138]; p. 6) included “Dasypus cingulis septem.” also with two references: “Mus. princ. 4” and “Tauete. Marcgr. bras. 231.” The former refers again to the *Amoenitates Academicae* [139]. In the 10^th^ edition, the latter reference to Marcgrave [108] was listed with *Dasypus novemcinctus* and excluded from *Dasypus septemcinctus*. Thus, the 1758 description of *Dasypus septemcinctus* was based on a single reference, Linnaeus [139]. That work referred to specimens donated to the University Museum in Uppsala by then Crown Prince Adolf Frederik in 1745 [140]. In this dissertation, Balk ([141]; p. 281), disciple of Linnaeus, provided a detailed description of a newborn specimen with seven movable bands (“*Specimen faetus recenter excludum*”) called *Erinaceus loricatus* that certainly was the basis of Linnaeus’ *D*. *septemcinctus*. In the mammal collection of the Museum of Evolution of Uppsala University, there is a neonate mounted armadillo with seven movable bands (UPSZTY 24) with an attached label handwritten by C. P. Thunberg, a pupil of and Linnaeus’ successor as curator of the Uppsala collection (Fig 31), that endorses its XVIII^th^ century age [142]. According to article 73.1.2 of the ICZN [104], “If the nominal species-group taxon is based on a single specimen, either so stated or implied in the original publication, that specimen is the holotype fixed by monotypy.” Therefore, the neonate specimen in Uppsala is the holotype by monotypy of *Dasypus septemcinctus* Linnaeus, 1758 (Fig 31).

**Type locality**: “Habitat in Indiis”. Erxleben ([143]; p. 108) provided an additional description of *Dasypus septemcinctus*, probably based on specimens other than Balk’s [141] neonate, and change the habitat to “Brasilia”. Cabrera [35] proposes Pernambuco, Brazil as type locality, after Hamlett [34]. Feijó and Langguth [144] further restricted to Lagoa Grande, Pernambuco, where the species is known to occur.

**Diagnosis:** *Dasypus septemcinctus* is the smallest species of the genus (mean TL: 453 mm; skull total length: 69 mm; see Tables 5 and 6), with six-seven movable bands, 47–68 scutes along the posterior border of the scapular shield, 43–59 scutes on the 4th movable band, 9–13 rings on the tail, four digits on the forefoot, and a straight dorsal profile of the skull (Fig 32).

**Fig 33:**
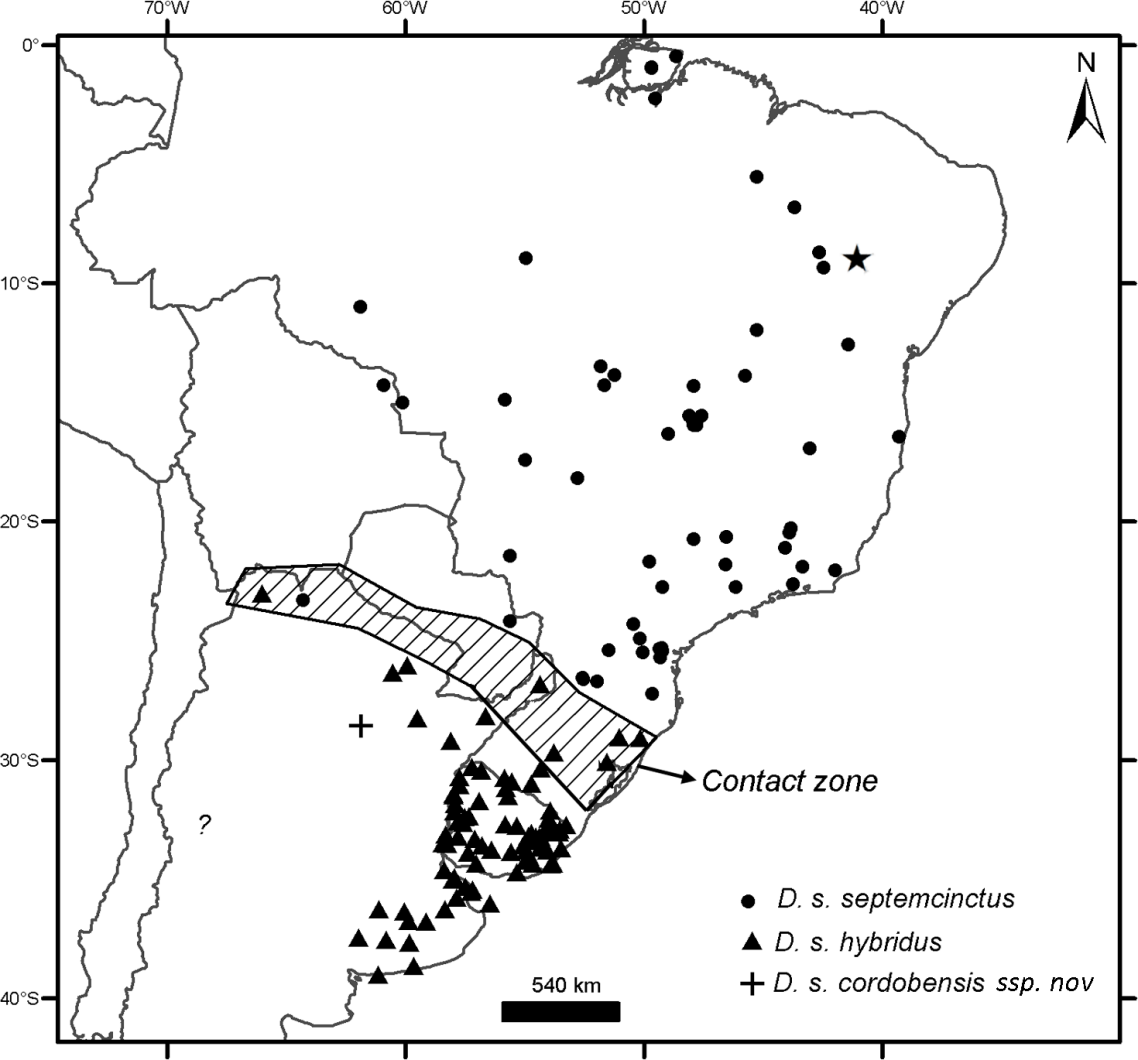
Localities recorded for *Dasypus septemcinctus* Linnaeus, 1758, showing records for the three subspecies and the putative contact zone between *D*. *s*. *septemcinctus* and *D*. *s*. *hybridus*. Star represents the type locality for the nominate subspecies (*D*. *s*. *septemcinctus*). Question mark: doubtful record from Mendoza.

**Distribution:** *Dasypus septemcinctus* has the most southern distribution of the genus, with a latitudinal range from 0° to 39°S. It is distributed in Brazil, eastern Paraguay, Bolivia, Uruguay, and eastern, central and northern Argentina (Fig 33). In Brazil, the records are widely distributed in the central and southern portions of the Atlantic Forest, Cerrado, and Caatinga biomes, as well as in the eastern Amazon, on the right banks of the Lower Amazon and Madeira rivers. The only museum record from Paraguay examined came from a specimen (MNHP 3365) collected at Estancia Roma III, Canindeyu, in the Atlantic Forest biome. The two Bolivian records reported are based on the specimen (MNK 3198) collected in Los Fierros, Noel Kempff Mercado National Park, province of José Miguel de Velazco, Santa Cruz, and on FMNH 119371, collected at San Joaquin, Beni, both collected in savanna formations. Wetzel and Mondolfi [36] reported two additional records from Bolivia: San José de Chiquitos in Santa Cruz, and Villa Montes in Tarija. A single specimen said to come from Mendoza, Argentina (AMNH 40068) represents the westernmost record of the species, but this is likely erroneous, as the weather is extreme for a *Dasypus* species and it has not been recorded in subsequent inventories (see [145,146]). Based on specimens examined, there is also an isolated population from Cordoba, central area of Argentina.

The seven-banded armadillo occurs in a variety of habitats, ranging from evergreen forest to areas with highly seasonal climates, such as Caatinga, Cerrado and Pampas. It is noteworthy that the species is absent from the northern part of the Atlantic Forest and there are few records from the Amazon.

**Taxonomic history:** In the description of *D*. *septemcinctus*, Linnaeus [20] included a comment (“*An sufficienter a fequente distinctus*?”) questioning whether it was different enough from *D*. *novemcinctus* to be assigned to a different species. The only difference apparent to Linnaeus was the number of movable bands. While a number of authors have included both species under the same name (e.g., [147,148]), others have used *D*. *septemcinctus* for the nine-banded armadillo (e.g., [149]). Gray [25] proposed a new generic name, *Muletia*, for *D*. *septemcinctus* Linneaus, separating it from *Tatusia peba* (*= D*. *novemcinctus*) and *Praopus kappleri* (= *D*. *kappleri*). Rhoads [91] considered *D*. *septemcinctus* Linnaeus, 1758 as synonym of *D*. *novemcinctus* and stated that the correct name for the short-tailed armadillo from “tropical America” should be *Dasypus hybridus* Desmarest, 1804. In the same work, Rhoads [91] described *Tatusia (Muletia) propalatum* from Bahia, Brazil, which nowadays is a synonym of *D*. *septemcinctus*. Lönnberg [33] placed *D*. *hybridus* as a synonym of *D*. *septemcinctus*. Conversely, Hamlett [34] provided diagnostic traits to distinguish *D*. *septemcinctus*, *D*. *hybridus*, and *D*. *novemcinctus*, and his view was largely followed by subsequent authors, including Cabrera [35], Mondolfi and Wetzel [36], Wetzel [53], Gardner [38], Wetzel et al. [1], and Abba and Superina [146]. However, Hamlett [34] examined only a limited number of specimens of *D*. *septemcinctus* and hence could not access to the whole variation exhibited by the seven-banded armadillo. Our morphologic results clearly show that the diagnostic traits listed by Hamlett [34] are variable and overlap with those of *D*. *hybridus*. Therefore, we refute the specific rank of *D*. *hybridus*, and consider it as a subspecies of *D*. *septemcinctus*.

**Remarks:** The color of the carapace in *D*. *septemcinctus* is usually uniformly brownish, but it is not uncommon to find specimens that show a yellowish stripe on the sides of the carapace (e.g., MN 10071, MN 59336, MN 2693) which resemble the pattern typically exhibited by *D*. *novemcinctus*. On the other hand, we found specimens of *D*. *novemcinctus* (e.g., MN 41960, MHNCI 3888) that have a predominantly brown carapace that is only slightly paler on the sides.

One specimen (UERJ-CD 121) from Lençois, Chapada Diamantina, Bahia has a peculiar pattern of osteoderms on the dorsal portion of the pelvic shield. They are hexagonal and uniformly flat on the external surface, lacking the typical pattern for the genus. Specimen MHNCI 3552 from Passo Amarelo, Paraná, Brazil possesses a subtle medial crest on the posterior portion of the palatine.

**Subspecies:** Here, we recognize three subspecies of *Dasypus septemcinctus*.

***Dasypus septemcinctus septemcinctus* Linnaeus, 1758**

*[Dasypus] septemcinctus* Linnaeus, 1758:51; original description.

*Cachicama hybridus*: Gervais, 1855:113; not *Loricatus hybridus* Desmarest, 1804.

*Praopus hybridus*: Pelzeln, 1883:99; not *Loricatus hybridus* Desmarest, 1804.

*Tatusia megalolepis* Cope, 1889:134; type locality “Chapada”, Mato Grosso, Brazil.

*Tatusia (Muletia) propalatum* Rhoads, 1894:111; type locality “Bahia”, Brazil.

*[Tatusia (Tatusia)] megalolepis*: Trouessart, 1898:1140; name combination.

*[Tatusia (Muletia)] propalatum*: Trouessart, 1898:1141; name combination.

*Tatu septemcincta*: Thomas, 1900:548; name combination.

*Tatu megalolepis*: Thomas, 1904:243; name combination.

*[Tatus (Tatus)] megalolepis*: Trouessart, 1905:814; name combination.

*[Tatus (Muletia)] propalatus*: Trouessart, 1905:814; name combination.

*Dasypus megalolepe*: Yepes, 1928:468; name combination and incorrect spelling of *Tatusia megalolepis* Cope.

*Dasypus propalatus*: Yepes, 1928:468; name combination.

*Dasypus [(Dasypus)] septemcinctus*: Wetzel and Mondolfi, 1979:53; name combination.

**Type**: UPSZTY 24, newborn mounted specimen (Fig 31).

**Diagnosis:** *Dasypus s*. *septemcinctus* has a less robust skull and is smaller, although it overlaps to varying degrees with *D*. *s*. *hybridus* (Table 8). Therefore, multiple comparisons are required to properly identify these subspecies. The external and carapace measurements with lowest overlap and significant difference in the Tukey's test are the hindfoot length, ear length, length of the scapular and pelvic shield, number of scutes on the posterior border of the scapular shield, and number of scutes on the 3th and 4th movable bands (Table 8). The cranial measurements most useful to differentiate these subspecies are: total skull length, condylobasal length, anterior palatal length, maxilla length, palatal length, infraorbital canal length, nasal length, rostral length, zygomatic breadth, and mandibular lenght (Table 8).

**Table 8:** Diagnostic morphometric traits for the subspecies of *Dasypus septemcinctus*.

	*D*. *s*. *septemcinctus*	*D*. *s*. *hybridus*	*D*. *s*. *cordobensis*
	**External Measurements**		
Hindfoot length*	50.3(30–65) 14	61.1 (40–72) 24	56.2 (48–60) 4
Ear length*^,^†^,^⁺	30(26–35) 12	24.8 (20–30) 23	22 (21–24) 7
**Ratio Total/Tail length**	2.6 (2.3–3.2) 14	2.7 (2.5–3.3) 22	2.82 (2.64–2.9) 6
	**Carapace Measurements**		
Scapular shield length*^,^⁺	68 (54.8–83.2) 23	78.6 (62.5–93) 45	62 (58–65) 7
Pelvic shield length*^,^†^,^⁺	80.4 (67–97) 23	85 (66–101) 47	67.5 (60–77) 7
CaSNR†	62 (44.1–77) 14	56 (36–77) 26	46 (37–54) 6
Scutes on the 3thMB*^,^†	42 (43–57) 22	53 (48–60) 45	52 (51–55) 7
Scutes on the 4thMB*^,^†	48 (43–55) 21	53 (46–59) 45	52 (50–54) 7
SSS*^,^†	52.8 (47–58) 21	57 (51–68) 44	56.4 (53–59) 7
	**Cranial Measurements**		
Greatest length*^,^⁺	65.4 ± 4.6 (57.7–74.9) 24	71.3 ± 3.4 (65.5–81.1) 50	64.7 ± 1.8 (61.7–66.9) 6
Condylobasal length*^,^⁺	60 ± 4.1 (52.2–68.3) 24	65.7 ± 3 (59.9–72.5) 50	59.6 ± 1.19 (57.6–61.1) 6
Anterior palatal length*^,^⁺	14.2 ± 1.4 (11.8–16.9) 24	16 ± 1.3 (12.7–18.7) 50	14.1 ± 0.9 (12.7–15.3) 6
Palatal length*^,^⁺	40.6 ± 5.1 (27.8–52.6) 24	45.6 ± 2.6 (39.6–51.5) 50	40.6 ± 1.5 (38.7–42.8) 6
Maxilla length*	23.9 ± 2.6 (19.4–30.2) 24	25.7 ± 2.2 (21.2–30.7) 50	22.7 ± 1 (21.5–24.1) 6
Infraorbital Canal length*^,^⁺	4.3 ± 1 (2.7–6.3) 24	5 ± 0.8 (3.7–7.2) 50	3.5 ± 0.3 (3.2–3.9) 6
Nasal length*^,^⁺	20.1 ± 2.1 (16–24) 24	22.1 ± 1.4 (19.3–25.5) 50	18.9 ± 2.2 (16.5–21.7) 6
Rostral length*^,^⁺	36.6 ± 3 (31.2–42.5) 24	40.4 ± 2.3 (36–45.6) 50	35.8 ± 1.7 (32.8–37.3) 6
Palatal breadth*^,^†	8.9 ± 1.5 (6.4–11.8) 24	11.3 ± 1.2 (7.5–13.8) 50	10.6 ± 0.4 (9.8–10.9) 6
Zigomatic height*^,^⁺	3.8 ± 0.8 (2.4–5.1) 24	4.5 ± 0.7 (2.7–5.9) 50	4.1 ± 0.4 (3.5–4.7) 6
Mandibular lenght*^,^⁺	50.4 ± 4 (40.7–57.7) 24	55.7 ± 2.7 (49.9–61.2) 50	49 ± 1.7(45.7–50.7) 6
Anterior mandibular length*^,^⁺	12.9 ± 1.5 (8.9–16) 24	15.1 ± 1.2 (12.5–17.6) 50	11.8 ± 1.4 (10.1–13.7) 6
Mandible height*	15.2 ± 1.6 (11.5–18.2) 24	17.8 ± 1.6 (15–22.6) 50	16.5 ± 0.9 (15.1–17.3) 6

Measurements (millimeters) are mean ± standard deviation (minimun–maximun) N.

CaSNR: Dorsal length of the caudal sheath without rings

SSS: number of scutes on the posterior border of the scapular shield

* significant difference (p<0.05) between *D*. *s*. *septemcinctus* vs *D*. *s*. *hybridus* in Tukey's test

† significant difference (p<0.05) between *D*. *s*. *septemcinctus* vs *D*. *s*. *cordobensis* in Tukey's test

⁺ significant difference (p<0.05) between *D*.*s*. *hybridus* vs *D*. *s*. *cordobensis* in Tukey's test

**Fig 34:**
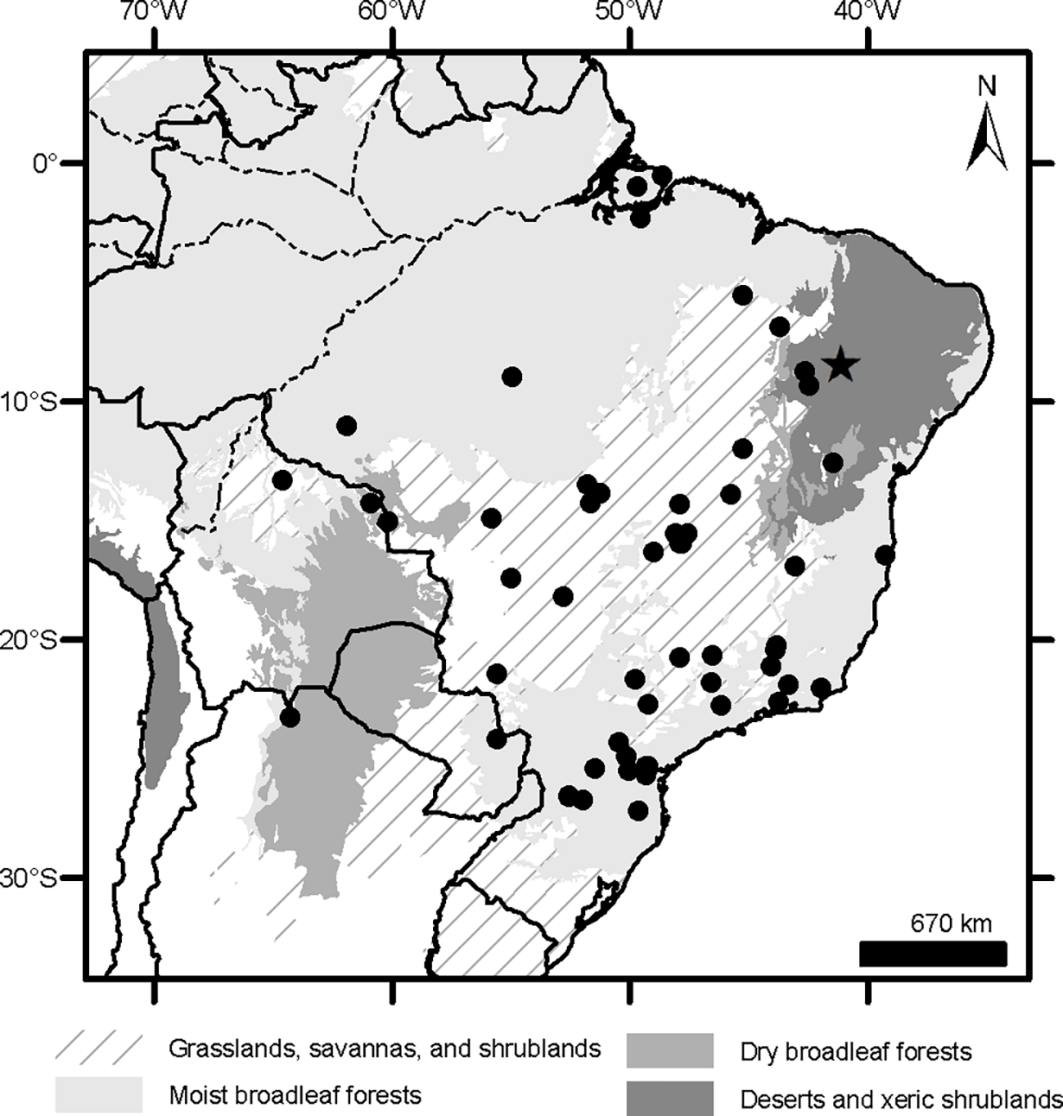
Localities recorded for *Dasypus septemcinctus septemcinctus* Linnaeus, 1758. Star represents the type locality.

**Distribution**: *Dasypus s*. *septemcinctus* is distributed in Brazil, eastern Paraguay, Bolivia, and northern Argentina. In Brazil, the records are widely distributed in the central and southern portion of the Atlantic Forest, Cerrado, and Caatinga biomes, as well as in the eastern Amazon, on the right banks of the Madeira and Lower Amazon rivers (Fig 34).

We examined only a single record from Argentina, consisting of an adult skull (MACN 33.23) from Tabacal, Orán (Salta). However, Wetzel and Mondolfi [36] mentioned other records from La Victoria and Misión Tacaaglé in Formosa. They also reported a record from Pampa del Indio, Chaco (MACN 30.18), but we identified that specimen as *D*. *s*. *hybridus*.

As expected, the southern limits to its distribution are unclear and most likely represent the intergrading contact zone between *D*. *s*. *septemcinctus* and *D*. *s*. *hybridus* (Fig 33). In Brazil, both subspecies occur in the state of Rio Grande do Sul. The southern and central portion of the state are open and covered by grasslands, while the eastern and northern portion are covered by evergreen Atlantic Forest. The visual identification of both subspecies in that state is mainly based on the region (vegetation), which is not a taxonomic character. *Dasypus s*. *septemcinctus* is supposedly restricted to the Atlantic Forest, whereas *D*. *s*. *hybridus* would be limited to open areas. However, it is clear from the present work that *D*. *s*. *septemcinctus* also inhabits southern portions of the Amazon, savannas (Cerrado), and dry forests (Caatinga). Unfortunately, the southernmost well-preserved specimens of this subspecies are from Santa Catarina state (FURB 20101, FURB 18780, FURB 20090; FURB 9109). Nevertheless, it seems very likely that Rio Grande do Sul represents the contact zone between the two subspecies, both in grassland and Atlantic Forest areas.

Besides, other contact zones are probably northern Argentina (Fig 33), where records of both subspecies are known (eg., MACN 251, MACN 33.23, MACN 35148, and MACN 32178). Unfortunately, there are few records based on voucher specimens from the putative contact zone to properly determine the extent of the intergrading areas. Therefore, we strongly encourage further sample efforts along these distribution limit areas in order to assess the morphologic and molecular integrity of *D*. *s*. *septemcinctus* and *D*. *s*. *hybridus*.

***Dasypus septemcinctus hybridus* (Desmarest, 1804)**

Figs 35 and 36

**Fig 35:**
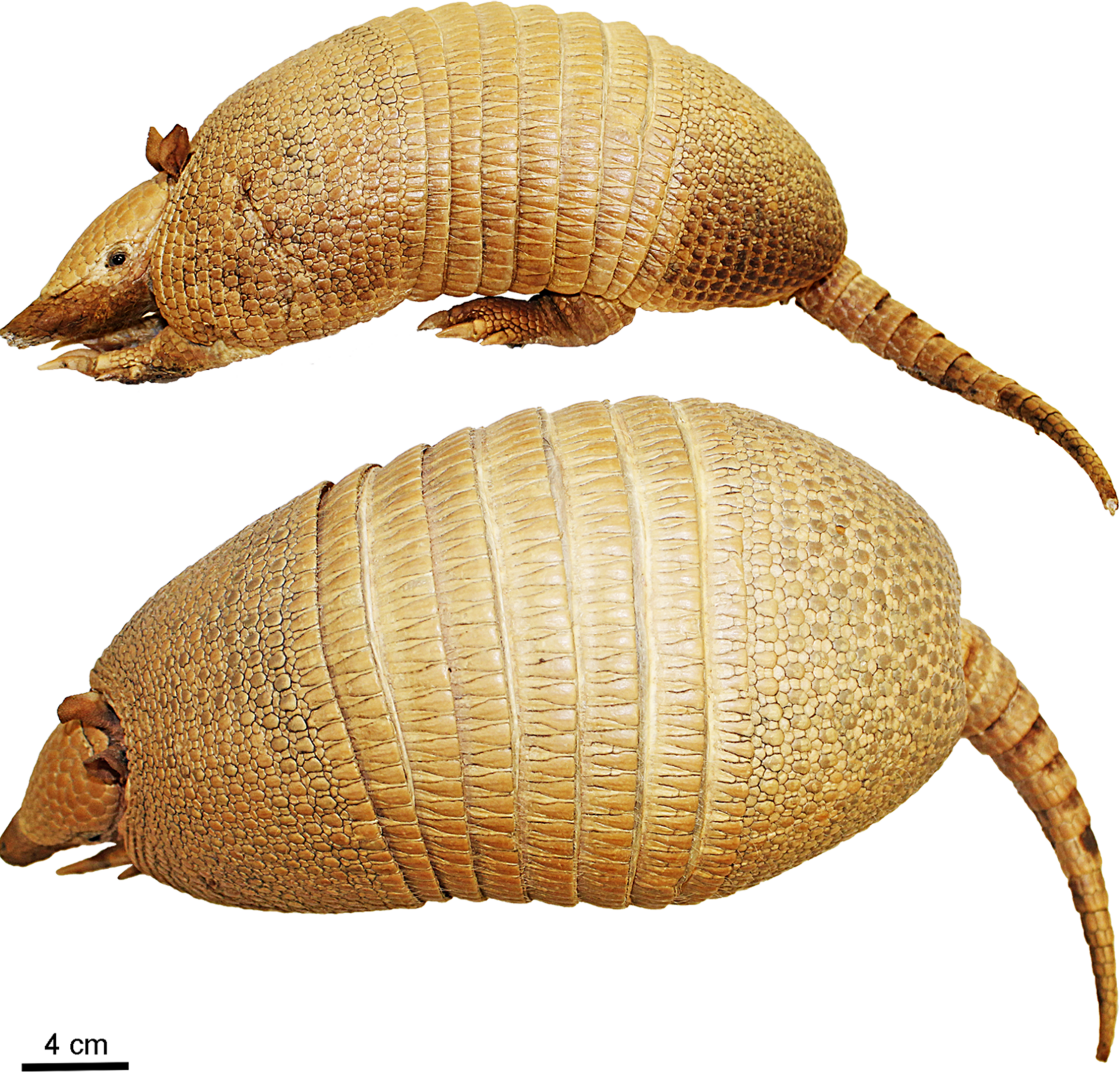
Mounted skin of the neotype (FMNH 29334) of *Loricatus hybridus* Desmarest, 1804 (= *Dasypus septemcinctus hybridus*).

**Fig 36:**
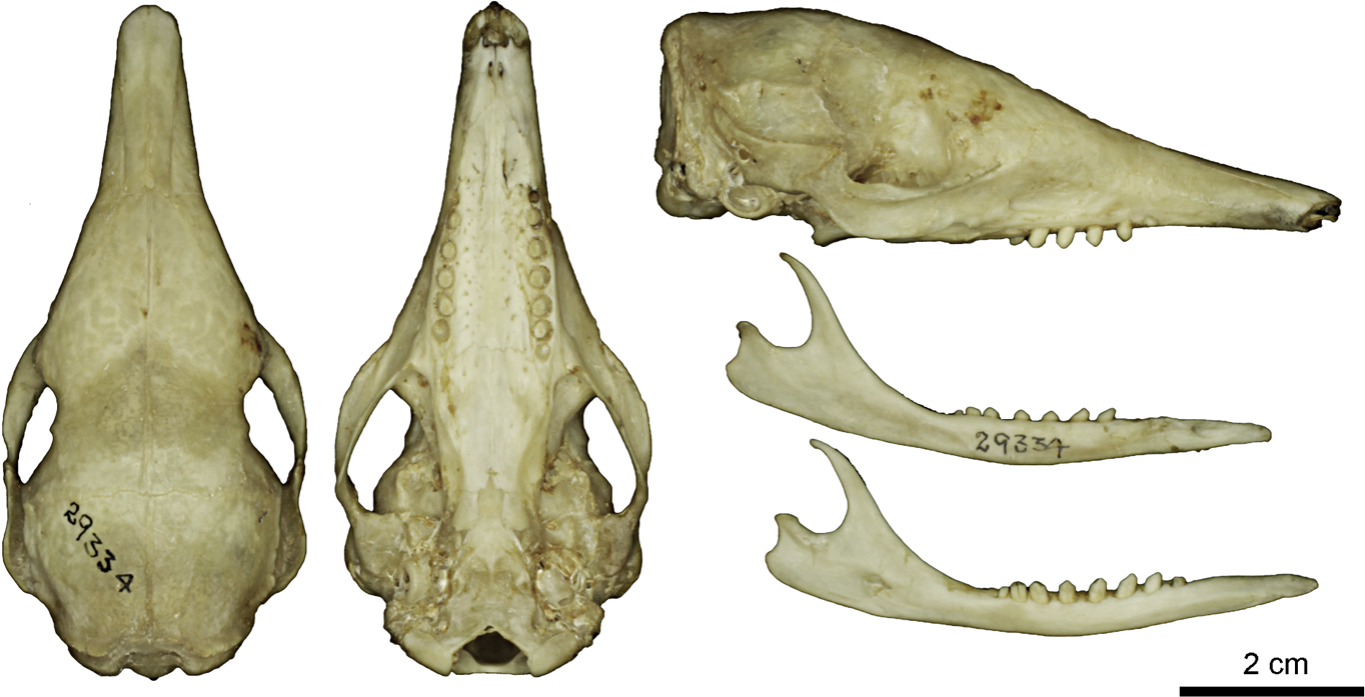
Skull and mandible of neotype (FMNH 29334) of *Loricatus hybridus* Desmarest, 1804 (= *Dasypus septemcinctus hybridus*).

*lor[icatus]*. *hybridus* Desmarest, 1804:28; original description.

*[Dasypus] hybridus*: Fischer, 1814:126, name combination.

*[Dasypus] auritus* Illiger, 1815:108; nomen nudum.

*T[atus]*. *auritus* Olfers, 1818:221; type locality “Paraguay”; based solely on “*T*. *mulet*” of Azara [150].

*Tatusia hybridus*: Lesson, 1827:311; name combination.

*Dasypus hybridus*: Martin, 1837: 13; name combination.

*T[atusia] hybrida*: Turner, 1853:213; corrected gender concordance.

*Praopus hybridus*: Burmeister, 1861:428; name combination.

*Muletia septemcincta*: Gray, 1874:246; name combination.

*Tatu hybridus*: Lahille, 1899:203; name combination.

*[Tatusia (Muletia)] hybrida*: Trouessart, 1898:1140; name combination.

*[Tatus (Muletia)] hybridus*: Trouessart, 1905:814; name combination.

*Muletia hybrida*: Miranda-Ribeiro, 1914:46; name combination.

*D[asypus]*. *Brevi-cauda* Larrañaga, 1923:344; type locality not given, but Uruguay implied (p.242); based on Azara’s ([105]; p. 156) “*Mulita”*.

*Dasypus hibridus*: Azevedo, El Achkar, Martins, and Ximénez, 1982:95; incorrect subsequent spelling of *Loricatus hybridus* Desmarest, 1804.

**Type:** Desmarest [148] named “*Le tatou mulet de*[*sic*] *d’Azara*” ([150]; p. 186) as *Loricatus hybridus*. The tatou mulet of Azara refers to a small-sized armadillo (46 cm), with smaller legs than “*tatou noir*” (= *D*. *novemcinctus*), tail much shorter than “tatou noir,” with six to seven movable bands (fetuses with 5 bands) ([150]; p. 186–191). Although a few specimens from Azara’s expedition are still preserved [35,151,152], we are unaware of any remaining armadillo, and assume that none exists, leaving the species without any type material.

*Loricatus hybridus* figures prominently in taxonomic controversies [33,34,81]. Schreber [153] believed that the "tatou mulet" of Azara was the same as Linnaeus’s [20] *D*. *septemcinctus*. On the other hand, Desmarest [147] believed that Azara’s armadillo was a new species and named it *Loricatus hybridus* in 1804. Notwithstanding, Gray ([26]; p. 246) employed the name *D*. *septemcinctus* for Azara’s *“tatou mulet*”, a course followed by Lönnberg [33] and Sanborn [154], who both listed *D*. *hybridus* as a synonym of *D*. *septemcinctus*. Rhoads [91] argued that *D*. *septemcinctus* Linnaeus, 1758 refers to an unrecognizable and composite species, and that the first unambiguous name for the short-tailed armadillo with six to seven bands is *Dasypus hybridus* (Desmarest, 1804).

Hamlett [34] examined specimens from Brazil, Argentina and Uruguay and was the first to list diagnostic traits to distinguish *D*. *septemcinctus* and *D*. *hybridus*, mainly based on differences in the size of the ear and tail. Species separation on this basis was rejected by Frechkopf and Yepes [155] and Moeller [156], but followed by Cabrera [35], Wetzel and Mondolfi [36], and Wetzel [53]. Interestingly, Wetzel and Mondolfi [36] found the ratio of tail length to body length similar in these species, but agreed with Hamlett that *D*. *septemcinctus* has larger ears than *D*. *hybridus*. Later, Wetzel [53] showed substantial overlap between species. Wetzel et al. [1] used the length of the body and ear and the number of scutes on the fourth movable band to separate these species.

As documented here, the two taxa show extensive overlap in both external and cranial measurements which lead us to recognize them as conspecifics. Accordingly, Gibb et al. [10] found their mitogenomes were almost identical, with 99.3% similarity between mtDNA of specimens they named *D*. *septemcinctus* and *D*. *hybridus*.

Therefore, considering (a) a long and confused taxonomic history; (b) the great morphological and genetic similarity between taxa; (c) the holotype of *Dasypus s*. *septemcinctus* lacks important diagnostic features; and (d) no specimen of *L*. *hybridus* collected by Azara is known, we find it justified to designate a neotype with precise locality in order to clarify the taxonomy of *Loricatus hybridus* Desmarest, 1804 (ICZN [104]: Art 75).

The neotype was selected based on the integrity of the material and taking into account the original distribution of the species as reported by Azara. He stated that “nunca he encontrado Mulitas [*D*. *s*. *hybridus*] al Norte de los 26 1/2 grados, desde donde se extiende hasta mas [*sic*] allá de los 36,” ([105]; p. 156), and, according to Cabrera [35], this is the original type locality of the species.

The neotype is an adult male collected at Estancia Jeffries, eight miles East of Treinta y Tres, Department of Treinta y Tres, Uruguay on December 1^st^, 1926 by Colin Campbell Sanborn (collector number 1368), and deposited in the Field Museum of Natural History, with the number FMNH 29334. It consists of a mounted skin with cleaned skull and mandible (Figs 35 and 36). The neotype has seven movable bands at the midline of the dorsum and eight along the sides of the body; four digits on the forefoot and five on the hindfoot; 57 scutes at the posterior margin of the scapular shield; 52 scutes on the 3^rd^ movable band; 51 on the 4^th^ movable band; and eleven concentric rings of scutes on the tail, covering 79% of the tail.

External measurements (in mm) are: total length 479; tail length 176; hindfoot with claw 67, ear length (taken from the mounted specimen) 16.62. Carapace measurements (in mm) are: cephalic shield length 58.6; scapular shield length 79; pelvic shield length 79; ringed tail length 138. Cranial measurements (in mm) are: total length 68.22; condylobasal length 62.63; anterior palatal length 14.37; palatal length 42.77; maxilla length 24.47; palatine length 13.8; infraorbital canal length 4.15; maxillary toothrow length 15.73; nasal length 20.73; lacrimal length 5.52; rostral length 38.53; anteorbital breadth 22.84; tooth length 1.36; palatal breadth 10.69; palatine breadth 11.03; postorbital constriction 18.06; braincase breadth 25.67; zygomatic breadth 31.08; mastoid breadth 22.04; height of jugal bone 4.91; mandible length 52.82; anterior mandibular length 12.92; mandibular toothrow length 18.03; height of mandible 15.78.

**Type locality**. Estancia Jeffries, eight miles [13 km] East of Treinta y Tres, Departament Treinta y Tres, Uruguay [33° 13’S, 54° 32’ W]. According to Sanborn ([154]; p. 149): “This locality, the Estancia Jeffries, was made up of gently rolling hills given over to sheep and cattle raising. It was cut by numerous streams and swamps, but woods, aside from eucalyptus groves, were practically absent.” Paynter ([157]; p. 27) cited this locality as Río Olimar Grande, about eight miles [13 km] East of Treinta y Tres, Uruguay.

**Diagnosis:** *Dasypus septemcinctus hybridus* is small (mean TL: 462 mm; skull total length 71.27 mm), with 6–7 movable bands, 51–68 scutes along the posterior border of the scapular shield, 48–60 scutes on the 3^rd^ movable band; and 46–59 scutes on the 4^th^ movable band, nine-twelve rings on the tail. A detailed comparison with *D*. *s*. *septemcinctus* is provided in the topic “Diagnosis” of the previous subspecies (see Table 8).

**Fig 37:**
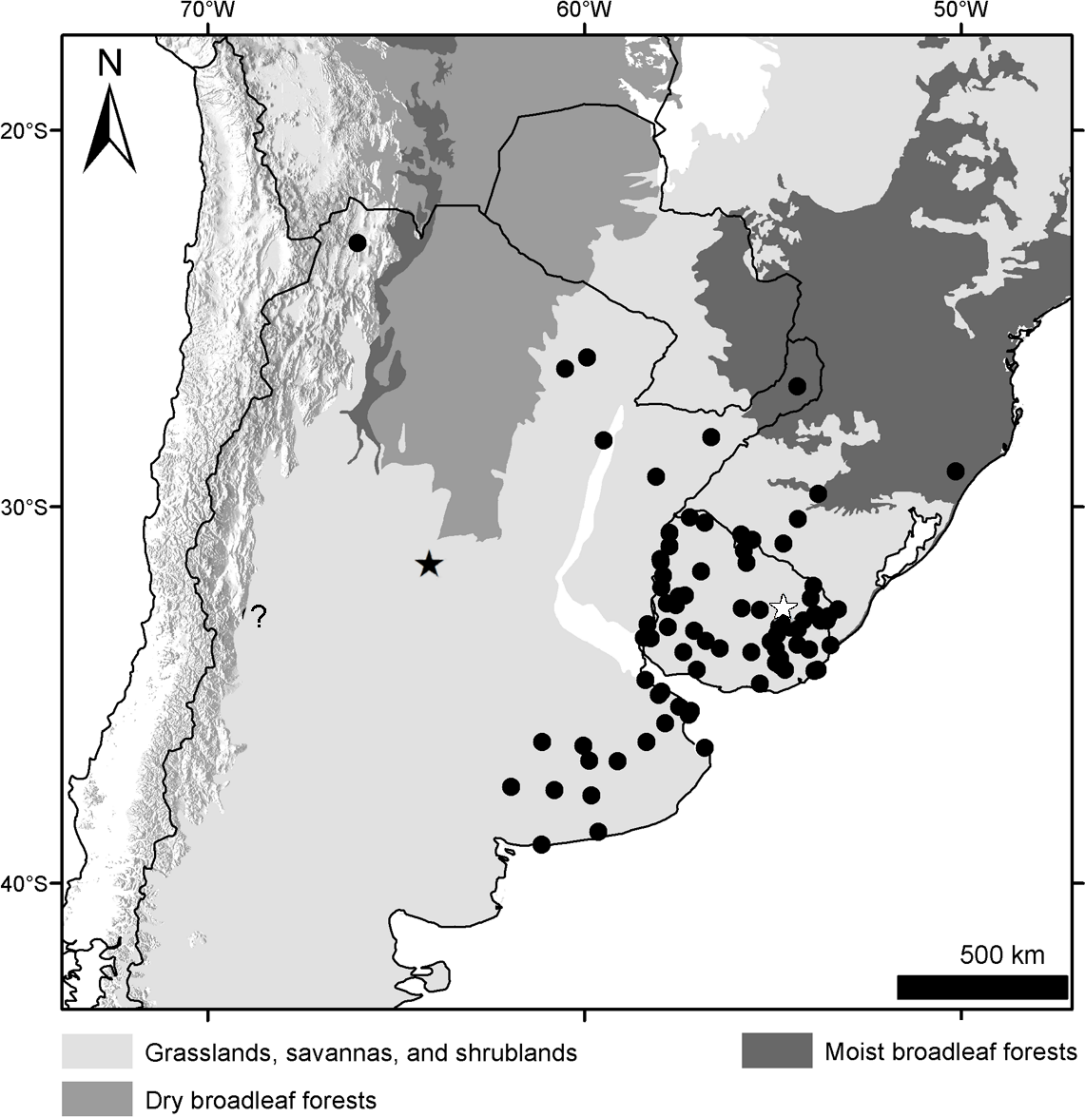
Localities recorded for *Dasypus septemcinctus hybridus* (dots) and *D*. *s*. *cordobensis* (star). Question mark: doubtful record from Mendoza. White star represents the type locality of *D*. *s*. *hybridus*.

**Distribution:** *Dasypus s*. *hybridus* is distributed from 23°S to 39°S (Fig 37). It occurs in southern Brazil, Uruguay, and eastern, central and northern Argentina. Although we could not find any specimens from Paraguay, Azara [150] stated that the *mulita* is found from Misiones and Ñeembucú, Paraguay southward to the Pampas of Buenos Aires. Wetzel and Mondolfi [36] reported two additional records from eastern Paraguay: Villarrica in Guairá and Curupayty in Misiones.

As with *D*. *s*. *septemcinctus*, the northern limits of *D*. *s*. *hybridus*’s distribution are not well understood and may represent an intergradation zone with *D*. *s*. *septemcinctus*. The northernmost records (MACN 35148 and MACN 32178) come from Jujuy and Salta, northwestern Argentina, where *D*. *s*. *septemcinctus*, *D*. *mazzai* and *D*. *novemcinctus* also occur. At the other extreme, in the northeastern portion of the distribution, there is a *D*. *s*. *hybridus* record from Cambará do Sul (MCN 3377), an Atlantic Forest region in the northern portion of Rio Grande do Sul. This record contradicts the impression that *D*. *s*. *hybridus* is restricted to the grasslands areas of Rio Grande do Sul. Other records of *D*. *s*. *hybridus* in that state are from Pinheiro, Santa Maria (MPEG 22218), Dom Pedrito (MPEG 22199), and São Gabriel (MCN 2205). Two other specimens are incomplete and could not be differentiated from *D*. *s*. *septemcinctus*; these are from Caxias do Sul (MCNU 2390) and Eldorado do Sul (MCNU 2503).

**Remarks:** The etymology of the epithet *hybridus* is curious and has led to some misinterpretations. Desmarest ([148]; p. 28) named “*le tatou mulet de d’Azara*” as *Loricatus hybridus*. Azara’s name was based on the indigenous term *Tatou m’bouriqua* used by the Guaranís (natives from Paraguay) and means mule armadillo (or “Tatou mulet” in French). According to Azara, this name derived from the fact that this species has large, straight, and parallel ears resembling a mule. The mule is a hybrid between a female horse and a male donkey, and most likely was the inspiration for Desmarest’s naming of the species. Another interpretation, one suggested by Braun and Mares [158], is that *hybridus* denoted the species being a hybrid between *D*. *novemcinctus* and *D*. *septemcinctus*, but this is unlikely because Desmarest treated them both as synonyms.

It is noteworthy that some specimens of *D*. *s*. *hybridus* (e.g., MNHN 2675, MNHN 2771, AMNH 205691) exhibit a small but remarkable postorbital process, absent in other *Dasypus* taxa.

***Dasypus septemcinctus cordobensis* new subspecies**

urn:lsid:zoobank.org:act:AB12393C-1F2A-47D8-92AF-D5E9CD777951

Fig 38

**Fig 38:**
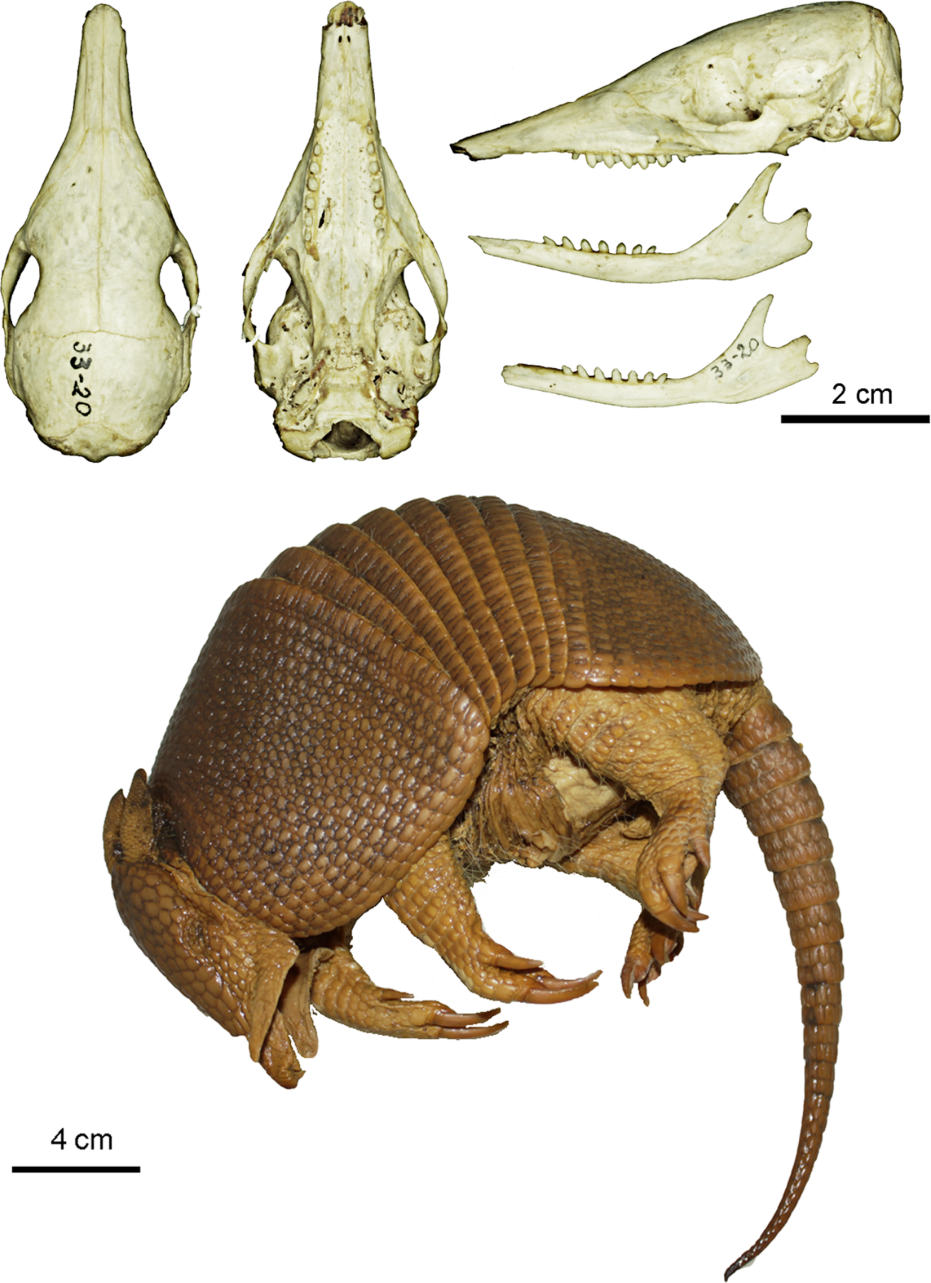
Skin, skull, and mandible of the holotype of *Dasypus septemcinctus cordobensis* new subspecies (MACN 33.20).

**Holotype**: MACN 33.20, adult male, specimen in alcohol with separated skull and mandible collected in “Cordoba, Argentina” by Dr. Enrique A. Artayeta on 1933. The holotype has six movable bands at the midline of the dorsum and seven on the sides of the body; four digits on the forefoot and five on the hindfoot; 56 scutes along the posterior margin of the scapular shield; 52 scutes on the 3^rd^ movable band; 54 on the 4^th^ movable band; and twelve concentric rings of scutes on the tail, covering 76% of the tail. External measurements (taken from the preserved alcohol specimen, in mm) are: total length 475; tail length 160; hindfoot with claw 60, ear length 24.

**Paratypes**: Four adult males (MACN 33.10, MACN 33.14, MACN 33.16, MACN 33.19), five adult females (MACN 33.11, MACN 33.12, MACN 33.15, MACN 33.17, MACN 33.18), and one subadult male (MACN 33.13). All are alcohol-preserved specimens collected by Dr. Artayeta in Cordoba, Argentina.

**Fig 39:**
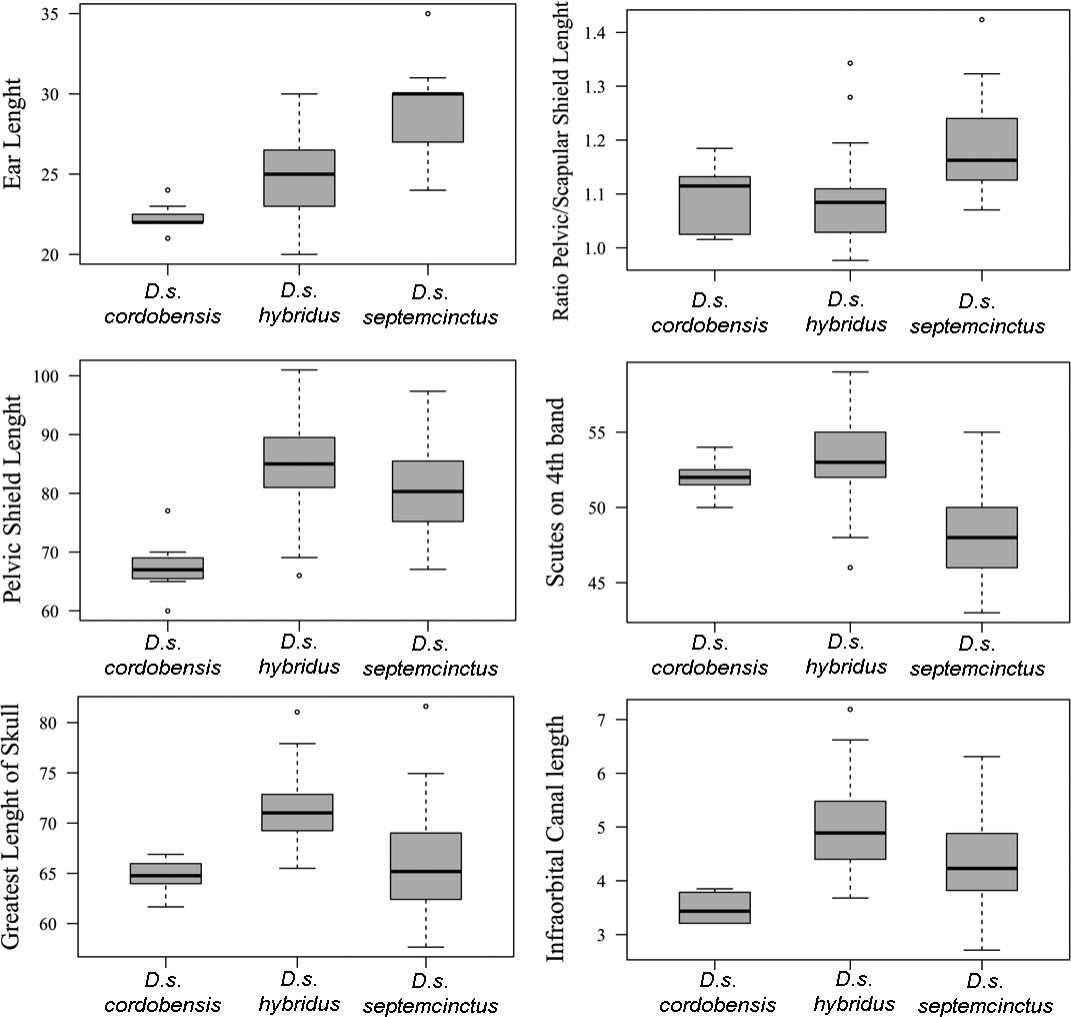
Boxplots of selected external, carapace and cranial diagnostic measurements between *Dasypus septemcinctus cordobensis*, *D*. *s*. *hybridus*, and *D*. *s*. *septemcinctus*. Boxes delimit de 1^st^ and 3^rd^ quantiles, the heavy midline the median and whiskers correspond to respective quantiles -/+ 1.5 IQR. Outliers are represented by circles.

**Diagnosis**: *Dasypus s*. *cordobensis* is distinguished from *Dasypus s*. *septemcinctus* and *Dasypus s*. *hybridus* based on external, carapace and cranial measurements (Table 8). The measurements with lowest overlap and significant difference in the Tukey's test between *D*. *s*. *cordobensis* and *D*. *s*. *septemcinctus* are: ear length, length of pelvic shield, length of the caudal sheath without rings, number of scutes on the 3th and 4th movable bands, number of scutes on the posterior border of the scapular shield, and palatal breath (Table 8). Comparing to *D*. *s*. *hybridus*, *D*. *s*. *cordobensis* can be distinguised based on: ear length, length of scapular and pelvic shields, total lenght of the skull, condylobasal length, anterior palatal lenght, infraorbital canal length, nasal length, rostral length, zigomatic length, mandibula lenght, and anterior mandibular length (see Table 8). For a graphic comparison of the measurements between *D*. *s*. *corbodensis* and the other two subspecies see Fig 39.

**Distribution**: The scant information about the distribution of *D*. *s*. *cordobensis* is limited to “Norte de Cordoba, Argentina,” according to the MACN museum catalogue (Sergio Lucero, pers. comm.). Cordoba is the name of the Argentinian province and also the capital of that province, situated in the central part of the country. Eastward, the province is a flat grassland area, while its western part is mountainous and covered by xerophytic woodland and grassland, ranging in altitude from 100 m up to 2400 m. Five ecoregions are present in Cordoba: Pampa to the extreme east, Espinal in its center, Dry Chaco in the north and west, and Sierra Chaco and Upper mountain grassland in the west [159].

Records of this species in Cordoba are scarce and, according to Soibelzon et al. [159], are limited to lower grasslands in the southern part of the province; it is not found in the mountains. The catalogue reference to northern Cordoba may indicate an isolated population living in a non-grassland area. Nevertheless, further discussion requires the collection of new samples to clarify the distribution range and habitat of this new subspecies.

## Conclusion

This work represents a comprehensive overview of the systematics of the long-nosed armadillos, compiling information about their taxonomy, distribution, geographic variation, species history, and ecology. Based on the largest sample of the genus thus far examined, we recognize eight species of *Dasypus*: *D*. *beniensis*, *D*. *kappleri*, *D*. *mazzai*, *D*. *novemcinctus*, *D*. *pastasae*, *D*. *pilosus*, *D*. *sabanicola*, and *D*. *septemcinctus*. We end the 200-year debate about the validity of the southern long-nosed armadillo, *Dasypus hybridus*, by considering it as a subspecies of *Dasypus septemcinctus*. Through the designation of a lectotype for *Dasypus novemcinctus* Linnaeus and a neotype for *Loricatus hybridus* (= *D*. *septemcinctus hybridus*) Desmarest, each taxon has now a name-bearing type specimen that is crucial for any further taxonomic study.

Despite our extensive sampling, some species remain imperfectly known and characterized. The absence of specimens of *Dasypus beniensis* and *D*. *pastasae* from central Amazonia prevents robust delimitations of their distribution limits. For instance, only a single incomplete specimen is available in northwestern Brazil, between the Madeira and Negro rivers, to document the eastern and northern limits of *D*. *pastasae* and *D*. *beniensis*’ distributions, respectively ([3], Figs 11 and 13). Another example is the contact zone between *D*. *s*. *septemcinctus* and *D*. *s*. *hybridus* (Fig 33), where the few specimens available preclude detailed analyses regard the morphological integration of these taxa and the extent of their integradation zone. The central region of Argentina, habitat for the new subspecies *D*. *s*. *cordobensis*, also represents an important sampling gap.

## Supporting information

S1 Appendix List of specimens of *Dasypus* examined here and their collection localities. (DOCX)

S2 Appendix External and cranial qualitative characters used in this study to assess the morphological variation in *Dasypus*. Characters were selected based on our direct observation of specimens in museums and on their use as diagnostic traits in previous taxonomic works on Cingulata [see references 3,6,24,26,31,33,34,36,48,49,50,51,52,53,54,55 in the main paper].
(DOCX)

S3 Appendix Definition of landmarks on the dorsal, ventral and lateral views of the skull used in this study. See Fig 2 in the main paper.
(DOCX)
