# Supplementary material for: Taxonomic revision of the long-nosed armadillos, Genus Dasypus Linnaeus, 1758 (Mammalia, Cingulata)
Source: PLoS One. 2018 Apr 6;13(4):e0195084. doi: 10.1371/journal.pone.0195084 (PMC5889077; doi:10.1371/journal.pone.0195084)
Supplement: S1 Appendix — (DOCX) [file pone.0195084.s001.docx]

**Supporting information**

**S1 Appendix**  – List of specimens of *Dasypus* examined here and their collection localities.

***Dasypus beniensis* Lönnberg, 1942**

**BOLIVIA**: ***Beni***: Bella Vista, provincia Itenez (MNK sn); Confluencia de los rios Madre de Dios y Beni, Victoria, Bolivia (NRM 583386). **BRAZIL**: ***Pará*:** Saude, margem esquerda do rio Tocantins, 170km S de Tucurui, Pará (MPEG 12331); Taperinha, Santarém, Pará (MPEG 4676, MPEG 4678); Tavio, Rio Tapajós (MZUSP 19973, MZUSP 19974); Transamazônica km 212, Jacareacanga, Itaituba (MPEG 8481).

***Dasypus pastasae* Thomas, 1901**

**BRAZIL**: ***Amazonas*:** Rescujubim, Município Jutaí (MPEG 47762). **COLOMBIA**: ***Meta*:** Los Micos, San Juan de Arama (FMNH 87914, FMNH 87919); Extremo Sur de la Sierra de la Macarena, margen izquierda del Rio Guayabero, frente al Caño Lozada (ICN 1621, ICN 1622); Municipio de Mesetas, vereda San Isidro, cuenca media rio Duda (ICN 11625); Rio Guapaya, La Macarena, Meta (FMNH 87917, FMNH 87916, FMNH 87915, FMNH 87918); San Juan de Arama, Meta (FMNH 88469); Villavicencio (AMNH 136251); Exact locality unknown (AMNH 136253, AMNH 136255). ***Nariño-Putumayo***: Cuenca alta de los rio Rumiyaco-Rancheria (2°28' 6"N; 77° 17' 8"W) (IAVH 6858). **ECUADOR**: ***Napo*:** Avila Viejo, Loreto (QCAZ 3370, QCAZ 3376). ***Orellana*:** San José, Canton Loreto (0° 30' S, 77° 19' W) (MEPN 8496); Exact locality unknown (NMW St 1018). ***Sucumbios*:** Nueva Loja, Lago Agrio (00°5'30" N, 76°52' W) (MEPN 7654). **PERU**: ***Loreto*:** Campamento El Fuerte (Cerros de Orellana), Distrito Vargas Guerra, prov. Ucayali (MUSM 697); Caruchupa (AMNH 98812); Iquitos (AMNH 98464); Requena, Rio Galvez, San Juan, Distrito Yaquerana, prov. Maynas (MUSM 11081, MUSM 11083); San Juan, Galvez River (AMNH 268227, AMNH 268228). ***Madre de Dios*:** CICRA Los Amigos, Distrito Manu, prov. Manu (MUSM 23073). ***Pasco*:** Exact locality unknown (FMNH 30348, FMNH 30349, FMNH 30350). ***Roque*:** Exact locality unknown (NRM 631235, NRM 586560). ***Ucayali*:** Lagarto (AMNH 76573, AMNH 76574). **VENEZUELA**: ***Amazonas*:** Mount Duida, Tamatama, Atabapo (AMNH 76933); Ocamo, Rio Ocairo, Alto Orinoco? (MHNLS 3593); San Juan de Manapiare (MHNLS 608). ***Bolivar***: 19 km NE Icabaru, Uaiparu (EBRG 3305); Rio Ichum, afluente margen Izquierda rio Paragua, abajo del Salto Ichuno Espuma (4°46'7"N; 63°21'16"O) (EBRG 25559).

***Dasypus kappleri* Krauss, 1862**

**BRAZIL**: ***Amapá***: Rio Tracajatuba (MN 20581). ***Amazonas*:** Apuaú, Manaus (MPEG 7128); Balbina (MZUSP 24798); Estrada Manaus-Itacoatiara, km 46, Manaus (MPEG 7127); Manaus (MPEG 8907); Rio Uatumã, margem direita, jusante barragem, ~ 1.5km, Balbina (INPA 303); Rio Uatumã, Usina hidrelétrica de Balbina (MPEG 22130, MPEG 22131, MN 26917, MN 26931). ***Pará*:** As pedras, Rio Cuminá-Miri (MZUSP 19967); Km 93, Rodovia BR 14, Capim (MZUSP 8950); Rio Saracazinho, Porto Trombetas (MPEG 12591). ***Rondônia*:** Garimpo Bom Futuro, Arequemes (MN 42853, MN 42854). **FRENCH GUIANA**: near Sinnamary, Paracou (AMNH 267011). **GUYANA**: Kalacoon (AMNH 48132); Kartabo (AMNH 48131, AMNH 48222, AMNH 64118, AMNH 64119); Exact locality unknown (ZMB_Mam_3174). **SURINAME**: Saramacca river, Loksie Hatti, Brokopondo (FMNH 95451); Exact locality unknown (SMNS 285, SMNS 26642, ZMB_Mam_6162, ZMB_Mam_6168, ZMB_Mam_85892). **VENEZUELA**: ***Bolivar*:** 56 km SE El Manteco (EBRG 3180); Amansaguapo via el Paraiso upata (EBRG 1417); El Carapo, La Tigra, el Palmar (EBRG 1691); Reserva Florestal Imataca, alt. 180 m, Unidade V. (EBRG 17486); Reserva Florestal Imataca, alt. 180 m, Unidade V., PEA, Sector N. (EBRG 17555); Rio Grande, El Palmar (EBRG 1490, EBRG 1491); Rio Grande (EBRG 1415, EBRG 1416, EBRG 231, EBRG 570, EBRG 582, EBRG 604) .

***Dasypus novemcinctus* Linnaeus, 1758**

**ARGENTINA**: ***Chaco*:** Las Palmas y El Perdido (MACN 39458, MACN 39457, MACN 39438, MACN 34459, MACN 39465, MACN 39461); Exact locality unknown (MACN 39459, MACN 39458, MACN s/n1, MACN 49397, MACN 49383). ***Entre Rios*:** Villaguay, Sauce de Luna (MACN 13221). ***Estero*:** Exact locality unknown (MACN 36962, MACN 36.966). ***Formosa*:** Exact locality unknown (MACN 29546). ***Misiones*:** Puerto Denis (MACN 21.1); Santa Ana (MACN 3392); Exact locality unknown (MACN 49391). ***“Patagonien”***: exact locality unknow (NMW 64561, NMW 64562). ***Santa Fé*:** (MACN 42106, MACN s/n2); Chaco santafecino, Vera Calchaqui (MACN 3016). ***Santiago del Jujuy*:** (MACN 34590). **BELIZE:** Manatee, Gales Point (AMNH 146591); Silk Grass (FMNH 63920). **BOLIVIA: *Beni*:** 40 km S. de Borja a Trinidad, Estación "Los Tajibos", Ballivián (CBF 416); Alrededor de Ibiato, San Javier, Cercado (CBF 3966); cerca de la Estación EBB, prov. Ballivian (CBF 1923); Diquisii, Ibiato, San Javier, Cercado (CBF 3967); Estacion Biologica Beni, Ballivian (CBF 1925, CBF 1941, CBF 1254, CBF 1251, CBF 1252, CBF 1016, CBF 1013, CBF 1253, CBF 1015, CBF 1256, CBF 1255, CBF 1257, CBF 4090); Estacion Biologica Beni, Yacuma, Ballivian (CBF 1947, CBF 1558, CBF 1556, CBF 1563, CBF 1560); El Arroyo, Ibiato, San Javier, Cercado (CBF 3972); El Totaizal, Yacuma, Ballivian (CBF 4085); Espíritu, Ballivian (CBF 1836); Estancia Florida, Yacuma, Ballivian (CBF 4098, CBF 1926, CBF 1950, CBF 1950, CBF 1565); Hacienda Conquista, Ballivian (CBF 4097, CBF 1944, CBF 1946, CBF 1951, CBF 4089); Hacienda El Triunfo, Ballivian (CBF 1953); Hacienda Florida, EBB, Yacuma, Ballivian (CBF 1562, CBF 1922); Hacienda Villa Dorita, Ballivian (CBF 4080, CBF 1927, CBF 1943, CBF 1942, CBF 1939, CBF 1949, CBF 1948, CBF 1945, CBF 1952, CBF 1564, CBF 1559, CBF 1569); Ibiato, Cercado (CBF 1575, CBF 1572, MNK 1130, MNK 990; MNK 1220, MNK 1775, MNK 1229, MNK 1032, MNK 1048, MNK 1016, MNK 1353, MNK 885, MNK 1098, MNK 1133, MNK 1273, MNK 989, MNK 1349, MNK 929, MNK 1114, MNK 1041, MNK 1197, MNK 987, MNK 12886, MNK 1103, MNK 1125, MNK 1187, MNK 1352, MNK 1040, MNK 1347, MNK 1211, MNK 1092, MNK 1350, MNK 1174, MNK 1030, MNK 1039, MNK 1212, MNK 1297, MNK 1196, MNK 1217, MNK 1176, MNK 1198, MNK 1095, MNK 943, MNK 1019, MNK 1173, MNK 1984, MNK 1093, MNK 1071, MNK 1348, MNK 1269, MNK 1047, MNK 1184, MNK 902, MNK 1295, MNK 886, MNK 1090, MNK 1092); Ibiato, San Javier, Cercado (CBF 3944, CBF 3968, CBF 3971); Irachiti, Ibiato, San Javier, Cercado (CBF 3949); Isla de Bosque a la orilla de carretera, Estancia Florida, Yacuma, Ballivian (CBF 4087); Isla de Bosque, Estacion Biologica Beni (EBB), Prov Ballivian (CBF 4093); Isla de Bosque, Hacienta el Triunfo, Ballivian (CBF 4084, CBF 4091, CBF 4086); Isleta entre la Estancias de Villa Dorita y EBB, Provincia Ballivian (CBF 1924); Jipi Japa; Ibiato, San Javier, Cercado (CBF 3970); La Esperanza, Ibiato, San Javier, Cercado (CBF 3965, CBF 3964, CBF 3962); Laguna Azul, Ibiato, San Javier, Cercado (CBF 3948); Laminero, Ballivian (MNK 3006); Nguiraquia Nete Ndesiquia, Ibiato, San Javier, Cercado (CBF 3847); Paichone, Ibiato, San Javier, Cercado (CBF 3977); Perro Quemado, Ibiato, San Javier, Cercado (CBF 3945, CBF 3974, CBF 3976); Plato Zori, Ibiato, San Javier, Cercado (CBF 3946); Province Ballivian (CBF 1929, CBF 1928, CBF 1933, CBF 1932, CBF 1931, CBF 1930, CBF 1940, CBF 1934, CBF 1557); Puesto Carlos, Ibiato, San Javier, Cercado (CBF 3963); ca. 12 kilometers northwest of Limoquije, Camiaco, Marban (AMNH 211664); ca. 23 kilometers west of San Javier, Mamore River, Cercado (AMNH 211666, AMNH 211667, AMNH 211669, AMNH 211670, AMNH 211671, AMNH 211674); ca. 20 kilometers southwest of San Joaquin, Estancia Yotiole, Marmore (AMNH 215066, AMNH 215067); San Joaquin (FMNH 119371); ca. 6 kilometers west of Casarabe, Cercado (AMNH 255864, AMNH 255865, AMNH 255866, AMNH 255867, AMNH 255868, AMNH 255869); Trinidad (AMNH 262659); Quatara, Ibiato, San Javier, Cercado (CBF 3973); Quicuandusurenda, Ibiato, San Javier, Cercado (CBF 3961, CBF 3969, CBF 3975); Reserva EBB, Yacuma, Ballivian (CBF 4086); San Antonio, Ballivian (MNK 3009, MNK 3003, MNK 3007, MNK 3014, MNK 3008, MNK 3011); Yacuma, Ballivian (CBF 4099); Yaranda, Ballivian (MNK 3030). ***Chuquisaca*:** Rosario del Ingre, Hernando Siles (CBF 4260). ***Cochabamba*:** Camp Yaqué, Carrasco (CBF 421); Campamento Yuquí (CBF 420); Rio Chímoré, Carrasco (CBF 1573, CBF 1571, CBF 1570, CBF 1610, CBF 1603, CBF 1503, CBF 1462, CBF 1526, CBF 1521, CBF 1507, CBF 1505, CBF 1543, CBF 1540, CBF 1539, CBF 1538, CBF 1548, CBF 1547, CBF 1546, CBF 1545, CBF 1544, CBF 1554, CBF 1553, CBF 1552, CBF 1551, CBF 1550, CBF 1549, CBF 1555, CBF 1568, CBF 1567, CBF 1566). ***La Paz*:** Santa Cruz de Madid (Santa Ana de Madidi?) (CBF 422). ***Santa Cruz de La Sierra*:** Andres Ibanez (AMNH 244645); Buena Vista, Santa Cruz (AMNH 61801, FMNH 21405, FMNH 21406, FMNH 34351); Buena Vista, Ichilo (MACN 50125, FMNH 34352, FMNH 34353); Campamento Pascana, Estancia San Miguelito, Velasco (MNK 4296); Cordillara Karvami (MNK 4295); El Cedrito, Andres Ibañez (MNK 502); Estancia Curichi, Cordillera (MNK 4792); Hoyada, Pampagrande, Florida (MNK 4645); 142 km N Santa Cruz (CBF 419); 7 km east and 3 km north of Ingenio Mora, Santa Cruz (AMNH 247662); Pampa grande, Florida (MNK 4646); San Lorenzo, Lomario, Nuflo da Chávez (MNK 2284, MNK 2351, MNK 2296, MNK 2310, MNK 2353, MNK 2317, MNK 2203, MNK 2301, MNK 2192, MNK 2354, MNK 2297, MNK 2288, MNK 2205, MNK 2289, MNK 2320, MNK 2291, MNK 2188, MNK 2287, MNK 2316, MNK 2248, MNK 2356, MNK 2298, MNK 2285, MNK 2286, MNK 2321, MNK 2290, MNK 2293, MNK 2198, MNK 2305, MNK 2294, MNK 2236, MNK 2309, MNK 105, MNK 2184, MNK 2244, MNK 2247, MNK 2175, MNK 2159, MNK 2172, MNK 2265, MNK 2237, MNK 2187, MNK 2190, MNK 2195, MNK 2153, MNK 2199, MNK 2225, MNK 2346, MNK 2206, MNK 2196, MNK 2161, MNK 2336, MNK 2241, MNK 2154, MNK 2170, MNK 2181, MNK 2165, MNK 2150, MNK 2186, MNK 2268, MNK 2155, MNK 2151, MNK 2152, MNK 2164, MNK 2332, MNK 2219, MNK 2327, MNK 2271, MNK 2156, MNK 2226, MNK 2168); Totaitú, San Javier, Ñuflo de Chávez (MNK 74); Santiago, Chiquitos (FMNH 105937, FMNH 105936); Exact locality unknown (MACN 50123). **BRAZIL**: ***Alagoas***: Manimbu (MZUSP 7527); Usina Caeté (20 km SSW São Miguel dos Campos) (MN 30687). ***Amazonas*:** Amazon River, south bank, Paritins (AMNH 93116, AMNH 93734, AMNH 93735); Balbina (MZUSP 22751, MZUSP 23167, MZUSP 23168). BR319, km 40, Manaus (INPA 50, INPA 51, INPA 53, INPA 54, INPA 55, INPA 56, INPA 57, INPA 59, INPA 60, INPA 61, INPA 62, INPA 63, INPA 64, INPA 65, INPA 66, INPA 67, INPA 706, INPA 707, INPA 708); BR364, Km 119 (INPA 725); Estrada Aleixo, km 8, São José, Manaus (INPA 49, INPA 58); Estrada Manaus-Itacoatiara, km 46, Manaus (MPEG 7129, MPEG 7130); Igarapé Anibá (MZUSP 19975, MZUSP 19976, MZUSP 19977); Ilha da Embauba, Anavilhana (INPA-CCM 037); Itacoatiara (MZUSP 19979); Lago Canaçari (MZUSP 19978, MZUSP 5109); Lago do Batista, Rio Amazonas (south) (MN 6071); Madeira River, Auara Igarape (AMNH 91708, AMNH 91709, AMNH 91710); Madeira River, Borba (AMNH 92336); Manaus (INPA 52); Reserva de Desenvolvimento Sustentável Amaná, Comunidade Boa Esperança, Maraã (MPEG 37146); Rio Uatumã, Usina hidrelétrica de Balbina (MN 26915, MN 26916, MN 26918, MN 26919, MPEG 22126, MPEG 22127, MPEG 22128, MPEG 22129); Silves, Murucutu (UNB 1900); Villa Bella Imperatriz, Amazon River, south bank, Paritins (AMNH 93117); 40 km da boca do Rio Ariuí, margem direita do Rio Negro (Rio Ariaú, Paraná do Ariaú) (MN 30484). ***Bahia*:** Albanai [Marai?], Rio Tapajos (Este) (NRM 587375); B. da Vitória, Ilhéus (MN 10045); Fazenda Jatoba, Correntina (UNB 1519); Fazenda Pirataquissé (mata do limoeiro); Ilhéus (MN 10974, MN 10975, MN 10976, MN 10977, MN 10978, MN 10979, MN 10980); Japu, Ilhéus (MN 10068); João Pessoa, Rio Jurua (NRM 592340); Lago do Baptista, Rio Amazonas (NRM 591571); Parque Nacional Chapada Diamantina (MHNCI 5587); Piçarrão, Sento Sé (UFPB 6905, UFPB 6906); Rio Amazonas, Igarapé Aribá (NRM 581543); Ribeirão da Fortuna, Buerarema (MN 10504); Serra Negra (MN 30686); Urucatuca, Aritaguá, Ilhéus (MN 10114). ***Espírito Santo*:** São Domingos (MN 23995); Santa Teresa (MN 24003). ***Goiás*:** Anápolis (AMNH 133275, AMNH 133277, AMNH 133346, AMNH 133347, AMNH 133355, AMNH 133357, AMNH 133358, AMNH 133361, AMNH 133362, AMNH 133363, AMNH 133364, AMNH 133365, AMNH 133367, AMNH 133368, AMNH 133369, AMNH 133371, AMNH 133372, AMNH 133373, AMNH 133375, MN 4669, MN 4670, MN 4671, MN 4672, MN 4673, MN 4674, MN 4733, MN 4974, MN 4981, MN 4983, MN 4984, MN 4985, MN 5006, MN 5007, MN 5008, MN 5009, MN 5010); Cana Brava (MZUSP 4130); Estulania, Pouso Alto (MN 10099); Fazenda Bandeirantes, Baliza (UNB 1118); Serra da Mesa (UNB 1602); Vale do Paranã, Foz do Rio Bezerra, Arraias (UNB 2022); 100 Km north Mineiros (MN 24460, MN 24461, MN 24462, MN 24463, MN 24464, MN 24465, MN 24466); 30 Km Mineiros (MN 24467, MN 24468); 10 Km Mineiros (MN 24470, MN 24471, MN 24472, MN 24473, MN 24474); Exact locality unkown (MZUSP 4129). ***Maranhão*:** Barra do Corda (MZUSP 7995, MZUSP 7996, MZUSP 7997); Complexo Ponta da Madeira, São Luis (UFMG 3982, UFMG 3981, UFMG 3980); Estreito (MPEG 23159); Pedra Preta, Arame (MPEG 23178). ***Mato Grosso*:** BR158, Vila Rica (UNB 2539); BR158, Porto Alegre do Norte (UNB 2547); Chapada (MZUSP 3245, MZUSP 3246); Chapada dos Guimarães (AMNH 365); Descalvados Ranch (FMNH 28349, FMNH 28347, FMNH 28348); Estação Ecológica Sesc Pantanal, Barão do Melgaço (MN 64001); Fazenda Pantanalzinho, Porto Espiridão (MN 25903, MN 25904); Maracaju (AMNH 133328, AMNH 133329, AMNH 133330, AMNH 133397); Posto Cuiabá, Estação Ecológica Sesc Pantanal, Barão do Melgaço (MN 64731); Usina Hidrelétrica Jauru, Jauru (MN 69848); Usina Hidrelétrica APM, Manso (UNB 1753); Xavantina (MZUSP 6900). ***Mato Grosso do Sul*:** Fazenda Barma, Brasilândia (MZUSP 28769); Fazenda Primavera, Bataiporã (MZUSP 28768); Parque Nacional Ilha Grande (MHNCI 5660); Urucum, Corumba (AMNH 37166). ***Minas Gerais*:** Araxá (MCN-M 1735); Arceburgo (MZUSP 6613); Araguari (MCN-M 2111, MN 44552); Bambuí (MN 8304, MN 8305, MN 8306, MN 8307, MN 8308, MN 8309, MN 8310, MN 8311); Barão de Cocais (MCN-M 1748); Conceição do Mato Dentro (MCN-M 2514); Comunidade de Palmeiras, Capela Nova (UFMG 4115); Congonhas (MCN-M 2114); Fazenda Batatal, Babilonia, Delfinópolis (MN 10077); Fazenda Bom Retiro, Além Paraíba (MN 5500, MN 5501); Fazenda Carrapicho, São João do Glória (MN 10049); Fazenda Cabuí, Matias Barbosa (MN 10064, MN 10075); Fazenda da Mata, Araguarí (MN 7592); Fazenda do Capão Grande, Passos (MN 10095); Fazenda Lambarí, São João do Glória (MN 10050, MN 10059, MN 10082); Fazenda Palmeiras, São João do Glória (MN 10106); Fazenda Pombal, Volta Grande (MN 2192, MN 2605); Fazendinha Saborá (UFMG 4146); Fazenda São José, Passos (MN 10109); Fazenda Santa Idália, Matias Cardoso, Manga (MN 29073); Fazenda Velha, Matias Barbosa (MN 10081); Formiga (UFMG 157, UFMG 158); Furnas (DEAE), AHE Simplício, Chiador (MN 79024); Irará (MZUSP 394); Lagoa Santa (MN 24011); Mariana (MCN-M 1744); Mangabeira, 2 km North Posses, Minas Novas (UFMG 1104); Mineração Tejucana, Diamantina (UFMG 3085); Rio das Velhas (FMNH 21131); Ribeirao Jordão (300 m do encontro da BR22 e Rio Jordão); Parque Florestal Estadual Rio Doce, Timóteo (UFMG 1572); Passos (MN 10094); Perd, Rio Doce (UFMG 3102); Pompéu (MCN-M 1767, UFMG 156, UFMG 484, UFMG 150, UFMG 148, UFMG 149); Prados (UFMG 738, UFMG 740, UFMG 734, UFMG 969, UFMG 914, UFMG 784, UFMG 920, UFMG 739); probably São João Batista do Glória (MN 24014); probably Bambuí (MN 24025, MN 24026, MN 24027, MN 24028); Rancharia, Matias Barbosa (MN 1006); São João do Glória (MN 10053, MN 10054, MN 10056, MN 10061, MN 10066, MN 10067, MN 10074, MN 10076, MN 10084, MN 10085, MN 10086, MN 10087, MN 10088, MN 10090, MN 10093, MN 10097, MN 10100, MN 10102, MN 10107); Serra São José, Prados (UFMG 303); Tapira (MCN-M 2940); São João do Glória (= São João Batista do Glória) (MN 10046); Usina Hidrelétrica Irapé, Córrego Alegre (MCN-M 2112); Usina Hidrelétrica Nova Ponte (UFPB 1966, UFPB 1968, UFPB 1967); Viçosa (MN 1368); Exact locality unknown (FURB 18679, FURB 6597). ***Pará*:** Alegre, 15 km NE Marapanim (MZUSP 19982); Agrovila União, 19 km S e 18km W de Altamira (MPEG 20157, MPEG 20158); Bagagem, Rio Tocantins (MZUSP 13485); Barcarena (MPEG 43711); Belém (MPEG 939); Belterra (MN 5789); Belterra, 25km S e 26km W de Santarém (MPEG 20153, MPEG 20154, MPEG 20155, MPEG 20156); Cachimbo (MZUSP 8073, MZUSP 19970); Cachoeira do Espelho, Rio Xingu (MZUSP 202924, MZUSP 20923, MZUSP 20924, MZUSP 21301); Canteiro XXX, Usina Hidreletrica Belo Monte, Vitória do Xingu (MPEG 42642); Canoal, margem direita Rio Tocantins, 30 km S de Tucurui (MPEG 12319); Comunidade Lago Juruti Grande, Município Juruti (MPEG 43712); Comunidade Capiranga, Juruti (MPEG 38389); Caxiricatuba, Rio Tapajós (MZUSP 5113); Curralinho, Marajó Island (AMNH 133337, AMNH 133339, AMNH 133340, AMNH 133341, AMNH 133342); Estrada Curuá-Una, 44km S e 40km E de Santarém (MPEG 20152, MPEG 20159); Estrada Belem-Brasilia, km 75 (MPEG 2393); Estrada PA 391, Comunidade do Arací, Santa Bárbara (MPEG 37916); Estrada Santarém-Cuiaba, km 84, BR 165, Santarém (MPEG 4528,MPEG 8116, MPEG 8117, MPEG 8526, MPEG 8527, MPEG 8532); Flora Tapirapó, Aguiri Salobo, Marabá (MPEG 43713); Fordlandia (MZUSP 19969); Foz do Curuá (MZUSP 5110); Igarapé Jaramacaru (MZUSP 19980); Ilha Mexiana (MZS Mam 3584, MZS Mam 3585, MZS Mam 3586, MZS Mam 3588, MZS Mam 3587, MZS Mam 3589); Lote 03, Gleba 29, 73 km NE 45km W Marabá (MPEG 20196); Marabá (MCN-M 2616); Óbidos (MN 5969); Parque Ambiental de Belém, Belém (MPEG 39710); Parque Zoobotânico (MPEG 6763); Pegunha, Santa Bárbara (MPEG 41000); Piquiatuba, Rio Tapajós (MZUSP 5112); Rampá, margem esquerda Rio Tocantins, Tucurui (MPEG 12330); Rio Tapajos, Fordlandia (AMNH 133402); Rio Tapajos, Tamari (AMNH 95111, AMNH 95112, AMNH 95113, AMNH 95115); Rio Tapajos, Igarapé Brabo (AMNH 95116, AMNH 95117); Rio Tocantins, Ilha do Taiuma (AMNH 96310); Rio Trombetas, Cachoeira Porteira, Oriximiná (MPEG 10431); Saude, margem esquerda Rio Tocantins, 170km S Tucurui (MPEG 12332); South Ilha Tocantins 75 km S 18 km E, Rio Tocantins, Tucurui (MPEG 12481, MPEG 12482, MPEG 12483); Taperinha, Santarém (MPEG 22655); Taperinha, Santarém (MPEG 4669, MPEG 4670, MPEG 4671, MPEG 4672, MPEG 4673, MPEG 4677); Tauari, Tapajos River, Santarém (AMNH 95110); Timbozal, margem esquerda Rio Tocantins, 57 km S Tucurui (MPEG 11879, MPEG 11880, MPEG 11881, MPEG 11882, MPEG 11883); Transamazônica km 50, prx. [near] Itaituba (MZUSP 19965); Transamazônica km 75, prx. [near] Itaituba (MZUSP 19966); Transamazônica km 62, prx. [near] Itaituba (MZUSP 19981); Exact locality unknown (MN 2687, MN 2688, ZMB_Mam_1905). ***Paraíba*:** Barra de Mamanguape, Mamanguape (UFPB 6893); BR230, ao lado do IBDF, 1 km do IPE, João Pessoa (UFPB 746); Pedra da Boca, Araruna (UFPB 6875); 5 km de Desterro de Malta (UFPB 744); Exact locality unknown (UFPB 6891, UFPB 6757, UFPB 6287, UFPB 6439). ***Paraná*:** ADEA, Curitiba (MHNCI 1108); Barra do Iguacú, Laranjeira do Sul (MHNCI 166); BR277, próximo Kayser, Ponta Grossa (MHNCI 3902); Clevelândia (FURB 638); Chácara Santo Antônio, Quatro Barras (MHNCI 3898); Curitiba, próximo ao Zoológico (MHNCI 5794); Diamante do Norte (MHNCI 3885); Estação Ecológica Caiuá, Diamante do Norte (MHNCI 3900, MHNCI 3899); Fazenda Coni Flora, Tijuca do Sul (MHNCI 4948, MHNCI 4950); Fazenda Gralha Azul, Fazenda Rio Grande (MHNCI 3896); Fazenda Monte Alegre, Telemaco Borba (MN 68378, MN 68379); Fazenda S. Júlio, Palmas (MHNCI 347); Fazenda Santana, Pinhão (MHNCI 3891); Fazenda Maribondo, Conselheiro Mairinck (MHNCI 1741); Fazenda Capivari, Campina Grande do Sul (MHNCI 1742); Foz do rio Jordão, Captação, Pinhão (MHNCI 2996); Foz do Iguaçu (MHNCI 3888); Guaíra (MHNCI 1739); Ilha do Cristo (proximo a Ilha Grande); Itaipu, Foz do Iguaçu (MHNCI 3897, MHNCI 3964); Itaqui, Guaraquecaba (MHNCI 3889, MHNCI 3893); Itaqui, Guaraquecaba (MHNCI 3890); Jandaia, Apucarana (MN 10078); Klabim, estrada PR160, Club Hamônia Telêmaco Borba (MHNCI 3903); Palmeira (MHNCI 1746); Parque Nacional Foz do Iguaçu (MHNCI 167); Parque Nacional Iguaçu, Foz do Iguaçu (MHNCI 3847); Parque Estadual Vila Rica do Espírito Santo, Fênix (MHNCI 4369); Parque Florestal de Caxambu, Castro (MHNCI 820); Pinhais (MHNCI 5590, MHNCI 5588, MHNCI 5589); Próximo Parque Estadual Vila Rica do Espírito Santo, Fênix (MHNCI 5791); Merces, Curitiba (MHNCI 144); Represa Capivari-Cachoeira, Campina Grande do Sul (MHNCI 3538); Rio do Meio, Barra de Guaratuba (MHNCI 305, MHNCI 303); Rondinha, Pinhão (MHNCI 2997); Terra Nova, Castro (MHNCI 304); Tijuco Alto (MZUSP 27799, MZUSP 27882, MZUSP 27883); Usina Hidrelétrica Caxias, Capitão Leonida Marques (MHNCI 3886, MHNCI 3887); Usina Hidrelétrica Parigot de Souza, B. Alto, Antonina (MHNCI 1997); Usina Hidrelétrica Segredo, Captação-Pinhão (MHNCI 2724); Uusina Hidrelétrica Segredo, Pinhão (MHNCI 3894, MHNCI 5317); Usina Hidrelétrica Segredo, Capitão Leônidas Marques (MHNCI 3895); Vila UHE Segredo, Copel. Pinhão (MHNCI 2998). ***Pernambuco*:** Chã de Peroba, Aldeia, Camaragibe (UFPE 1706); Exu (MZUSP 25594); Poção (MN 1504); Exact locality unknown (UFPB 6989). ***Piauí*:** Aroazes (MM 55384, MN 55385, MN 55386, MN 55387, MN 55388); Brejinho, Coronel José Dias, Parque Nacional Serra da Capivara (MN 59488, MN 59493); Fazenda Concal, Brejo do Piauí (MN 75225); Mangueira do João Paulo, Parque Nacional Serra da Capivara (MN 303393); Oeiras (MN 62999, MN 63000, MN 63001, MN 63002, MN 63003, MN 63004, MN 63005); Parque Nacional Serra da Capivara, São Raimundo Nonato (MN 59477, MN 59479, MN 59484, MN 59487, MN 59494); Passagem Franca (MN 55377, MN 55378, MN 55379, MN 55380, MN 55381, MN 55382, MN 55383); Paulistana (MN 55370, MN 55371, MN 55372, MN 55373, MN 55374, MN 55375, MN 55376); Raimundão, Dirceu Arcoverde (MN 63450, MN 63451); São Raimundo Nonato (MN 55389); São João Vermelho (Guarita), Parque Nacional Serra da Capivara (MN 59469); Vereda do Baixo, João Costa (MN 63507). ***Rio de Janeiro*:** Caxias (MN 1853, MN 2432, MN 2433); Col. Da Taquara, Nova Iguaçu (MN 10070, MN 10073, MN 10079); Fazenda Boa Fé, Teresópolis (MN 7306, MN 7593, MN 7600, MN 7605); Fazenda Santa Clara, Tres Rios (MN 10051); Fazenda Providência, Carmo ((MN 59007); Laje do Muriaé (MN 73291, MN 73301, MN 73552); Maramba, Parque Nacional Itatiaia, Itatiaia (MN 41960); Macieiras, Parque Nacional Itatiaia, Itatiaia (MN 41961); Núcleo Colonial, Duque de Caxias (MN 10044); Praia Grande, Arraial do Cabo (MN 63012); Pedra Branca, Paraty (MN 8464); Pinheiral, Monte Serrat, Parque Nacional Itatiaia, Itatiaia (MN 41926); Reservatório Ribeirão das Lajes, Piraí (MN 75298); Restinga de Marambaia (MN 51653); Rio de Janeiro (NMW St 1026, NMW St 1027); Rio Guarays, Magé (MN 24012); RPPN Nestoda, Natividade (MN 73551); São João do Meio (MN 2431, MN 2434, MN 2435); Vale do Pamparrão, Sumidouro (MN 42859, MN 42860, MN 62812). ***Rio Grande do Sul*:** Alegrete (MCNU 750); Balneareo, Tapes (MCNU 2297); Cambará do Sul (MCN 3372, MCN 3365, MCN 3378); Cerros Verdes, Santana do Livramento (MCNU 1682, MCNU 1683, MCNU 2305); Copesul, Triunfo (MCN 2962); Estrada da Estiva, Taim (MCN 986); Estrada Bacia dos Ratões, Rio Caí, Copesul, Triunfo (MCN 3352); Estância do Tigre, Bagé (MCN 2571); Fazenda Chapada, Dom Feliciano (MCN 3332); Fazenda São Demétrio, Nova Roma do Sul (MCNU 904); Foz de Chapecó, Alpestre (FURB 18225); Garruchos, São Borja (MCN 2553); Garruchos, São Borja (MCN 352); Itá (MCNU 2179); National Forest São Francisco de Paula, São Francisco de Paulo (FURB 9384); Nova Santa Rita (MCNU 2905, MCNU 2904); Nova Roma do Sul (MCNU 906); Osório (MCNU 925, MPEG 22219); Parque da Copesul de Proteção Ambiental, Triunfo (MCN 3406); Pastemon, Porto Alegre (MN 7686); Pólo Petroquímico, Copesul, Triunfo (MCN 2440); Pólo Petroquímico, Triunfo (MCN 2554); P. Raso, Triunfo (MCN 2788); Quinta (AMNH 235966); Rio das Antes, Usina Hidrelétrica 14 de Julho, Bento Gonçalves (MCNU 2394); Rio Grande (MCNU 2317); Rio Turuo Ipê (MCNU 2211); Rosário do Sul (MCNU 738); Santana do Livramento (MCNU 2846, MCNU 2845); Santo Antônio da Patrulha (MCNU 621); Santo Amaro, General Câmara (MCN 3021); São Lourenço (MZUSP 1640, MZUSP 1641, MZUSP 1642, MZUSP 1643, MZUSP 1644, MZUSP 1645, MZUSP 2049, MZUSP 2050, MZUSP 2051, MZUSP 2052, MZUSP 3249); Sentinela do Sul (MCNU 58); Serra do Umbu, São Francisco de Paula (MCNU 76); Usina Hidrelétrica Foz do Chapecó, Area 2, Alpestre (FURB 15326); Exact locality unknown (ZMB_Mam_85946, ZMB_Mam_85943, ZMB_Mam_85787, ZMB_Mam_31710, ZMB_Mam_31703, ZMB_Mam_31225, ZMB_Mam_31223, ZMB_Mam_30265, ZMB_Mam_27845, ZMB_Mam_20470, ZMB_Mam_11700, ZMB_Mam_11696, MZS Mam 3585, MZS Mam 3582, ZMB_Mam_85973, ZMB_Mam_85972, ZMB_Mam_85969, ZMB_Mam_85966, ZMB_Mam_85965, ZMB_Mam_85961, ZMB_Mam_85960, ZMB_Mam_85959, ZMB_Mam_85958, ZMB_Mam_85957, ZMB_Mam_85955, ZMB_Mam_85953, ZMB_Mam_85952, ZMB_Mam_85950, ZMB_Mam_85949). ***Rondônia*:** BR 364, km 49, Samuel (INPA 67); Costa Marques (AMNH 209944); Garimpo Bom Futuro, próximo do Igarapé Santa Cruz, Bom Jardim (MN 42850); Maloca S. Joraem Surumú, Pacaraima (MN 60616); Ouro Preto do Oeste (MPEG 20531); Pimenta Bueno (LF 4953-MN); Usina Hidreletrica Samuel, Porto Velho (UNB 1047); Usina Hidreletrica Samuel, Eletronorte, Porto Velho (UFPE 1229); Usina Hidrelétrica Samuel, margem direita Rio Jamari (MN 27945); Usina Hidrelétrica Samuel, margem direita Rio Jamari, Candeias do Jamari (MN 28503, MN 28504). ***Roraima***: Rio Catrimani (MZUSP 13687). ***Santa Catarina*:** Água Branca street, Blumenau (FURB 6409); BR470 (próximo Segalas); BR470, 300 metros de Lontras (FURB 7000); Blumenau (FURB 18811, ZMB_Mam_6059, FURB 5211, FURB 5362); Blumenau (23° Batalhão de Infataria, Garcia) (FURB 6412); Colonie Hansa (ZMB_Mam_42564, ZMB_Mam_42565); Encano Alto, Indaial (FURB 15030); Engenho Paul Werner street, Blumenau (FURB 20079); Fortaleza, Blumenau (FURB 9998, FURB 18749); Gasparinho, Gaspar (FURB 636); Jaraguá (do Sul) (NMW B2985, NMW B3035, ZMB_Mam_85910); Joinville (MN 7594, MN 7595, MN 7596, MN 7597, NMW B3005, NMW 2648); Lado Vertedouro, Ipuaçu (FURB 12077); Max Hering street, Victor Konder, Blumenau (FURB 20069); Monte Castelo (FURB 680); Parque Artex, Blumenau (FURB 36); Pomerode (FURB 683); próximo Rio Negro, Fragosos, Campo Alegre (MHNCI 1745); Reserva Biologica Estadual Sassafrás, Dr. Pedrinho (FURB 12635); Reserva Vila Velha, Itapoá (MHNCI 5586); Rio da Luz (NMW B3034); Rodeio, Km32 (FURB 9169); Rodovia entre São Domingos e Galvão (FURB 6966); Rua dos Caçadores, Velha, Blumenau (FURB 12018); Theresopolis (NMW 1677, NMW 1676); Usina Hidreletrica Campos Novos, Anita Garibaldi (MCN 3489); Exact locality unkown (FMNH 22436, ZMB_Mam_92498, ZMB_Mam_92496). ***São Paulo*:** Água Funda (MZUSP 6276); Bauru (MZUSP 496); Botucatu (MN 73384, MN 73385); BR153 km, Campos Novos, Paulista (MHNCI 4181); Campestre, Lins (MZUSP 6166); Conchas (MZUSP 13800, MZUSP 13801); Estação Experimental Ubatuba (MN 10092); Fazenda Bandeirante, Presidente Venceslau (MN 10055, MN 10072); Franca (MZUSP 1082); Franco da Rocha (Serra dos Cristais) (MZUSP 19985); Instituto Butantan (ZMB_Mam_36415); Iporanga (Bairro da Serra) (MZUSP 20007); Ipiranga, São Paulo (MZUSP 2389, MZUSP 2937); Piracicaba (ZMB_Mam_60560); Poujinho, Rio Juguiá [Juquiá] (FMNH 92960, FMNH 92962, FMNH 92961, FMNH 92963, FMNH 92964); Praia Grande (FMNH 89854); Primeiro Morro (FMNH 94297, FMNH 94298, FMNH 94299); Ribeirão Água Limpa, Ribeirão Grande (MHNCI 4181); São Jerônimo, Avanhandava (MZUSP 1280); São José dos Campos (MZUSP 22439); Tamanduá, Rio Ipiranga (MZUSP 10431); Ubatuba (MZUSP 2711, MZUSP 3244); Ypanema/Ipanema jetzt Varnhagen (NMW St 1019); Exact locality unknown (MZUSP 20189). ***Sergipe*:** Exact locality unknown (MN 43934, MN 43906). ***Tocantins*:** Projeto Irrigação São João, Palmas (UNB 2546). ***Unknown Locality*:** UFMG 3100, UFMG 3095, UFMG 3097, UFMG 3098, UFMG 3096, MCN 2787, UFPB 6074, ZMB_Mam_1906, NRM 631270, MZS Mam 3610, NMW B3003, NMW B3004, NMW B3001. **COLOMBIA**: ***Amazonas*:** Municipio Leticia, corregimiento Le Chorrera, vereda Cordillera, Rio Igara-Paraná (ICN 15281). ***Antioquia*:** Valdivia (FMNH 70816). ***Arauca***: Fatima, Rio Cobariá (FMNH 92356, FMNH 92355); Rio Cobaria (FMNH 92352, FMNH 92353, FMNH 92354). ***Bolivar*:** San Juan Nepomuceno (IAVH 1204). ***Boyaca*:** Tinjacá (IAVH 7332). ***Caldas*:** Manizales (IAVH 5419). ***Cauca*:** Chisquio (FMNH 88468); Munchique (FMNH 86763); Popayan (AMNH 149274); Quioquio, El Tambo (NRM 602703, NRM 602702); Salento, Quindio (AMNH 33150); Exact locality unknown (AMNH 33147, AMNH 33148, AMNH 33149). ***Cesar*:** Perijá (ICN 19054); Serrania de Rerijá, Aquetin codazzi, vereda Siete de Agosto, El Becerril (ICN 18540). ***Choco***: Riosucio, Vereda Peye, Parque Nacional Natural Los Katios (IAVH 3114); Riosucio, margen derecha de Rio Peye, Parque Nacional Natural Los Katios (IAVH 3914, IAVH 3081); Riosucio, Parque Nacional Natural "Los Katios", Caño de las Mujeres (IAVH 3919). ***Córdoba*:** alto Rio Sinu, Socorro (FMNH 68928, FMNH 68929); alto Rio San Jorge, Catival (FMNH 68932). ***Cundinamarca*:** Anolaima (AMNH 37355, AMNH 37356); El Peñon, Insp de policia talauta (IAVH 5970); Fusagasugá, La Aguadita (ICN 3696); Madrid, Carretera Bogotá – Medellín (ICN 1618); Montes arriba de la Represa del Neusa (ICN 1625). ***Guainía*:** Puerto Inirida (IAVH 6185). ***Guajira*:** Maicao, Corregimiento de Albania, Finca "La Holandesa" (ICN 14947). ***Magdalena*:** Bonda, Santa Marta (AMNH 14663); Parque Nacional Natural "Isla de Salamanca", Caño Clarin Viejo, Los Cocos (IAVH 3910); Santa Marta, Parque Nacional Natural Tayrona, El Cedro (IAVH 4182). ***Meta*:** Caño Lozada (ICN 1620); Extremo sur de Macarena, margen izquierda del Rio Guayabero, frente a desembocadura Finca Saravita "San Juan de Arama" (IAVH 767); Los Micos, San Juan de Arama (FMNH 87907, FMNH 87910, FMNH 87909, FMNH 87908, FMNH 87911, FMNH 87912, FMNH 87913); Macarena (ICN 3694); margen derecha del Rio Guayabero, "El Refugio" (ICN 1623); Mesetas, Vereda San Isidro, cuenca media del Rio Duda (ICN 11622); Parque Nacioinal Natural "La Macarena", Loma del Conejos, 8 km NW El Refúgio (IAVH 1962); Puerto López (ICN 3700, ICN 13001); Rio Guapaya, La Macarena (FMNH 87906); San Martin, Los Guaduales (ICN 5184); sul de la Sierra de la Macarena, margen esquerda del rio Guayapero, Sabana (ICN 3693); Villavicencio (AMNH 136252, AMNH 136254, ICN 1442); Villavicencio, SE Selva entre Caño Aguas Claras e Rio Ocoa (ICN 1619); Villavicencio, al sureste, cerca a Ocoa, Hacienda "La Graciela" (ICN 1617). ***Norte de Santander*:** Charalá, Insp. Policia (IAVH 7051); El Porvenir, Rio Cubugan (FMNH 92357); La Argentina, Rio Cubugon (FMNH 92358); Parque Nacional Tamá Natural, Toledo, vereda El Diamante (IAVH 7054); Parque Nacional Tamá Natural, Alto de Las Brujas (IAVH 7063). ***Putumayo*:** Lower Rio Guamuez (ICN 1560); Rio Mecaya, Caqueta (Putumayo) (FMNH 70817, FMNH 70818, FMNH 70819, FMNH 70820). ***Santander*:** Charalá, Virolin, Rio Luisito (ICN 6699); Encino (ICN 17497); município San Cayetuno, vereda Tabiro, finca la Ortencia, próximo al area metropolitana de Cúcuta, alt 963 msnm (ICN 21450); San Gil (ICN 4713). ***Sucre*:** Coloso (FMNH 68930). ***Vichada*:** Parque Nacional Natural "El Tuparro", Fundo Mata Grande (IAVH 3913). **COSTA RICA**: ***Cartago***: Exact locality unknown (ZMB_Mam_85903, ZMB_Mam_25877, ZMB_Mam_85947, ZMB_Mam_85908). ***Puntarenas*:** Palmar (AMNH 139318, AMNH 139319). ***San Carlos*:** Cataratos (AMNH 139834); Exact locality unknow (FMNH 34191). ***San José*:** Barania [?] (ZMB_Mam_6061); Exact locality unknow (ZMB_Mam_3175, ZMB_Mam_3176). **ECUADOR: *Bolivar*:** Caluma, Caluma (San Antonio) (QCAZ 700, QCAZ 701). ***Carchi*:** Río Blanco, Nueva vía a San Lorenzo, Chinambi (QCAZ 2195, QCAZ 2196, QCAZ 2197). ***Cotopaxi*:** Reserva La Otonga, San Francisco de Las Pampas (QCAZ 697). ***El Oro***: La Chonta, Santa Rosa (AMNH 46553). ***Esmeraldas*:** Caimito (QCAZ 8986); Cupa (QCAZ 696); Esmeraldas (MECN 008); Parroquia Tabiazo, 200 m del estero Taquiana (MEPN 73180); Viche (Cantón Quinindé) (MEPN 7381, MEPN 7380). ***Loja*:** Guachanamá (AMNH 46555). ***Guayas*:** Terminal de La Troncal (MECN sn). ***Manabí*:** (QCAZ 141). ***Morona Santiago*:** Río Miriume, Sucúa (QCAZ 699). ***Pastaza*:** Rio Pindo Yaco (FMNH 43285, FMNH 43286, FMNH 43287); Sarayacu (AMNH 67710). ***Pichincha*:** Cerca Río Pachijal, Las Tolas (QCAZ 1046); Cruz Loma 5, Quito (MEPN 7278); Guayllabamba, Quito (MECN 1925); Finca La Poderosa Crisaga, Cantón Pedro Vicente Maldonado (MECN 3378); Las Maquinas (AMNH 66754); Las Tolas (QCAZ 142); Perucho (NRM 641325); Tinalandia, La Unión del Toachi (QCAZ 140). ***Orellana*:** Avila Viejo, Avila Viejo (QCAZ 3382); Avila, Oriente (MEPN 10807); San José, Canton Loreto (MEPN 8495, MEPN 8490, MEPN 8492, MEPN 8487, MEPN 8488). ***Zamora Chinchipe*:** Shaime, Río Nangaritza (QCAZ 2534). ***Exact Locality Unknown*** (MZS Mam 3581). **FRENCH GUIANA:** near Sinnamary, Paracou (AMNH 266483, AMNH 267012). **GUATEMALA**: ***Izabal***: Escobas (FMNH 41890); Bobos (FMNH 41585); Los Amates (FMNH 15968, FMNH 15969, FMNH 15967); Exact locality unknown (AMNH 177078); ***Vera Paz*:** Finca la Providencia, near San Cristobal (ZMB_Mam_38347, ZMB_Mam_38346, ZMB_Mam_38344, ZMB_Mam_38343, ZMB_Mam_38342, ZMB_Mam_38341, ZMB_Mam_38340); Exact locality unknown (MZS Mam 3578). **GUYANA: *Cuyuni-Mazacuni*:** Kartabo (AMNH 42441, AMNH 42442, AMNH 42914); Exact locality unknow (AMNH 140496, AMNH 42883). ***Exact Locality Unknown*:** ZMB_Mam_85936. **HONDURAS**: ***Copán*:** Roman River, Santa Rosa (AMNH 24229, AMNH 24230). ***Francisco Morazán*:** Cantoral, Tegucigalpa (AMNH 126135, AMNH 126136); La Cueva Archaga, Tegucigalpa (AMNH 128131, AMNH 128132, AMNH 128134, AMNH 128135, AMNH 128136, AMNH 128137, AMNH 128138); Tegucigalpa (AMNH 127562). **MEXICO**: ***Chihuahua*:** Sierra Madres, Rio Almas (AMNH 17476). ***Coahuila*:** Sabinas (FMNH 14008). ***Colima*:** Pueblo Juarez (AMNH 171919, AMNH 171920, AMNH 171921, AMNH 171922). ***Jalisco*:** Las Canoas (AMNH 26007, AMNH 26008); Volcan de Fuego, Tonila (AMNH 2134/2656). ***Michoacan*:** Apatzingan (FMNH 51963, FMNH 51964, FMNH 52246); Tancitaro (FMNH 51392, FMNH 51962). ***Oaxaca*:** Las Tejas, Tehuantepec (AMNH 207420); Reforma (FMNH 15857); Tapanatepec (AMNH 172715); Tehuantepec (AMNH 208909, AMNH 208910, AMNH 208911, AMNH 208912). ***Sinaloa*:** Escuinapa (AMNH 24047, AMNH 24048, AMNH 24049, AMNH 24050, AMNH 24052, AMNH 24053, AMNH 24054, AMNH 24055, AMNH 24056, AMNH 24057, AMNH 24058, AMNH 24059, AMNH 24060, AMNH 24061, AMNH 24062, AMNH 24063, AMNH 24731, AMNH 24732); Mazotlan (ZMB_Mam_14228); Presidio, Mazatlan (Mexique, État de Sinaloa, Mazatlán) (MZS Mam 3579, MZS Mam 3580); Presidio prope, Mazatlan (ZMB_Mam_60580); Exact locality unknow (AMNH 19583). ***Tamaulipas*:** 10 miles south of Ciudad Mante (AMNH 176676). ***Veracruz*:** San Carlos (FMNH 11089); Tampico (AMNH 1622); Exact locality unknow (NRM 590855). ***Yucatan*:** Actun (Excavation) (AMNH 212682); Chichen Itza (FMNH 63921); Zanatepec (AMNH 247450). **NICARAGUA**: 8 miles west of Managua, Lake Jiloa (AMNH 176675); Lavala (AMNH 28422); Pena Blanca (AMNH 29828); Rio coco (AMNH 29276); Sapota (NRM 583791); Exact locality unkown (AMNH 28445). **PANAMA**: ***Canal Zone*:** (FMNH 30885). ***Chiriqui*:** Boquerón (AMNH 18888, AMNH 18889, AMNH 18890); ***Locality Unknown*:** (ZMB_Mam_21652). **PARAGUAY**: ***Alto Guarani*:** Puerto Guarani (MACN 28225). ***Canindeyú*:** Mbaracayu Reserva Natural del Bosque, 2 km NE central camp (Lagunita) (MNHNP 917). ***Central*:** Colonia Nueva Italia (FMNH 54355, FMNH 54354); Lambare (ZMB_Mam_85906). ***Chaco*:** 58 km SW Agua Dulce (MNHNP 542); 4 km S Madrejon, casa de admin, Parque Nacional Defensores del Chaco (MNHNP 541); Estancia Deolindo (NRM 600182). ***Concepción*:** Parque Nacional Serrania San Luís (MNHNP 2506); Estancia Cerrito (MNHNP 2507). ***San Pedro*:** Ybapobo (FMNH 26649). ***Locality Unknown*:** (ZMB_Mam_85899). **PERU: *Cajamarca*:** Caserio de San Juan, Distrito San Ignacio, San Ignacio (MUSM 8720). ***Cuzco*:** Hacienda Luisiana on R io Apurimac (AMNH 208100); Hacienda Villa Carmen, Cosñipata (FMNH 84244). ***Huanuco*:** Exito (FMNH 30476); Hacienda Vista Alegre (FMNH 30479, FMNH 30478, FMNH 30477); Rio Chinchao (FMNH 30489); Rio Yuyapichis, Nebenfluss des Pachitea, ca. 260m Höhe, Panguana (9°37' S, 74°56'W) (SMNS 40192). ***Junin*:** Chanchamayo (FMNH 65801). ***Loreto*:** 25 km S de Iquitos, Estacion Biologica Allpahuayo, Iquitos, Maynas (MUSM 16502); Iquitos (AMNH 98462, AMNH 98463, AMNH 98515, AMNH 99172, AMNH 99173, AMNH 99174); Requena, Rio Galvez, San Juan, Yaquerana, Maynas (MUSM 11088, MUSM 11089); Rio Alto Ucayali (AMNH 147494); Rio Amazonas, Iquitos (AMNH 98458, AMNH 98465); Rio Samiria, Nauta (FMNH 123022); San Juan, Galvez River (AMNH 268229); Yurimaguas, Alto Amazonas (MUSM 2052). ***Madre de Dios*:** Reserva de Cusco Amazónico, 15 km NE. Pto. [Puerto] Maldonado, Distrito Las Piedras, província Tambopata (MUSM 6113). ***Pasco*:** Delfin, Pozuzo, Oxapampa (MUSM 12154); Exact locality unknown (FMNH 30480, FMNH 30481, FMNH 30482, FMNH 30483, FMNH 30490, FMNH 30491, FMNH 30484, FMNH 30486, FMNH 30487, FMNH 30485). ***Ucayali*:** Pucallpa, Callaria, Coronel Portillo (MUSM 6003). **SURINAME**: Albina (ZMB_Mam_85940, ZMB_Mam_85939, ZMB_Mam_85938, ZMB_Mam_85934, ZMB_Mam_85933, ZMB_Mam_85932, ZMB_Mam_85931); Saramacca river, Loksie Hatti, Brokopondo (FMNH 95452); Exact locality unknown (SMNS 26647, SMNS 902, SMNS 26646, SMNS 26645). **TRINIDAD AND TOBAGO:** Caparo, Caroni county (AMNH 7549/5942). **UNITED STATES: *Florida*:** Highlands County (AMNH 242336, AMNH 242661); Loveless (FMNH 198914); 8 miles south of Lake Placid, Archbold Biological Station, Highlands County (AMNH 215329, AMNH 240531, AMNH 242337, AMNH 242339, AMNH 242663, AMNH 253584); 6 miles south of Lake Placid on SR-17, Highlands county (AMNH 242662). ***Georgia*:** Saint Catherine's Island, Liberty County (AMNH 245485, AMNH 245642). ***Texas*:** Brownsville, Cameron county (AMNH 3000); Blanco county (FMNH 39304, FMNH 39305, FMNH 39307); Comfort, Kendall county (AMNH 148798, AMNH 148798); Cameron (FMNH 8858); Dalas (SMNS 47455); Kerrville, Kerr county (AMNH 182075); Kyle, Hays (FMNH 39306); McAllen, Hidalgo County (AMNH 35650); Pierce-Runnells Ranch, 6 miles N Bay City, Matagorda (FMNH 104996, FMNH 104997); Rockport, Aransas county (AMNH 7278); Travis (FMNH 134417); 8 miles S of Del Rio, Val Verde (FMNH 48357); 5 miles E of Sealy, Austin (FMNH 51801); 5 miles NW of Kyle, Hays (FMNH 51802); 5 miles W of Marquez, Leon (FMNH 54323); 12 miles W of Coleman (FMNH 55664); 1/2 miles N Grandview, Johnson (FMNH 122059); Exact locality unknow (AMNH 4947, AMNH 4949). **URUGUAY**: ***Artigas*:** 6 km north northwest of Belen (AMNH 205725). ***Canelones*:** Balneario Los Titanes (MNHN 959). ***Cerro Largo***: Road to Caputti II (ZVCM 5532); Sierra de Vat (MNHN 6929). ***Colonia*:** Colonia Suiza (ZVCM 1276). ***Durazno*:** 15 kilometers north northwest of San Jorge, Rio Negro (AMNH 205726). ***Flores*:** Arroyo Catalán Grande (MNHN 4698); Arroyo Sepulturas, Rio Cuareim (MNHN 3340); Arroyo la Invernada (ZVCM 125, ZVCM 124, ZVCM 123); Cuchilla Villasboas (MNHN 1928). ***Florida*:** (MNHN 3369). ***Lavalleja*:** 12 kilometers west southwest of Zapican (AMNH 205727). ***Rio Negro*:** Alrededor de la ciudad de Fray Bentos (MNHN 3395); Arroyo Bopicuá a 2km de su Boca (ZVCM s/n AL 1131); Bopicuá (MNHN 2775); Young (MNHN 690). ***Rivera*:** Platón (MNHN 6327); Valle del Lunarejo (MNHN 7294). ***San José*:** Paso del Rey (ZVCM 386). ***Soriano*:** Costa del Rio San Salvador (MNHN 6279); Estancia Santa Elena, Arroyo Perdido (MNHN 687, MNHN 686, MNHN 1313, MNHN 828, MNHN 828). ***Tacuarembo*:** Punta del Arroyo Laureles (MNHN 1205); 40 kilometers northwest of Tacuarembo (AMNH 205728). ***Treinta y Tres*:** Estancia "Doña Alba", 5° Seccional Policial, camino verinal al Arroyo Avestruz Grande (ZVCM 5530); Próximo al Arroyo Sarandi Grande Estancia La Mimi (MNHN 2634); Rio Negro 7 km agua arriba de la barracon el Rio Tacuarembo (MNHN 1181); Ruta 8 km (MNHN 4357); Exactly locality unknown (MNHN 3296). **VENEZUELA**: ***Amazonas*:** Atures, entre Provincial y Galipero, N del Puerto Ayacucho (EBRG 4285); Huaynia River (AMNH 78517); Puerto Ayacucho, Hato Atures, Atures (EBRG 2760); Puerto Ayacucho, Cerro sede CAICET, Ature (EBRG 10634); San Carlos do Rio Negro (MBUCV 62). ***Anzoategui*:** 15 km E Puerto Pirito, Quebrada Hoces (EBRG 22225). ***Aragua*:** Estacion Biologica Rancho Grande (EBRG 2174); Los Riitos, Rancho Grande (EBRG 690, EBRG 691); Parque Nacional Henri Pittier Rancho Grande, entre Paisa Mencho y Riitos (EBRG 18); Parque Nacional Henri Pittier Jardin Estacion Biologica Rancho Grande, entre Paisa Mencho y Riitos (EBRG 17); Rancho Grande (AMNH 144828); Urbanizacion Cata, Cata (EBRG 27566, ERBG 27567). ***Barinas*:** Reserva Florestal Ticoporo, unidad II, Compartimiento 9 (EBRG 15788); Reserva Floresta Ticoporo, unidad III, compartimiento 14 a 3 km Reserva Fauna Sabana de Anaro (EBRG 20223). ***Bolivar*:** Caño Maniapure (EBRG 1034); Carretera Caicara-San Juan de Manapiare (EBRG 2229); Puesto de la Guardia Nacional en el Venado (EBRG 782); Represa El Guri, Operacion Rescate (EBRG 1114, EBRG 1193, EBRG 1192, EBRG 1116, EBRG 1115); Serrania de lso Pijiguaos, 140km SO de Caicara (EBRG 15847). ***Carabobo*:** Fila la Josefina, 11 km de Tronconero cerca Carretera (EBRG 10128). ***Cojedes*:** Apartadero (EBRG 1754, EBRG 1753); Hato Nuevo (EBRG 779, EBRG 780); Agropecuaria Las Galeras, Galeras del Pao, Rio Tinaco (EBRG 20835). ***Distrito Capital*:** Falda Sur del Avila (MBUCV 143). ***Guarico*:** 5 km NE San José de Guarite (MBUCV 5348). ***Lara*:** Cumbre Las Trojas, 45 km al Sur de Cabudare (EBRG 2272, EBRG 2271). ***Merida*:** Valle, Libertador (AMNH 33151). ***Miranda*:** Est. Exp. Rio Negro (U.S.R.), Miranda (MBUCV 3627, MBUCV 3810, MBUCV 5639); Est. Exp. Rio Negro (U.S.R.), a 30km de Caucaqua (MBUCV 4089, MBUCV 4256); Parque Vinicio Adames, Hoya de la puerta, Tazon (MBUCV 5637); Ruta 4, La Morita, San Antonio de los Altos (MBUCV 3005, MBUCV 3006);San José de los Altos (MBUCV 3806). ***Monagas*:** Finca dona Nar, 3 km El Tejero (EBRG 22471). ***Nueva Esparta*:** Sur de San Sebastián em Parque Nacional Cerro El Copey, Nueva Esparta (MBUCV 5278). ***Sucre*:** Parque Nacional Peninsula de Paria, NW Melenas (EBRG 17584); Peninsula de Araya, afuera de Guayacan (EBRG 26038). ***Tachira*:** La Fria (MBUCV 1528). ***Yaracuy*:** Hacienda el Zinc, Sierra de Aroa, Sucre (EBRG 29788); Parque Nacional Yurubi, sector El Silencio (EBRG 28782); Urama (MBUCV 625). ***Zulia*:** 20 miles SW Maracaibo (FMNH 18749, FMNH 18748, FMNH 18751, FMNH 18752, FMNH 18750, FMNH 18753, FMNH 18755, FMNH 18754); Maracaibo (FMNH 20537); near Maracaibo (FMNH 18757, FMNH 18756, FMNH 18759, FMNH 18758, FMNH 18760, FMNH 18761). **LOCALITY UNKNOWN**: NMW B3006, NMW B3007, NMW B3002, NMW B 3000, NMW sn2, SMNS 26649, SMNS 26650, MZS Mam 3590, ZMB_Mam_2743, NRM 582918, NRM 20115448, NRM 532077, MPEG 11880, MNHN 7294, MNHN 7348, MNHN7372, MNHN 6394, MN 982, MN 7601, MN 7602, MN 7603, MN 73, MN 46610, MN 2692, MN 2687, MN 2688, MN 24029, MN 24015, MN 24016, MN 24017, MN 24018, MN 24020, MN 24021, MN 24009, MN 24010, MN 23996, MN 23998, MN 23999, MN 24000, MN 24001, MN 2365, MN 20423, MN 1679, MN 17509, MN 1407, MN 1141, MCN 3378, MCN 2779, MBUCV 62, ICN 3034, ICN 16397, SMNS 49809, ZMB_Mam_6060, NRM 582328, NRM 586605.

***Dasypus pilosus* Fitzinger, 1856**

**PERU**: ***Huanáco*:** Apatogocha (Acomavo), Distrito Chinchao, prov. Huánuco (MUSM 2056). ***Pasco*:** Santa Barbara, Distrito Huancabamba, Prov. Oxapampa (MUSM 24214). ***San Martin*:** Parque Nacional Rio Abiseo, distrito Huicungo, Prov. Mariscal Cáceres (MUSM 7499, MUSM 7500); Parque Nacional Rio Abiseo, La playa,distrito Huicungo, Prov. Mariscal Cáceres (MUSM 7501, MUSM 7502); Parque Nacional Rio Abiseo, Las Palmas, Prov. Mariscal Cáceres (MUSM 7503); Parque Nacional Rio Abiseo, Rio Colorado, Prov. Mariscal Cáceres (MUSM 7504, MUSM 7505). ***Locality unknown*:** NMW St 222.

***Dasypus sabanicola* Mondolfi, 1968**

**COLOMBIA**: ***Arauca*:** Estación Biologica "El Guajal" Proyecto Chiguiro, Puerto Rondon (IAVH 5335); Finca el Vanero, cerca de 13 km SSW de município Arauca (IAVH 6188). ***Casanare*:** Maní, Hacienda La Floresta, Altura 260m (ICN 5451). ***Cundinamarca*:** Municipio Tocaima (ICN 1624). ***Meta*:** Puerto Gaitán, vereda Alto Tillauá, Finca Brisalia. Alt 150m (3°49' 33.6'' N; 68° 52' 25.7" W) (ICN 21452). ***Vichada*:** 50 km al SE del bajo "Las Topias" (46 km al W del Tapón) (IAVH 534); Hacienda Portuguesa (ICN 3076); Hacienda Portuguesa, 35 minutos em carro al Sur de Caviona (Caviona esta frente a municipio de Orocué-Meta) (ICN 3075); Fundo "Mata Grande" al occidonte del Fundo "Canaima" em Sabana (IAVH 434); Puerto Carreño, Finca "El Chaparral" a 30 km de Puerto Carreño (IAVH 728). **VENEZUELA**: ***Anzoategui*:** Mamo (MHNLS 5803, MHNLS 5804, MHNLS 5805, MHNLS 5806, MHNLS 5807, MHNLS 5808, MHNLS 5809, MHNLS 5810, MHNLS 5811, MHNLS 5812, MHNLS 5813); Sabanas, Rio Caris, Norte de Paso Los Cocos (MBUCV 3101); Exact locality unknown (MLP 22.II.00.6). ***Apure*:** Alrededores Cerro La Pica, Rio Capanaparo (MHNLS 7000); Finca Laguna Clara (La Trinidad), Municipio Cunaviche, Distrito San Fernando (EBRG 963, EBRG 964); Hato Aguas Claras (50km SO de Palmarito), Distirto Páez (EBRG 966, EBRG 967); Hato El Frio (Fundación Manirito), Carretera El Samán-Mantecal (EBRG 15726, EBRG 15727); Hato El Frio (Carboncito) (EBRG 1889); Hato Lost Matapalos (Cerca de Achaguas) (EBRG 968); Hato Macanillal (Laq. El Tigre), Distrito Achaguas (EBRG 965); La Trinidad de Arauca (Médano de Castillo), Distrito Muñoz (EBRG 783); 41 km NW of Puerto Páez, altitude 76m (EBRG 6492). ***Bolivar*:** Carretera El Tigre-Ciudad Bolivar (EBRG 25573); Morichal Tirigua, Carretera Ciudad Bolivar-Ciudad Piar, km 50 (MHNLS 6601); Represa Guri, Rio Caroní (MBUCV 1669, MBUCV 1670). ***Guarico*:** Carretera entre las Mercedes-Santa Rita (EBRG 1890). ***Monagas*:** Campamento Conare, Chagaramas (MHNLS 5802); Paso Nuevo aproximado 30km Sur del Temblador (EBRG 1891); Uverito (MBUCV 5640, MBUCV 5641). Exact locality unknown (MBUCV 2963, MBUCV 4915).

***Dasypus mazzai* Yepes, 1933**

**ARGENTINA**: ***Jujuy*:** Abra de Cañas, El Monolito, Depto Valle Grande (CML 1809); El Alto, Depto Ledesma (MLP 30.III.90.4); Fca La Realidad Dpto. Ledesma (MLP 30.III.90.3; MLP 30.III.90.I); La Mauricia, Depto Ledesma (MLP 30.III.90.5); La Realidad 25km, Caimancito, Depto Ledesma, (MLP 10.II.99.6); prox. 34.5 km al E. de caimancito, 410 msn.m, Rio San Francico, Fca. La Realidad, Depto Ledesma (MLP 30.III.90.2); Rio Candelaria Fal Caulario, Depto Ledesma (MLP 30.III.90.7); Serania de Santa Bárbara, Depto Santa Barbara (CML 4549). ***Salta*:** San Andres, Depto Oran (MLP 30.III.90.8); Tabacal, Depto Orán (MACN 13222, MACN 31.273).

***Dasypus septemcinctus* Linnaeus, 1758**

***Dasypus septemcinctus septemcinctus* Linnaeus, 1758**

**ARGENTINA**: ***Salta*:** Tabacal, Depto Orán (MACN 3323). **BOLIVIA**: ***Santa Cruz de la Sierra*:** Los Fierros, Parque Nacional Noel Kempff, provincia Velazco (MNK 3198). **BRAZIL**: ***Bahia*:** Chapada Diamantina, Remanso, Lençois (UERJ-CD121, UERJ-CD 022, UERJ-CD 06); Fazenda Jatoba, Correntina (UNB 1518); Reserva Biológicas Pau Brasil, 15 km NW de Porto Seguro (UFPB 585). ***Distrito Federal*:** Águas Emendadas (UNB 1584); Área Alfa, Brasilia (UNB 1475); Brasilia (MZUSP 19983); Córrego, Saia Velha (UNB 2102); Fazenda Água Limpa, Brasília (UNB 2996, UNB 2101, UNB 2103, UNB 2104); Parque Nacional, Brasilia (UNB 1417); Proximidade do Parque Nacional de Brasília (MN 24469); Reserva Ecológica do IBGE (UNB 1476, UNB 2992, UNB 2993, UNB 2994, UNB 2995, UNB 2997, UNB 2998, UNB 2999); Reserva Biológica de Águas Emendadas (UNB 853). ***Goiás*:** Anapolis (AMNH 133258); Parque Nacional das Emas (MN 59336, UFMG 4140); Rio São Miguel (MN 2366). ***Maranhão*:** Barra do Corda (MZUSP 7991, MZUSP 7992, MZUSP 7992, MZUSP 7993, MZUSP 7994). ***Mato Grosso*:** Água Limpa (MZUSP-ML 1263); Chapada (MZUSP 3247, MZUSP 3248); Cocalinho (MZUSP-APC 406); Reserva Xavante, Rio das Mortes (UNB 2815); São Domingos (Rio das mortes) (MZUSP 7031, MZUSP 7032, MZUSP 7033); UHE Manso, Chapada dos Guimarães (UNB 795); Vila Bela de Santissima Trindade (MZUSP-APC 3069); Xavantina, Rio das Mortes (MN 23989). ***Minas Gerais*:** Alto da Consulta, Poços de Caldas (MN 24094); Botumirim (MCN-M 2113); Congonhas, 1131 m.a.s.l. (MCN-M 2530); Fazenda Velha, Matias Barbosa (MN 10091); Fazenda Vargem, Matias Barbosa (MN 10062); Fazenda Levantina, Camandocaia (MZUSP 19984); Itabirito (MCN-M 1794); Prados (UFMG 737, UFMG 782); Provavelmente Matias Barbosa (MN 24005); São João do Glória (= São João Batista do Glória) (MN 10071); Serra da Bandeira, 120 km Barreiras no Caminho para Goiás (MN 4291). ***Pará*:** Cachimbo (MZUSP 8111); Fazenda Teso, Soure, Ilha de Marajó (MN 2370); Ilha de Marajó (MPEG 33); Marajó, Soure, Fazenda Teso (MZUSP 9970); Rio Tocantins, Cametá (MZUSP 5111). ***Paraná*:** Almirante Tamandaré (MHNCI 1107); Br 151, proximo Rio dos Papagaios, Palmeira (MHNCI 1743); Bairro Alto, Curitiba (MHNCI 2782); Boa Vista, Curitiba (MHNCI 2728); Carambeí (MHNCI 4949); Colombo (MHNCI 1744); Colonia Santo Antônio, São José (MHNCI 302); Fazenda Monte Alegre, Klabim, PR 160, Jelêmarco Borba (MHNCI 3901); Fazenda Monte Alegre, Telemaco Borba (MN 68375); Guarapuava (MHNCI 3883); Passo Amarelo, Fazenda Rio Grande (MHNCI 3352). ***Piauí*:** Guadalupe (UFPB-AF713); Parque Nacional da Serra da Capivara, Brejinho, Coronel José Dias (MN 63474); Parque Nacional da Serra da Capivara, São Raimundo Nonato (MN 59478); Raimundão, Dirceu Arcoverde (MN 63454). ***Rio de Janeiro*:** Hospital, Paracambi (MN 53376); Paracambi (MN 55051, MN 55052, MN 55053); Triunfo, Santa Maria Madalena (MN 51652); Exact locality unknown (MN 24007). ***Rondônia*:** Chapadão na divisa do Jamarí e Guaporé, Cabeceirass do Urú, Alto Gi-Paraná (MN 2693). ***Santa Catarina*:** Passos Maia (FURB 18780); PCH Santa Luzia, Alto São Domingos (FURB 20090); Km 164, BR 470 (FURB 20101); São Domingos (FURB 9109); Exact locality unknown (MHNCI 348, MHNCI 349). ***São Paulo*:** Estação Ecológica Santa Bárbara, Águas de Santa Bárbara (MZUSP-APC1171); Fazenda Varzão, Municipio de Lins (FMNH 52354); Orlandia (MZUSP 5857). ***Locality unknown***: MACN 2044, NMW B3013, NMW B3014, ZMB_Mam_3194, UNB 3340, MPEG 38339, MPEG 38340, MPEG 38341, UFMG 3099, MN 2362, MN 2367, MN 23997, MN 24022, MN 24024, MN 25707). **PARAGUAY**: Estancia Roma III, Canindeyu (MNHNP 3365). ***LOCALITY UNKNOWN*:** MN 2362, MN 2367, MN 23997, MN 24022, MN 24024, MN 25707, MPEG 38339, MPEG 38340, MPEG 38341, UFMG 3099.

***Dasypus septemcinctus hybridus* (Desmarest, 1804)**

**ARGENTINA**: ***Buenos Aires*:** Azul (MNHN 1388, MNHN 1389, MNHN 141); Bolivar (MACN 17310); Buenos Aires (MACN 113, MACN 29877, MLP 16.IX.35.36, MN 1435); Calle 58 y 9, La Plata (MLP 1.I.03.69); Campo La Tablada, Coronel Suárez (MLP 1.I.03.65); Chascomus (MLP 1.I.03.67, MLP 2.III.00.5, MLP 5.IX.97.3); Coronel Suarez (MLP 1.I.03.68); Colonia Mantiales, Chascomus (MLP 3.X.94.3); Cristiano Muerto (MLP 867); Ecas (MLP 1879); Estabelecimiento "Los Talas", Alvares Jande (MLP 2.III.00.15); Estacion Girondo (NRM 580174, NRM 580365); Fcia San Pedro, Pdo de Castelli (MLP 5.VIII. 98.4); Jardin Zoologico, La Plata (MLP 4.VIII.98.10, MLP 870); Juan Geronimo, Punto Indio (MLP 1980); Juarez (MACN 54156); Lafrida (MN 24095); La Plata (MLP 16.IX.35.41, MLP 16.IX.35.42, MLP 16.IX.35.43, MLP 16.IX.35.45, MLP 16.IX.35.46); Pdo. Pila, Buenos Aires (MLP 1.I.03.71); Punta Indio, Punta Piedras (MLP 1975, MLP 1976); Rauch (MACN 13220); Rio Sauce Grande, Pdo de Monte Hermoso (MLP 3.X.96.1); Ruta 3 entre Lamadrid y Olava (MACN 13900); Tapalqué (MACN 28.86); Vergara (MLP 1977); Exact locality unknown (MACN 32174, MACN 32175, MBUCV 2905, MLP 16.IX.35.48, MLP 16.IX.35.53, MLP 16.IX.35.54, MLP 16.IX.35.55, MLP 16.IX.35.37, MLP 16.IX.35.38, MLP 16.IX.35.39, MLP 16.IX.35.40, MLP 16.IX.35.47, MLP 16.IX.35.49, MLP 16.IX.35.50, MLP 16.IX.35.51, MLP 16.IX.35.52, MLP 868, NRM 580593); ***Chaco*:** Pampa de los Indios, Pte Rocha (MACN 3017, MACN 3018); Exact locality unknown (MACN 36980). ***Corrientes*:** Mercedes (MACN 34733); Santo Tome, Ecia, Santo Antonia (MLP 18.XI.99.7). ***Jujuy*:** Exact locality unknown (MACN 35148). ***Mendoza*:** Exact locality unknown (AMNH 40068). ***Misiones*:** Exact locality unknown (MACN 251). ***Santa Fé*:** Villa Guillermina (MACN 33176). ***Exact locality unknown*:** NRM 580391, NRM 600183, NRM 611693, MACN 34669. **BRAZIL**: ***Rio Grande do Sul*:** Cambará do Sul (MCN 3377); Cerros Verdes, Santana do Livramento (MCNU 1684, MCNU 2513); Cabeceiras do rio Camaguã, Dom Pedrito (MPEG 22199); Eldorado do Sul (MCNU 2503); Estância da Glória, Ervalia (MN 24006); Estância das Goiabeiras, Santana do Livramento (MCN 99); Pinheiro, Santa Maria (MPEG 22218); Santana do Livramento (MCNU 2182); São Gabriel (MCNU 2205); Vila Seca/Arroio Marrecas, Caxias do Sul (MCNU 2390); Exact locality unknown: ZMB_Mam_85928, ZMB_Mam_85924. ***Exact locality unknown*:**NMW 3012, ZMB_Mam_1903. **URUGUAY**: ***Artigas*:** Ay° Tres Cruces Grande (MNHN 6401); 32 km NW de Artigas Rio Cuareim (ZVCM 847); 6 kilometers north northwest of Belen (AMNH 205691, AMNH 205692, AMNH 205693). ***Cerro Largo*:** Arroyo del Cordobes 28 km NW de Cerro Chato (ZVCM 1158, ZVCM 5547, ZVCM 845, ZVCM 846, ZVCM 849, ZVCM 853, ZVCM 854, ZVCM 855); Alrededores Balneario La Laguna (ZVCM 5633); Centurión (ZVCM 5571); 20 kilometers southeast of Melo, Tacuari River, Sierra de Vaz (AMNH 205694, AMNH 205695, AMNH 205696). ***Colonia*:** S of La Lata (FMNH 29336). ***Durazno*:** 15 kilometers north northwest of San Jorge, Negro River (AMNH 205697, AMNH 205698, AMNH 205699). ***Lavalleja*:** Arroyo Sauce del Olimar Chico (MNHN 1204); Arroyo Tapes de Godoy (ZVCM 1274); Mariscala (MNHN 2492); Estancia "El Paraíso" (ZVCM 5534); Paraje Sauce de Barriga Negra, 6° Sección Pilicial del Depto de Lavalleja (ZVCM 1622); Passo Avarias (FMNH 29332); Km 162, Ruta 8 (ZVCM 5541); 12 kilometers west southwest of Zapican (AMNH 205700, AMNH 205701, AMNH 205702, AMNH 205703, AMNH 205704, AMNH 205705, AMNH 205706, AMNH 205707, AMNH 205708, AMNH 205709, AMNH 205710, AMNH 205711). ***Flores*:** Costas Rio San José, aprox. Km 140 R3 (MNHN 6275); Cuchilla Villasboas (MNHN 1927). ***Florida*:** Arroyo Sauce del Tala, 6 km SW de Sarandi Grande (MNHN 1386); Cerro Colorado (MNHN 1816). ***Maldonado*:** Sierra de los Animais (MNHN 3957); Sierra Carapé (MNHN 7374); Sierra de Canapé (MNHN 7375). ***Paysandú*:** Estancia "Lomas del Queguay" (MNHN 7351); Estancia "El Refugio" Ruta 90, 10 km antes de Algorta (ZVCM 1672, ZVCM 1673); Estancia Santa Matilde (ZVCM 1691); Fermas de Luabojú (MNHN 2316); Ruta 3 cerca de Young (MNHN 4713). ***Rio Negro*:** Alrededor de la ciudad de Fray Bentos (MNHN 3396, MNHN 688); Caiada El Tala (MNHN 6397); Fray Bentos (MNHN 6840); 15 kilometers south Paysandu, Arroyo Negro (AMNH 205712, AMNH 205713). ***Rivera*:** Rio Cuñapiró, 3 km aguas abajo del Pueblo Minas de Cuñapirú (ZVCM 8440); Ruta 5 km 418 (MNHN 3188); 5 km al W de Minas de Cuñapiru (ZVCM 607, ZVCM 848); 2 km al n de Pueblo Cuñapiru (ZVCM 843). ***Rocha*:** Bara Grande, Laguna de Castillos (ZVCM 5572); Ruta 10 km 270 (MLP 1979); San Vicente (FMNH 29331); 22 km SE Lascano (AMNH 205714); 22 kilometers southeast of Lascano (AMNH 205715). ***Salto*:** Arroyo Valentin Grande 18 km NNW de Paso del Parque (ZVCM 2010); Barra de Espinillar "Espinillar" (MNHN 683); Rio Arapey Grande, 4km W de las Termas (ZVCM 1117, ZVCM 1118); Exact locality unknown (MNHN 2675). ***San José*:** Estancia "El Relincho" (ZVCM 1777, ZVCM 1778, ZVCM 5529); Estancia "El Relincho", ruta 11 km 18 (ZVCM 5528). ***Soriano*:** Est. Santa Elena Ay° Perdido (MNHN 1120); Est. "La Madrugada" Ay° San Salvador (MNHN 685); Dolores (FMNH 29337, FMNH 29338). ***Treinta y Tres*:** Arroyo Las Pavas 14 km. SE de Cerro Chato (MNHN 2761); Arroyo Avestrus Grandes, 5° seccion policial (ZVCM 5531); Arrozol 33 (MNHN 3956); Costa de la Laguna Merin (ZVCM 54); Est: La Mimi (MNHN 2635); Ruta 8, 5 km al N TfTaes (MNHN 3187); 16 kilometers south southwest of Tacuari River mouth (AMNH 205717, AMNH 205718, AMNH 205719, AMNH 205720, AMNH 205721, AMNH 205722); 3 kilometers northwest of Mendizabal (AMNH 205723); 8 miles E of Treinta y Tres (FMNH 29335); 25 kilometers west southwest of Treinta y Tres, Olimar Chico River (AMNH 205724); Treinta y Tres (FMNH 29333); near Treinta y Tres (FMNH 29334). ***Exact locality unknown*:** UFPB 7943, UFPB 7944. ***LOCALITY UNKNOWN***: MN 1434, MNHN 5527, MNHN 5570, MNHN 6395.

***Dasypus septemcinctus cordobensis* Feijó, Patterson and Cordeiro-Estrela, 2017**

**ARGENTINA**: ***Córdoba*:** “North of Cordoba” (MACN 33.10, MACN 33.12, MACN 33.13, MACN 33.14, MACN 3311, MACN 3315, MACN 3316, MACN 3317, MACN 3318, MACN 3319, MACN 3320).
