# Supplementary material for: Taxonomic revision of the long-nosed armadillos, Genus Dasypus Linnaeus, 1758 (Mammalia, Cingulata)
Source: PLoS One. 2018 Apr 6;13(4):e0195084. doi: 10.1371/journal.pone.0195084 (PMC5889077; doi:10.1371/journal.pone.0195084)
Supplement: S2 Appendix — Characters were selected based on our direct observation of specimens in museums and on their use as diagnostic traits in previous taxonomic works on Cingulata [see references 3,6,24,26,31,33,34,36,48,49,50,51,52,53,54,55 in the main paper]. (DOCX) [file pone.0195084.s002.docx]

Appendix S2. External and cranial qualitative characters used in this study to assess the morphological variation in *Dasypus*. Characters were selected based on our direct observation of specimens in museums and on their use as diagnostic traits in previous taxonomic works on Cingulata [see references 3,6,24,26,31,33,34,36,48,49,50,51,52,53,54,55 in the main paper].

**External traits**

1. Color of the cephalic shield: (0) uniformly brownish; (1) bicolor, posterior darker and anterior yellowish.
2. Color of the carapace: (0) uniformly brownish; (1) darker dorsally and slightly yellowish in the lateral, without a clear separation; (2) darker dorsally and yellowish in the lateral, with a clear separation (based on [36]).
3. Proportion of the yellowish stripe in the lateral of the carapace: (0) absent; (1) 1/3 of the whole carapace; (2) ½ of the whole carapace; (3) 2/3 of the whole carapace (based on [36]).
4. Color of the caudal sheath: (0) ventral half paler (yellowish) and dorsal half darker (brownish or blackish); (1) ventral half light brownish and dorsal half blackish; (2) yellowish ventral half and proximal dorsal half of each ring in the tail darker and the proximal distal half of each ring paler.
5. Occipital sulcus in the cephalic shield: (0) absent; (1) present and shallow; (2) present and deep (based on [6,34,48]).
6. Occipital lobe: (0) poorly differentiated; (1) clearly notable (based on [33,48,50]).
7. Shape of the occipital lobe: (0) triangular, with well-developed posterior vertex; (1) triangular, less-developed posterior vertex, (2) semicircular (based on [6,48]).
8. Contour of the anterior border of the scapular shield: (0) rounded; (1) V-shaped (based on [33]).
9. Numbers of digit in the forefoot: (0) 4; (1) 5 (based on [3,31,33,51,54]).
10. Development of the scales at the knee: (0) poorly developed; (1) well developed (based on [33,34,51]).
11. Length of the tail in relation to the head-body length: (0) smaller; (1) similar; (2) longer (based on [26,34]).
12. First ring at the base of the tail: (0) completely surrounded the tail; (1) restricted to the dorsal half of the tail; (2) restricted to the ventral half of the tail (based on [51]).
13. Sulcus in the ventral portion of the tail: (0) absent; (1)2; (2) 3; (3) 4.
14. Shape-texture of the scales in the pelvic shield: (0) smooth; (1) rough (based on [3,31]).
15. Shape-texture of the scale in the caudal sheath: (0) Flattened; (1) keeled (based on [3,31,51]).

**Cranial traits**

1. Dorsal profile of the skull as seen in lateral view: (0) sigmoid; (1) straight (based on [55]).
2. Anterior and posterior width of the nasal bones: (0) similar; (1) wider anteriorly; (2) wider posteriorly (based on [26]).
3. Posterior contour of the suture between nasal and frontal bones: (0) straight and perpendicular to the main axis of the skull; (1) straight and anterior oriented; (2) straight and posterior oriented; (3) curved; (4) interdigitated.
4. Contour of the suture between lacrimal and frontal bones: (0) curved; (1) straight.
5. Shape of the lacrimal: (0) Rounded and well developed; (1) triangular and smaller (based on [26,33,52]).
6. Shape of the lacrimal foramen: (0) circular; (1) elongate.
7. Swollen lateral condition of the maxilla anterior to the lacrimal as seen in the dorsal view: (0) poorly developed; (1) well developed (based on [6,33,49]).
8. Position of the infraorbital foramen related to the anterior border of the lacrimal: (0) anterior; (1) similar; (2) posterior (based on [49,55]).
9. Position of the suture between maxilla and palatine related to the last upper teeth: (0) similar; (1) slightly posterior; (2) well posterior; (3) anterior (based on [24,33]).
10. Tentorial process of the parietals: (0) rectangular and prominent; (1) pentagonal and less developed (based on [3]).
11. Shape of the palate between upper teeth: (0) straight; (1) slightly concave; (2) deeply concave (based on [33,55]).
12. Lateral margin of palatine: (0) rounded; (1) erect and swollen; (2) erect and thin; (3) smoothly inclined (based on [3,26,31,33,51]).
13. Posterior border of the palatine: (0) straight; (1) convex (based on [3,31, 33, 51]).
14. Direction of the suture between jugal and squamosal: (0) vertical; (1) oblique and straight; (2) oblique and wavy; (3) horizontal.
15. Posterior extension of the nuchal crest related to the external occipital crest in the occipital bone: (0) anterior; (1) similar; (2) posterior (based on [31,51]).
16. Ventral level of the jugal related to the palatine level: (0) similar; (1) projecting downwards; (2) palatine level surpassed jugal ventral border (based on [31,51]).
17. Shape and orientation of the coronoid process: (0) vertically straight; (1) posteriorly straight; (2) posteriorly curved.
18. Angle between coronoid and condylar processes: (0) circular; (1) straight.
19. Angle between condylar and angular process: (0) shallow; (1) deep (based on [6]).
20. Angular process with a posterior projection: (0) absent; (1) present (based on [52]).
21. Posterior position of the condylar process related to the angular: (0) anterior; (1) equal; (2) posterior.
