# Supplementary material for: Taxonomic revision of the long-nosed armadillos, Genus Dasypus Linnaeus, 1758 (Mammalia, Cingulata)
Source: PLoS One. 2018 Apr 6;13(4):e0195084. doi: 10.1371/journal.pone.0195084 (PMC5889077; doi:10.1371/journal.pone.0195084)
Supplement: S3 Appendix — See Fig 2 in the main paper. (DOCX) [file pone.0195084.s003.docx]

Appendix S3. Definition of landmarks on the dorsal, ventral and lateral views of the skull used in this study. See Figure 2 in the main paper.

| Dorsal view | |
| --- | --- |
| L1 | Anteriormost point of the suture between nasals |
| L2 | Posteriormost point of the suture between nasals |
| L3 | Medial point of the suture between frontal and parietal bones |
| L4 | Medial point of the suture between parietal and occiptal bones |
| L5 | Posteriormost point of the occipital |
| L6-7 | Anteriormost point of the suture between nasal and premaxilla bones |
| L8-9 | Lateralmost point of the frontal bone horizontal to the landmark 2 |
| L10-11 | Anteriormost point of the orbit at the suture between lacrimal and frontal bones |
| L12-13 | Lateralmost point of the zygomatic aligned with the landmark 10/11 |
| L14-15 | Innermost point of the orbit in the frontal bone as seen in the dorsal view |
| L16-17 | Lateralmost point of the suture between jugal and squamosal bones |
| L18-19 | Lateralmost point of the braincase near the suture between parietal and frontal |
| L20-21 | Medial-posterior end of squamosal root of zygomatic bar |
| L22-23 | Posteriormost point of the nuchal crest of the occipital bone as seen in the dorsal view. |
|  |  |
| Ventral view | |
| L1 | Anteriormost point of the suture between nasals |
| L2 | Anteriormost point of the suture between premaxilla bones |
| L3 | Medial point of the suture between premaxilla and maxilla |
| L4 | Medial point of the suture between maxilla and palatine |
| L5 | Posteriormost point of the sutures between palatines |
| L6 | Anteriormost point of the foramen magnum along the midline of the skull |
| L7 | Posteriormost point of the occipital along the midline of the skull |
| L8 | Innermost point of the occipital condyle |
| L9 | Lateralmost point of the occipital condyle |
| L10 | Posteriomost point of the paroccipital process of the petrosal near the squamosal |
| L11 | Anteriormost point of the petrosal |
| L12 | Medial point of the anterior border of the glenoid fossa |
| L13 | Innermost point of the squamosal seen in ventral view |
| L14 | Anteriormost point of the orbit |
| L15 | Inner point of maximum curvature of the jugal |
| L16 | Lateralmost point of the suture between jugal and squamosal |
| L17 | Posteriormost point of the sutures between palatine and maxilla |
| L18 | Lateralmost point of the jugal aligned with landmark 14 |
| L19 | Posteriormost point of the upper molar alveolus |
| L20 | Anteriormost point of the suture between frontal and lacrimal |
| L21 | Point of maximum curvature along the border of the frontal as seen in lateral view |
| L22 | Anteriormost point of the anteriormost maxillary tooth alveolus |
| L23 | Lateralmost point of the nasal opening |
| L24 | Lateralmost point of the foramen magnum |
|  |  |
| Lateral view | |
| L1 | Anteriormost point of the nasal |
| L2 | Anteriormost point of the premaxilla at ventral surface |
| L3 | Dorsalmost point of the nasal aligned with landmark 2 |
| L4 | Dorsalmost point of the nasal aligned with landmark 5 |
| L5 | Anteriormost point of the anteriormost maxillary tooth alveolus |
| L6 | Anteriormost point of the lacrimal bone |
| L7 | Center of lacrimal foramen |
| L8 | Dorsalmost point of the frontal aligned with landmark 7 |
| L9 | Ventralmost point of the jugal |
| L10 | Dorsalmost point of the jugal aligned with landmark 9 |
| L11 | Dorsalmost point of the suture between jugal and squamosal |
| L12 | Ventralmost point of the suture between jugal and squamosal |
| L13 | Posteriormost point of the zygomatic bar |
| L14 | Ventralmost point of the lateral exposure of the petrosal |
| L15 | Dorsalmost point of the occipital condyle |
| L16 | Dorsalmost point of the suture between parietal and occipital |
